# The impact of financial incentives on physical activity for employees in the context of workplace health promotion: a systematic review

**DOI:** 10.1093/joccuh/uiae048

**Published:** 2024-08-19

**Authors:** Miriam Alice Vitzthum, Karsten Krüger, Christopher Weyh

**Affiliations:** Department of Exercise Physiology and Sports Therapy, Justus-Liebig-University Giessen, Kugelberg 62, 35394 Giessen, Germany; Department of Exercise Physiology and Sports Therapy, Justus-Liebig-University Giessen, Kugelberg 62, 35394 Giessen, Germany; Department of Exercise Physiology and Sports Therapy, Justus-Liebig-University Giessen, Kugelberg 62, 35394 Giessen, Germany

**Keywords:** financial incentives, physical activity, workplace health promotion, occupational health management, behavioral change, employee

## Abstract

**Objectives:**

The global increase in physical inactivity is progressively evolving into a significant health challenge. Alongside the promotion of more active leisure pursuits, elevating physical activity in the workplace has come into focus. Financial incentives are not only a popular but also a promising tool in this regard. According to behavioral economics, they are able to initiate physical activity and thus create the basis for behavioral change.

**Methods:**

The present systematic review was prepared according to the current PRISMA guidelines and with reference to the Cochrane Handbook. A systematic literature search of 6 electronic databases and 3 study registers was conducted to identify relevant literature. Both randomized controlled trials (RCTs) as well as non-RCTs were included. The Cochrane Risk-of-Bias Tool and the ROBINS-I Tool were used to assess the risk of bias of individual studies, whereas the GRADE approach was used to evaluate the quality of evidence for all studies related to physical activity outcomes. A narrative synthesis was conducted.

**Results:**

Six studies were included in the review. Among the total of 2646 participants, the average age ranged from 35.5 to 43.3 years, and women accounted for between 48.6% and 88%. Risk of bias was rated as “high” in 3 studies, “moderate” in 2, and “low” in 1. The quality of evidence was assessed as “moderate.” Four of the 6 studies reported positive effects on physical activity during the incentive period.

**Conclusions:**

Workplace health promotion incorporating financial incentives has the potential to positively impact the physical activity levels of employees.

## Key points

**What is already known on this topic:** Studies show that financial incentives can have positive effects in the form of an increase in physical activity. There is a debate about which incentive design will most effectively lead to an increase in physical activity and how sustainable the change in exercise behavior is. The present review examines the extent to which existing knowledge of the effects of financial incentives on physical activity can also be observed within the framework of occupational health management. Previous studies are analyzed and systematically summarized in order to demonstrate their quality and provide an overview of the current state of research.

**What this study adds:** It was found that the use of financial incentives in the workplace leads to an increase in physical activity in the majority of the included studies (4 out of 6), particularly in the short term. With regard to the follow-up time, a heterogeneous picture emerges, as some studies continue to show an increase in activity, whereas others show a decrease.

**How this study might affect research, practice, or policy:** This systematic review shows the great need for further studies on this topic. A focus should be placed on investigating the individualization of financial incentives in order to uncover possible causal relationships between the type, amount, and duration of the incentive and individual factors of the people studied with regard to their age, gender, and socioeconomic background. There is also a need to understand the long-term impact of financial incentives on physical activity and, moreover, on health. In addition to knowledge about the treatment effects, policymakers or companies may also be interested in cost-effectiveness.

## 1. Introduction

A “pandemic of physical inactivity”—this term has been used since 2012 to draw attention to a global problem; worldwide, more than 30% of the adult population is physically inactive, meaning that they do not meet the World Health Organization (WHO) recommendations for health-promoting physical activity.^1^^,^^2^ This has far-reaching consequences, as physical inactivity leads to an increased risk of developing noncommunicable diseases, reduced life expectancy, and a higher economic burden.^3^^,^^4^ Everyday working life is also characterized by increasing physical inactivity, which can be observed especially in high-income countries, where work is more often performed in a sedentary position due to ongoing digitalization.^1^^,^^5^ Considering that the negative effects of sedentary activities can only be offset by a high level of leisure-time activity, which is often not achieved, the promotion of a physically active lifestyle among employees in and around the work environment is becoming the focus of efforts to achieve a more active society.^1^^,^^6^ However, not all activities at the workplace are equally health-promoting. In contrast to physical activity during leisure time, which is usually characterized by aerobic activity, physical activity at work is often associated with heavy lifting and static muscle contractions. A high level of physical activity at the workplace therefore usually leads to musculoskeletal disorders, can increase blood pressure, and has been shown to be associated with an increase in all-cause-mortality.^7^^,^^8^ Therefore, increasing physical activity that is not related to work tasks is recommended for all employees, and could result in health improvements^9^ and economic benefits for employers, such as reduced absenteeism and increased productivity.^10^ Because most working adults spend one-third to two-thirds of their waking hours at work, this setting is an important place for health promotion. Therefore, workplace health promotion (WHP) is an important strategy for influencing employees’ health. WHP can be divided into 2 approaches, the individualistic and the setting-based approach. Whereas the individualistic approach to WHP uses the workplace as a venue to promote healthier lifestyles, the setting-based approach aims to create work environments that protect and promote health. Both approaches can help to increase the physical activity of employees.^11^ Examples of the settings-based approach include creating exercise-friendly work environments, such as active break rooms or providing fitness rooms. Examples of the individual approach include offering regular fitness classes, step competitions and challenges, mobile apps and digital platforms, workshops and seminars on the benefits of physical activity and introducing reward systems for regular participation in fitness programs, such as vouchers, additional vacation days, or prizes.^12^ In addition to the range of action measures to increase physical activity for employees, financial incentives have been increasingly used for this purpose in recent years.^13^ These represent an external (extrinsic) motivation that influences a person’s decision architecture in such a way that the person shows the desired behavior.^13^ The mode of action can be explained via principles of standard and behavioral economics. According to the standard economic approach, a person shows a certain behavior if the perceived costs are lower than the expected gains.^14^ In terms of physical activity, this means that a person weighs the costs (eg, time, effort) against the gains (eg, health) before making a decision for or against.^15^ An important phenomenon in this context is the present bias, which states that the further in the future a gain lies, the more it is downgraded in value.^14^^,^^15^ This means that the immediate time and physical investments of physical activity are overvalued whereas the time-delayed health benefits are undervalued. The resulting decision is often in favor of inactivity.^15^^,^^16^ From a behavioral economics perspective, a financial incentive as an immediate gain for physical activity may offset the value of the cost, thereby mitigating the present bias.^14^^,^^17^

Research shows that positive effects, like reduced sedentary behavior or reduced body mass index (BMI) on increasing physical activity can be achieved through financial incentives.^13^^,^^15^^,^^17^^,^^18^ Financial incentives can also be used to increase participation in training sessions.^15^^,^^17^ Which incentive design is most effective in increasing physical activity has not yet been clearly established.^17^ Overall, both the behavior introduced by financial incentives and a sustainable change in behavior are therefore controversially discussed. The extent to which these described findings can be transferred to the workplace setting in the context of occupational health management is currently the subject of research. However, previous results have not yet been systematically summarized.

The aim of this systematic review was therefore to investigate the effects of financial incentives on physical activity in the context of workplace health promotion. In doing so, the investigation of both short-term effects (immediately during and after the intervention period) and long-term effects (after the follow-up period) of financial incentives on physical activity was the subject of the present work.

## 2. Methods

The present systematic review was prepared according to the current Preferred Reporting Items for Systematic reviews and Meta-Analyses (PRISMA) guidelines and with reference to the Cochrane Handbook.^19^^,^^20^

### 2.1. Information sources

The systematic search to identify relevant literature was conducted in 6 electronic databases (MEDLINE [PubMed], Web of Science [Clarivate], Cochrane Central Register of Controlled Trials [CENTRAL; Wiley], SPORTDiscus [EBSCOhost], PsycINFO [EBSCOhost], and EconLit [EBSCOhost]) and 3 trial registers (International Clinical Trials Registry Platform [ICTRP], US Library of Medicine Clinical Trials Registry [CENTRAL; Wiley], and the European Union Clinical Trials Registry [www.clinicaltrialsregister.eu]). Due to the interdisciplinary nature of this review, the choice of databases and study registries was based on those with medical, sports science, health science, psychological, and economic orientations.^21^ In addition, a search of the so-called gray literature was conducted using Google Scholar (Google). For this purpose, the search syntax “workplace AND financial incentives AND physical activity” was used, with the first 50 hits included in the literature selection. In addition, a manual search was carried out using forward screening (via Web of Science and PubMed) and backward screening (reference lists of articles in the full-text selection and of related reviews.^13^^,^^15^^‑^^18^^,^^22^^‑^^25^ Furthermore, the studies that met the inclusion criteria were subjected to a further PubMed search to identify studies related to them using the “similar articles” feature.

### 2.2. Search strategy

To develop the search strategy, relevant search terms of the domains “Population” (“workplace”), “Intervention” (“financial incentives”), “Outcome” (“physical activity”), and “Study design” were connected by the Boolean operators “AND” and “OR” based on the PICO(S) process. The language filter was set to find English and German studies, as the time and cost of translating studies of other languages would exceed available capacity. In order to identify as few articles as possible that were thematically unsuitable, another search aspect was included in the search strategy and linked to the other search aspects by the operator “NOT”. In order to obtain the largest possible number of search results, no restrictions were placed on the publication period. The search strategies of all databases can be found in Appendix S1.

### 2.3. Selection criteria

To decide which articles could be included in the review, the following selection criteria were created, which are structured using the PICO(S) process.^19^ An overview of the inclusion and exclusion criteria is presented in Appendix S1. Population: studies in which the study population consisted of employees, apprentices, or trainees were included. Due to the better comparability of the studies with each other, it was important that the study population was from a high-income country.^26^ The background to this is that high-income countries tend to have a larger service sector, in which more occupations with high levels of physical inactivity can be found. Moreover, the effectiveness of financial incentives also appears to be related to income.^27^ All ages, genders, and socioeconomic backgrounds were accepted. In contrast, studies with student and patient participants were excluded because they could differ from the target group in terms of daily schedule, available financial resources, or even health conditions. In order to enable good comparability of the studies with each other, the choice of financial incentives was restricted. Accordingly, those studies were considered in which participants were offered direct financial gains in cash, via a check, or in the form of vouchers for achieving activity goals. These had to be transparent for the participants, ie, they should have been aware of the amount, duration, and frequency of the financial reward. We excluded studies in which participants did not have 100% certainty of receiving the financial gain, as is the case with lottery drawings.^28^ Comparator: both active and passive control groups were included. Active control groups refers to the promotion of physical activity independent of financial incentives (eg, via the use of pedometers). Studies without a control group were excluded because without one there is no way to attribute changes to the effect of the intervention alone.^29^ Outcomes: studies in which physical activity was the primary or secondary outcome were included in the review. Physical activity can be measured by the amount of time spent moving (in minutes per day) or the number of daily steps (eg, via smartphones or wearable devices) or by participation in sports courses, training sessions, or similar (eg, via use of a gym). Studies were included in which physical activity was recorded at the workplace only, or at the workplace in combination with leisure-time. When studies used financial incentives to reward general health-promoting behaviors (eg, weight loss, healthy eating habits), they could only be included if some of the financial incentive was used to promote achievement of activity goals. Study design: studies were included in the review if they were conducted according to a randomized controlled trial (RCT) design. This was the surest way to determine whether there was a cause-and-effect relationship between a treatment and an outcome.^30^ In addition, cluster-RCTs were also included. Since the topic of the review article is quite specific and many limitations were already set in the search for studies (see 2.2 Search strategy), non-RCTs were also included. Observational studies and studies that had not undergone a peer review process were excluded in order to keep the quality of the present review at a high scientific level.^31^

### 2.4. Selection of studies

All search results of the databases and registers used were collected in a literature management program (Citavi 6.4), and all duplicates were automatically removed. Using the selection criteria reported above, the first screening step was to review the titles and abstracts of the available search results. The remaining hits were again checked against the selection criteria in the full text. Both steps were performed independently by M.A.V. and C.W. Any disagreement was resolved in each case by discussion among themselves.

### 2.5. Data extraction process

In order to collect all relevant data from the included studies, to facilitate the assessment of the risk of bias, and to enable the synthesis of the results,^20^ the data extraction template of the Cochrane Consumer & Communication Review Group^32^ was used and adapted to the present review at appropriate points (see Appendix S2). Data were extracted by 1 author (M.A.V.), using the published study reports, and, when available, the study protocols. In case of missing study protocol, incomplete data report, and ambiguities within the study report, the study authors were contacted. Another author (C.W.) reviewed the extracted data. Again, if there was disagreement, there was discussion until agreement could be reached. A selection of the extracted data is presented in Table 1 and 2 for a better overview.

**Table 1:** Overview of the study characteristics.

**Characteristics of the study**		**Characteristics of the participants**						**Characteristics of the outcome**	
**Study ID: lead author (year)**	**Study design**	**Country**	**Setting**	**Number of arms**	**Sample size**	**Age (SD)**	**Female gender (%)**	**Type**	**Measurement**
Carrera (2020)^42^	RCT	USA	Fortune 500 company in the Midwest	IG 1	*n* = 280	M: 39.83 NM: 40.99	M: 49; NM: 54	Proportion of participants with at least a weekly gym attendance	Gym log-in records at worksite fitness centre
				IG 2	*n* = 284	M: 40.23 NM: 41.02	M: 53; NM: 54		
				IG 3	*n* = 138	NM: 40.96	NM: 55		
				IG 4	*n* = 106	M: 41.57	M: 46		
				PCG	*n* = 172	M: 41.39 NM: 41.24	M: 42; NM: 49		
Finkelstein (2016)^46^	RCT	Singapore	13 organizations + 15 worksites	IG 3	*n* = 197	35.5 (8.4)	57	Daily steps	Accelerometer at workplace and leisure time
				PCG	*n* = 201	35.6 (8.6)	56		
Hunter (2013)^44^	Quasi-experimental study	Northern Ireland	Two buildings in the main government offices	IG	*n* = 199	43.30 (9.58)	66	Minutes of PA	PA tracking system at workplace only
				ACG	*n* = 207	43.34 (9.20)	68		
Omran (2018)^27^	RCT	Canada	Canadian university	IG	*n* = 34	41 (10.39)	91.20	Daily steps	Pedometer (Yamax SW-200) at workplace and leisure time
				ACG	*n* = 35	40 (10.93)	85.70		
Patel (2016)^45^	RCT	USA	University of Pennsylvania	IG 1	*n* = 70	37.1 (10.9)	79.70	Daily steps	Smartphone app at workplace and leisure time
				ACG	*n* = 70	39.4 (12.2)	78.60		
Royer (2015)^43^	RCT	USA	Fortune 500 company in the Midwest	IG a	*n* = 363	M: 38.95 NM: 38.87	M: 50; NM: 47	Proportion of participants with at least a weekly gym attendance	Gym log-in records at worksite fitness centre
				PCG	*n* = 290	M: 40.12 NM: 39.62	M: 54; NM: 48		

Abbreviations: ACG, active control group; IG, intervention group; M, members; NM, nonmembers; PA, physical activity; PCG, passive control group; RCT, randomized controlled trial.

**Table 2:** Overview of the interventions in the included studies.

**Study ID: lead author (year)**	**Description of the IG**	**Type of FI (immediacy of payment)**	**Yield/ behavior shown**	**Limitation**	**Max. yield** ^**a**^	**Duration of intervention** ^**b**^	**Duration of follow-up period** ^**b**^
	IG 1 (“constant”): Constant FI	Voucher (n. i.)	10 USD/gym visit	Max. 1 visit/d + 2 visits/wk	160 USD	2 mo	2 mo
	IG 2 (“kickstart/front-load”): Initially higher FI than later	Voucher (n. i.)	Weeks 1-2: 25 USD/gym visit Weeks 3-8: 5 USD/gym visit		160 USD	2 mo	2 mo
	IG 3 (“constant short”): Constant FI for a shorter period	Voucher (n. i.)	10 USD/gym visit		80 USD	1 mo	3 mo
Carrera (2020)^42^	IG 4 (“extended-sporadic”): FI over a longer period with random interruptions	Voucher (n. i.)	10 USD/gym visit		160 USD	4 mo (2 mo FI)	—
Finkelstein (2016)^46^	IG 3: FI + FitBit activity tracker	Cash (every 4-6 wk)	15 SGD at 50 000-70 000 steps/wk; 30 SGD at >70 000 steps/wk	—	577.01 USD	6 mo	6 mo
Hunter (2013)^44^	IG: FI + PAL intervention	Voucher (in weeks 6 + 12)	1 GBP/30 min PA (1 min PA = 1 point; 30 points = 1 GBP)	Max. 30 points/d	85.74 USD	3 mo	—
Omran (2018)^27^	IG: FI + APT	Voucher (at the end of each week)	5 CAD/wk if at least 1 AP is completed; additional 5 CAD if all 4 APs are completed	Max. 1 AP/wk	18.34 USD	1 mo	1 mo
Patel (2016)^45^	IG 1: FI + daily feedback	Cash (daily)	1.40 USD/d with achieved step goal of 7000 steps	—	127.40 USD	3 mo	3 mo
Royer (2015)^43^	IG a: FI	Cash (after 8 wk)	10 USD/gym visit	Max. 1 visit/d + 3 visits/wk	120 USD	1 mo	5 mo

Abbreviations: AP, action plan; APT, action planning tool; CAD, Canadian dollar; FI, financial incentive; GBP, pound sterling; IG, intervention group; n. i., no information; PA, physical activity; PAL, physical activity loyalty; SGD, Singapore dollar; USD, US dollar.

a Conversion to USD using current exchange rate (2022-06-01).

b Conversion in months.

### 2.6. Data details

Data were extracted from included studies as follows.^19^^,^^20^ First, identification characteristics: author(s), publication year. Second, characteristics of the study: study objective, study design, number of trial arms, funding source. Third, characteristics of the participants: country, setting, recruitment methods, inclusion and exclusion criteria, baseline characteristics (age, gender, BMI, ethnicity, education, and physical activity), sample size (number of participants in each trial arm, lost to follow-up with rationale—if available, number of participants included in the analysis). Fourth, characteristics of the intervention: theories and objectives of the intervention, materials used (informational or physical), procedures, modalities of implementation, frequency and duration of the intervention, adjustments and modifications. Fifth, characteristics of the outcome: type of outcome (eg, daily step count), measurement tool (eg, pedometer with brand). Sixth, characteristics of the results: outcome data of the intervention and control groups at the corresponding measurement time points (post-intervention and post-follow-up).

As recommended in the Cochrane Handbook,^20^ the Template for Intervention Description and Replication (TIDieR) was used to describe the main characteristics of the intervention^33^^,^^34^ (see Appendix S2). If there were multiple possible outcomes within a study, a hierarchy developed for this purpose was used to select the outcome to be reported (see Appendix S1).

### 2.7. Risk of bias in the individual studies

To assess the risk of bias in the RCTs, the current revised version of the Cochrane Risk-of-Bias Tool (RoB 2) for randomized trials was used^35^ and the ROBINS-I tool for nonrandomized intervention studies.^36^ Each study was assessed by 1 author (M.A.V.) using the criteria “low risk,” “some concerns,” and “high risk,” whereas a second author (C.W.) independently reviewed the assessments made.^37^ Visualization of the risk-of-bias assessment was created using the robvis tool.^38^

### 2.8. Synthesis of the results

Due to a high heterogeneity of the included studies, both on a methodological level (inclusion of RCTs and nonrandomized studies of interventions, continuous and binary outcome data) and on a clinical level (regarding PICO elements, eg, active and passive comparison groups, different outcome measures and measurement time points), as well as missing data to calculate standardized effect sizes, conducting a meta-analysis was not possible.^20^^,^^39^ Alternatively, a narrative synthesis was used in which the results of individual studies are reported descriptively and synthesized in their entirety with the goal of exploring, interpreting, and finding explanations for the study findings.^40^

### 2.9. Assessment of the quality of the body of evidence

The GRADE approach was used to assess the quality of evidence from all studies on the outcome physical activity. The quality of the evidence in studies with a randomized controlled design is initially classified as high, with limitations within 5 factors (“risk of bias,” “inconsistency,” “indirectness,” “imprecision,” “publication bias”) that can lead to downgrading of quality. Observational studies, on the other hand, are initially assessed with a low quality score, but can be upgraded based on 3 characteristics (“large effect,” “dose–response,” “all plausible confounding”). In order to apply the GRADE approach, even when a meta-analysis is not performed but data are summarized in a narrative manner, as in the present review, the guidance of Murad et al^41^ was used in assessing the quality of evidence. This procedure was performed by one author (M.A.V.), and a second author (C.W.) verified this independently.

**Figure 1:**
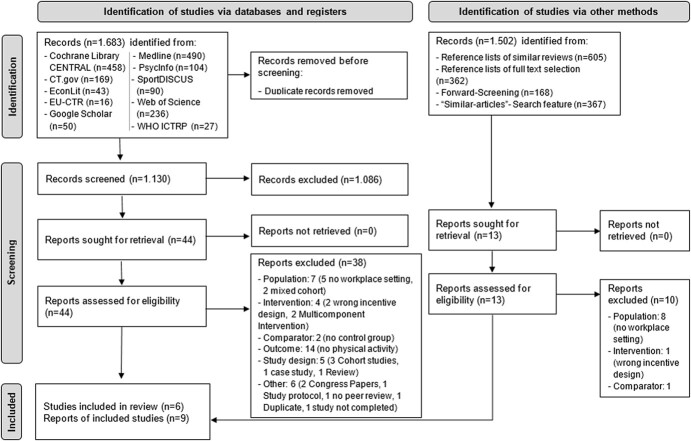
PRISMA flowchart.

**Figure 2:**
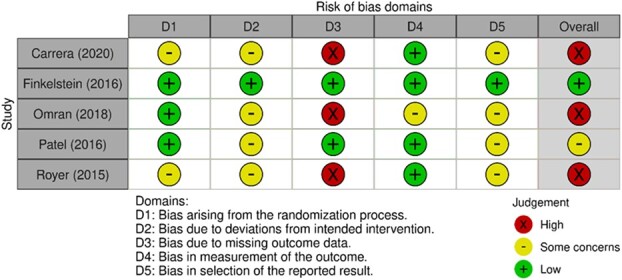
Risk of bias 2 (ROB 2) graph.

**Figure 3:**
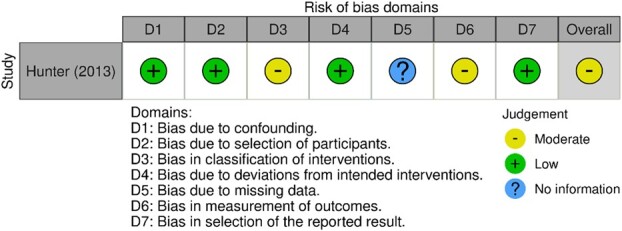
Risk of bias in nonrandomized studies of interventions (ROBINS-I) graph.

## 3. Results

### 3.1. Study selection

A total of 1683 articles were identified by the search carried out on June 17, 2022 in the described databases and registers. After removing duplicates and screening for title and abstract, 44 full-text study reports were screened for eligibility, of which 38 were excluded. In the manual search (see above) 1502 additional articles could be identified and screened. In this way, the full texts of 13 additional study reports were checked. Ten of these were excluded. Thus, 6 studies were identified for review, of which 2 studies represented 5 reports (ie, 9 study reports in total). The flow diagram provided in Figure 1 provides an overview of the number of search results after each screening step and the reason for the elimination of the respective articles.

### 3.2. Study characteristics

Table 1 describes the characteristics of the 6 included studies. The population (*n* = 2646) consisted of employees of companies, universities, and government agencies. Three studies reported that employees performed mostly sedentary office jobs. The average age of the populations ranged from 35.5 to 43.3 years. The outcome was reported on daily step count, the proportion of participants with at least 1 gym visit per week, and minutes of physical activity per day. The outcome measurements took place after the intervention and after the follow-up period. The intervention duration of the individual studies varied between 1 and 6 months, and the duration of the follow-up period between 2 and 12 months. In 2 studies, the outcome was investigated depending on the membership status before the intervention (nonmembers or members). Three studies measured physical activity in the workplace setting.^42^^‑^^44^ The 3 other studies recorded activity throughout the day, both at workplace and during leisure time.^27^^,^^45^^,^^46^  Table 2 summarizes the individual intervention components of all studies to enable a clear comparison between the studies.^19^

### 3.3. Risk of bias

The results of the risk-of-bias assessment are shown in Figures 2 and 3. The studies assessed using the RoB 2 tool (Figure 2) were rated in the overall assessment as follows: 1 study with a low risk for bias, 2 with some concerns, and 3 with a high risk. To make it easier to understand which factors led to the overall assessment at each study level, the assessment of each domain is presented in more detail below.^19^ Detailed justifications can be found in Appendix S3.

In domain 1 (bias due to the randomization process), 2 studies provided no information about allocation sequence and about allocation sequence concealment, which is why they were rated as “some concerns.” Because both participants and study coordinators could not be blinded due to the nature of the interventions, in domain 2 the classification “some concerns” occurred in 4 of the 5 studies assessed by RoB 2 tool. A high-risk assessment could be dispensed with because an intention-to-treat (ITT) analysis ensured that all participants were analyzed in the group to which they were initially assigned.^36^ Only in 1 study could a low-risk assessment be made by examining the study protocol. A high risk of bias was found in the third domain in 3 studies. This was because despite the fact that less than 95% of the outcome data were available, the authors had not conducted analyses to show how the lack of these may bias the results.^35^ Likewise, no reasons were provided for participant dropouts. A further classification of “some concerns” was made in domain 4 (bias due to outcome measurement) for a study in which there were doubts about the appropriateness of the measurement methodology. Due to missing study protocols, no statement could be made about the intended outcome measures and analysis methods in domain 5 (bias due to selection of the reported outcome). This resulted in a classification of “some concerns” for 4 of the 5 studies.

The study evaluated with the ROBINS-I tool was classified as moderate risk in the overall assessment (see Figure 3). This means that the study was robust, but not “comparable to a well-conducted RCT.”^36^ Reasons for this classification were due to a missing study protocol (moderate risk in domain 3) and the fact that participants could not be blinded (moderate risk in domain 6). Lack of information about the dropout of the participants as well as about the handling of the missing data led to the classification of “no information” in domain 5.

### 3.4. Results of the individual studies

The 6 included studies could be divided into 2 studies with financial incentives for gym visits^42^^,^^43^ and 4 studies with financial incentives for achieving activity goals.^27^^,^^44^^‑^^46^ Financial incentives for gym visits were given in the form of vouchers or cash, with maximum incentive amounts ranging from 80 US dollars (USD) to 160 USD. Both studies differentiated participants into nonmembers or members according to membership status before the intervention and compared their intervention groups with passive control groups who were not motivated financially or otherwise to increase their physical activity. In both studies, it was shown that the proportion of nonmembers or members of an intervention group who exercised at least once a week in the gym was significantly higher than the proportion of the control groups. Although this could be observed for all intervention groups at the time after the intervention, regardless of the varying level and duration of the incentive, an inconsistent picture emerged after the follow-up phase: the constant financial incentive of 10 USD per gym visit over a longer period of 8 weeks also led to increased use of the gym in the long term (the nonmembers increased use by 3.6 percentage points compared with the control group, the members increased use by 9.3 percentage points; *P* < .05, *P* < .001, respectively). However, due to the lack of statistical data, it was not possible to make a statement about the size of the effect.

The studies that financially rewarded the achievement of predetermined activity goals used daily as well as weekly step goals, weekly action plans to be completed, and minutes of physical activity as criteria for the financial incentives. These were issued in the form of vouchers as well as cash. Three of the 4 studies compared their intervention groups with active control groups that underwent the same intervention only without financial incentives. Of the 3 studies with active control groups, only one^27^ reported a positive effect of financial incentives on physical activity at both measurement time points: During the 4-week follow-up period, the intervention group had an increase from baseline of 1793 (SD = 2408.72) daily steps, which is higher than the control group’s 686 (SD = 2887.62) daily steps. Another study with a passive control group^46^ found higher physical activity in the intervention group in the form of 1050 steps per day (95% CI, 600-1490) compared with the control group after a 6-month intervention period (*P* < .0001). Even after the 12-month follow-up period, a difference of 500 steps per day (95% CI, 50-960) was observed between the intervention and the control group (*P* < .05). An overview of the individual study results and their statistical significance is provided in Table 3.

**Table 3:** Overview of the individual study results.

**Study ID**	**Intervention**	**Type of outcome**	**Outcome measurement**	**Measurement time points: after intervention; after follow-up**	**Results**
	IG 1: Constant FI	Proportion of participants with at least a weekly gym attendance	Gym log-in records	2 mo	NM↑ M↑
				4 mo	NM↑ M↑
	IG 2: Initially higher FI than later			2 mo	NM↑ M↑
				4 mo	NM↔ M↔
	IG 3: Constant FI for a shorter period			1 mo	NM↑
				4 mo	NM↔
	IG 4: FI over a longer period with random interruptions			4 mo	M↑
Carrera (2020)^42^				—	—
Finkelstein (2016)^46^	FI + activity tracker	Daily steps	Accelerometer	6 mo	↑ (+1050 steps/d [95% CI, 600-1490])
				12 mo	↑ (+500 steps/d [95% CI, 50-960])
Hunter (2013)^44^	FI + PAL intervention	Minutes of PA	PA-tracking system	3 mo	↔
				—	—
Omran (2018)^27^	FI + APT	Daily steps	Pedometer	1 mo	↔
				2 mo	↑ (+1793 steps/d; SD = 2407.78)
Patel (2016)^45^	FI + daily feedback	Daily steps	Smartphone app	3 mo	↔
				6 mo	↔
Royer (2015)^43^	FI	Proportion of participants with at least a weekly gym attendance	Gym log-in records	1 mo	NM↑ M↑
				6 mo	NM↑ M↔

Abbreviations: APT, action planning tool; FI, financial incentives; IG, intervention group; M, members; NM, nonmembers; PA, physical activity; PAL, physical activity loyalty; ↔, no effect on physical activity; ↑, increase in physical activity.

### 3.5. Results of the syntheses

Across all studies, the majority (4 out of 6) reported statistically significant results regarding the impact of financial incentives on physical activity during the incentive period.^27^^,^^42^^,^^43^^,^^46^ Given the overall small number of included studies, the 2 studies whose results are not statistically significant represent a non-negligible, relatively high proportion of the total number of articles examined, accounting for one-third of the total. For this reason, a mixed picture of results can be assumed here, although statistically nonsignificant results do not necessarily mean that there is no effect.^47^ A closer look at the results of the follow-up periods reveals an even more heterogeneous picture: Here, only the intervention groups from the studies by Finkelstein et al and Omran et al, as well as intervention group (IG) 1 from the study by Carrera et al and the nonmembers of the IG from the study by Royer et al show statistically significant results.^27^^,^^42^^,^^43^^,^^46^

In summary, it can be stated that short-term effects of financial incentives on physical activity can be observed in the context of occupational health management, although their unambiguity is questionable.

### 3.6. Assessment of the quality of the body of evidence

The quality of the evidence was rated as “moderate”. The downgrading of the quality of evidence by 1 level is mainly because the risk of bias was rated as “severe” across all studies.^48^ Among others, 2 studies stood out as having concerns or high risk of bias across all criteria of this domain, in addition to having relatively large sample sizes (*n* = 1633).^42^^,^^43^ Accordingly, the proportion of studies with high risk of bias was sufficient to weaken confidence in the results and influence interpretation of the results. A summary overview regarding the risk of bias of the studies is presented in Appendix S3. In addition, it should be noted that the quasi-experimental study by Hunter et al^44^ was more similar to a cluster-randomized trial than an observational study, which means that the initial study quality was classified as moderate rather than low due to the study design,^49^ and thus it is included in the assessment of GRADE ranges.

## 4. Discussion

### 4.1. Summary of evidence

The aim of this systematic review was to investigate the effects of financial incentives on physical activity for employees in the context of workplace health promotion. Both short-term and long-term effects of financial incentives on physical activity were considered. The results of the 6 studies included to explore this topic showed a heterogeneous picture, based on moderate evidence. Through the use of financial incentives, an increase in physical activity and visits to fitness facilities was initially recorded in most cases. With regard to the follow-up time, some studies continued to show an increased activity or participation rate, whereas others showed a decrease. Despite the difficulty in making a definitive statement about the topic examined in this review, financial incentives appear to increase physical activity, especially in the short term.

Adams et al^28^ developed different domains for describing interventions that are intended to influence the health behavior of individuals with financial incentives. A crucial factor seems to be the type and amount of the incentive as well as the immediacy between the performance of the required criterion and the payment of the incentive. In addition, a possible influence of the duration of the interventions is discussed. When assessing the incentive level as an influencing factor, the economic background of the participants must also be taken into account. For this purpose, the purchasing power-adjusted gross domestic product (GDP) per capita of those countries in which the studies were carried out is taken and related to the maximum financial incentive to be received from the respective study. For better comparability, the corresponding national currencies are converted into USD. It is notable that in a country like Canada, with a comparatively low purchasing power-adjusted GDP per capita of 47 903 International Dollars, even a relatively small financial incentive totaling 25 Canadian dollars (CAD; about 18.34 USD) had a beneficial effect on physical activity.^27^ In contrast, in a country such as Singapore, where purchasing power-adjusted GDP per capita is much higher at 106 032 International Dollars, a response to the intervention was achieved with a higher total financial incentive of 780 Singapore dollars (SGD; approximately 577.01 USD).^46^ From these 2 comparisons, it can be deduced that financial incentives should be adjusted depending on the level of GDP per capita adjusted for purchasing power in order to represent a relevant incentive for the target population. However, a comparison of the 3 studies conducted in the United States^42^^,^^43^^,^^45^ shows that despite the same purchasing power-adjusted GDP per capita of 61 505 International Dollars and similar financial incentives of 80-160 USD, different results could be observed. There was also no effect in the study by Hunter et al,^44^ although the UK’s purchasing power-adjusted GDP per capita of 45 839 International Dollars is lower than that of Canada, despite the maximum financial incentive at 70 GBP (about 85.74 USD) being higher than those in the study by Omran et al.^27^ The assumption of Adams et al,^28^ that people with low incomes are more likely to be motivated to change their health behavior by lower financial incentives than those with high incomes, can therefore neither be refuted nor supported by the included studies. Other studies and reviews also controversially report incentive level as an influencing factor. Mitchell et al^15^ found in the subgroup analysis of their review that higher incentives also led to greater effects on physical activity. This contrasts with the observation of Giles et al^16^ that as the incentive value increases, the effect of the incentives diminishes.

The immediacy of a financial incentive describes how quickly the incentives are offered after the behavior. As described (see Table 2), this varied greatly between studies. Within the included studies no connection could be established between the immediacy of the financial incentive and the physical activity. This contrasts with the observations made by Adams et al^50^ in their study of financial incentives. Here, participants in the intervention group with immediate rewards showed a significantly higher step count compared with baseline than participants with delayed rewards.

Similarly, no consistent conclusion can be drawn from the included studies for the duration of the interventions during which financial incentives were given. It becomes clear that a response to the intervention was observed both at a shorter duration of 1 or 2 months^27^^,^^42^^,^^43^ and at a longer one of 6 months.^46^ Mitchell et al^15^ showed that the effects of financial incentives on physical activity were greater the longer the interventions were carried out. But for the studies included in this review, such a relationship of effect size with duration of intervention cannot be replicated, for the reasons stated above. In addition, Mitchell et al^15^ found that 3 months may not be sufficient to form new habits—a statement that cannot be supported based on the follow-up results of the included studies, as studies with a shorter intervention duration also showed statistically significant results after follow-up measurement. The review included studies with varying activity goals and types of physical activity. The results of 2 studies on gym log-in frequency were predominantly positive. However, in the other 4 studies, an increase in daily steps or activity due to financial incentives was reported in only half. This suggests that a single and short-time weekly goal, such as a gym visit combined with a financial incentive, might be more acceptable to employees. It must be emphasized, however, that from the log-in frequencies alone, we do not obtain information about the duration and intensity of physical activity, which would be important from a health perspective. Therefore, incentives that only target participation frequency are considered questionable.^13^ The review therefore cannot conclude whether a specific type of physical activity can be better increased by financial incentives. In addition to knowledge about the treatment effects, policymakers or companies may also be interested in cost-effectiveness. However, this point was addressed only by Hunter et al.^44^. The authors were unable to determine any cost-effectiveness as a result of incentives due to a change in absenteeism. Other reviews are also unable to make a clear recommendation due to limited evidence.^25^ Only the effects of financial incentives were examined in this review. Other reward systems, as described above, could be effective, especially since various target groups within a setting respond differently to incentives, as could be shown for women and men.^51^ Further research is necessary.

### 4.2. Limitations at study level

When interpreting the results, it should be noted that the body of evidence for all included studies was downgraded by 1 level to moderate. Concerns exist, firstly, because of the serious impact of risk of bias, which is mainly due to the lack of blinding, incomplete accounting of patients and outcome events, and selective reporting. Secondly, concerns are due to the fact that the quality of the evidence could not be assessed in the areas of “inconsistency” and “insufficient precision.” This creates some uncertainty in the evidence that should be considered in questions of applicability.

### 4.3. Limitations at review level

The following limitations of the review should be considered. Firstly, most of the included studies lacked appropriate study protocols—queries to the study authors regarding this and missing data remained mostly unanswered. Secondly, despite the extensive literature search in databases, registers, and the gray literature, it cannot be assumed with certainty that all relevant publications were found. Furthermore, due to a lack of financial and time resources, only studies in English and German could be included. Thirdly, because of the specific topic and the small number of studies conducted, nonrandomized controlled studies were also included in the review. Fourthly, a relatively large variation in the study population (eg, in terms of age and gender) could be observed. It should also be noted that both the direct (eg, daily step count via pedometer) and indirect (gym visits via log-in data) methods of measurement of the physical activity outcome were compared as well as different types of physical activity interventions. Moreover, half of the interventions only measured physical activity at the workplace, whereas the other 3 studies measured physical activity both at the workplace and during leisure time. In addition, there are limitations in the synthesis of results due to the fact that a meta-analysis could not be performed because of the high heterogeneity of the individual studies and a lack of data to calculate standardized effect sizes. In turn, interpretation of the results through narrative synthesis did not allow us to draw conclusions about the size and direction of the effect. Finally, the lack of a protocol for the transparent presentation of all steps performed in the review process as well as the lack of preregistration can be considered as a limitation. Apart from that, all other recommendations of the PRISMA guidelines were followed and are documented in detail and comprehensively in the Appendix S4.

## 5. Conclusions

The use of financial incentives in the workplace appears to have the potential to increase physical activity, especially in the short term. Characteristics of the population, such as gender and economic background, as well as the type of incentive design and their complex interactions with each other could influence effectiveness. It is still unclear which incentive design in which population leads to primarily sustainable improvements in physical activity behavior. This should be the subject of future research, as should the question of the cost-effectiveness of financial incentives.

## Supplementary Material

Web_Material_uiae048

## Data Availability

The data underlying this article will be shared on reasonable request to the corresponding author.
