# Supplementary material for: The impact of financial incentives on physical activity for employees in the context of workplace health promotion: a systematic review
Source: J Occup Health. 2024 Aug 19;66(1):uiae048. doi: 10.1093/joccuh/uiae048 (PMC11662443; doi:10.1093/joccuh/uiae048)
Supplement: Web_Material_uiae048 [file web_material_uiae048.zip › Appendix 1 search strategies.docx]

Appendix 1. Search strategies

Appendix 1. Tab. 1. Search strategy MEDLINE (Pubmed)

| Search aspect | Search terms |
| --- | --- |
| Search aspect 1  Population  /Problem | Workplace [mesh] OR Occupational health services [mesh] OR „Workplace health management“ [tiab] OR „Worksite health management“ [tiab] OR „Occupational health management“ [tiab] OR „Occupational health care“ [tiab] OR „Occupational health“ [tiab] OR staff [tiab] OR job [tiab] OR labour [tiab] OR work [tiab] OR workplace-based [tiab] OR „workplace setting*“ [tiab] OR Occupation* [tiab] OR Workplace* [tiab] OR Worksite [tiab] OR Workforce [tiab] OR Employee* [tiab] OR Worker* [tiab] OR Employer* [tiab] OR Trainee* [tiab] OR Apprentice* [tiab] |
| Search aspect 2  Intervention | reward [mesh] OR financial support [mesh] OR health benefit plans, employee [mesh] OR motivation [mesh] OR Lotter* [tiab] OR Voucher* [tiab] OR Rebate* [tiab] OR Nudg* [tiab] OR Gift* [tiab] OR „Token econom*“ [tiab] OR „Incentive design*“ [tiab] OR ((financial OR material OR monetary OR economic OR cash) AND (incentive* OR motivation* OR value* OR reward* OR bonus* OR compensation* OR gratification* OR payment* OR support*)) [tiab] |
| Search aspect 3  Outcome | Exercise [mesh] OR Sports [mesh] OR recreation [mesh] OR Physical fitness [mesh] OR Physical conditioning, human [mesh] OR Weight loss [mesh] OR „Physical exercise*“ [tiab] OR „Sedentary behavio*“ [tiab] OR Exercise* [tiab] OR Sport* [tiab] OR Activ* [tiab] OR Walk* [tiab] OR Recreation* [tiab] OR Fitness [tiab] OR ((exercise OR course) AND (participation OR adherence OR attendance)) [tiab] OR (commut* AND (active OR bicycl* OR bike OR biking OR cycl* OR walk*)) [tiab] |
| Search aspect 4  Study design | (Random* AND (alloc* OR assign*)) [tiab] OR (Controlled AND (stud* OR trial*)) [tiab] OR (Blind AND (single OR double OR treble OR triple)) [tiab] OR Randomi* [tiab] OR Randomly [tiab] OR placebo [tiab] OR „Controlled clinical trial*“ [tiab] OR Cluster [tiab] OR rct [tiab] OR crct [tiab] OR cct [tiab] |
| Search aspect 5  Exclusion | Child* [ti] OR Teenage* [ti] OR Parent* [ti] OR Pregnant* [ti] OR Juvenile* [ti] OR Student* [ti] |
| (Search aspect 1 AND search aspect 2 AND search aspect 3 AND search aspect 4) NOT search aspect 5 | |
| Search fields:  [mesh] = Thesaurus term including narrower  [tiab] = Title/Abstract | |
| Filter:  Language: english, german | |

Appendix 1. Tab. 2*. Search strategy Cochrane Library CENTRAL (Wiley)*

| Search aspect | Search terms |
| --- | --- |
| Search aspect 1  Population /Problem | MeSH „Workplace“ expl. OR MeSH „Occupational health services“ expl. OR „Workplace health management“ OR „Worksite health management“ OR „Occupational health management“ OR „Occupational health care“ OR „Occupational health“ OR (staff:ti OR :ab) OR (job:ti OR :ab) OR (labour:ti OR :ab) OR (work:ti OR :ab) OR (workplace-based:ti OR :ab) OR ((workplace NEXT setting?):ti OR :ab) OR (Occupation*:ti OR :ab) OR (Workplace?:ti OR :ab) OR (Worksite:ti OR :ab) OR (Workforce:ti OR :ab) OR (Employee?:ti OR :ab) OR (Worker?:ti OR :ab) OR (Employer?:ti OR :ab) OR (Trainee?:ti OR :ab) OR (Apprentice?:ti OR :ab) |
| Search aspect 2  Intervention | MeSH „Reward“ expl. OR MeSH „financial support“ expl. OR MeSH „health benefit plans, employee“ expl. OR MeSH „motivation“ expl. OR (Lotter*:ti OR :ab) OR (Voucher?:ti OR :ab) OR (Rebate?:ti OR :ab) OR (Nudg*:ti OR :ab) OR (Gift?:ti OR :ab) OR ((Token NEXT econom*):ti OR :ab) OR ((Incentive NEXT design?):ti OR :ab) OR (((financial OR material OR monetary OR economic OR cash) NEXT (incentive? OR motivation? OR value? OR reward? OR bonus* OR compensation? OR gratification? OR payment? OR support?)):ti OR :ab) |
| Search aspect 3  Outcome | MeSH „Exercise“ expl. OR MeSH „Sports“ expl. OR MeSH „recreation“ expl. OR MeSH „Physical fitness“ expl. OR MeSH „Physical conditioning, human“ expl. OR MeSH „Weight loss“ expl. OR ((Physical NEXT exercise?):ti OR :ab) OR ((Sedentary NEXT behavio?r):ti OR :ab) OR (Exercise? :ti OR :ab) OR (Sport? :ti OR :ab) OR (Activ*:ti OR :ab) OR (Walk*:ti OR :ab) OR (Recreation*:ti OR :ab) OR (Fitness:ti OR :ab) OR (((exercise OR course) near/1 (participation OR adherence OR attendance)):ti OR :ab) OR ((commut* near/1 (active OR bicycl* OR bik* OR cycl* OR walk*)):ti OR :ab) |
| Search aspect 4  Study design | ((Random* next (alloc* OR assign*)):ti OR :ab) OR ((Controlled next (stud* OR trial*)):ti OR :ab) OR ((Blind next (single OR double OR treble OR triple)):ti OR :ab) OR (Randomi*:ti OR :ab) OR (Randomly:ti OR :ab) OR (placebo:ti OR :ab) OR ((Controlled NEXT clinical NEXT trial?):ti OR :ab) OR (Cluster:ti OR :ab) OR (rct:ti OR :ab) OR (crct:ti OR :ab) OR (Cct:ti OR :ab) |
| Search aspect 5  Exclusion | Child*:ti OR Teenage*:ti OR Parent*:ti OR Pregnant*:ti OR Juvenile*:ti OR Student*:ti |
| (Search aspect 1 AND search aspect 2 AND search aspect 3 AND search aspect 4) NOT search aspect 5 | |
| Search fields:  MeSH „“ expl. = MeSH term explode all tress;  :ti OR :ab = Title OR Abstract | |
| Filter:  Language: english, german | |

Appendix 1. Tab. 3. Search strategy EconLit (EBSCOhost)

| Search aspect | Search terms |
| --- | --- |
| Search aspect 1  Population /Problem | TI/AB („Workplace intervention“) OR TI/AB („Occupational health services“) OR TI/AB („Workplace health management“) OR TI/AB („Worksite health management“) OR TI/AB („Occupational health management“) OR TI/AB („Occupational health care“) OR TI/AB („Occupational health“) OR TI/AB (staff) OR TI/AB (job) OR TI/AB (labour) OR TI/AB (work) OR TI/AB (workplace-based) OR TI/AB („workplace setting#“) OR TI/AB (Occupation*) OR TI/AB (Workplace#) OR TI/AB (Worksite) OR TI/AB (Workforce) OR TI/AB (Employee#) OR TI/AB (Worker#) OR TI/AB (Employer#) OR TI/AB (Trainee#) OR TI/AB (Apprentice#) |
| Search aspect 2  Intervention | TI/AB (Lotter*) OR TI/AB (Voucher#) OR TI/AB (Rebate#) OR TI/AB (Nudg*) OR TI/AB (Gift#) OR TI/AB („Token econom*“) OR TI/AB („Incentive design#“) OR TI/AB ((financial OR material OR monetary OR economic OR cash) N1 (incentive# OR motivation# OR value# OR reward# OR bonus* OR compensation# OR gratification# OR payment# OR support#)) |
| Search aspect 3  Outcome | TI/AB („Health Behavior“) OR TI/AB („Physical fitness“) OR TI/AB ("Physical activity“) OR TI/AB („Weight loss“) OR TI/AB („Physical exercise#“) OR TI/AB („Sedentary behavio#r“) OR TI/AB (Exercise#) OR TI/AB (Sport#) OR TI/AB (Activ*) OR TI/AB (Walk*) OR TI/AB (Recreation*) OR TI/AB (Fitness) OR TI/AB ((exercise OR course) N1 (participation OR adherence OR attendance)) OR TI OR AB (commut* N1 (ative OR bicycl* OR bik* OR cycl* OR walk*)) |
| Search aspect 4  Study design | TI/AB (Random* N1 (alloc* OR assign*)) OR TI/AB (Controlled N1 (stud* OR trial*)) OR TI/AB (Blind N1 (single OR double OR treble OR triple)) OR TI/AB (Randomi*) OR TI/AB (Randomly) OR TI/AB (placebo) OR TI/AB („Controlled clinical trial*“) OR TI/AB (Cluster) OR TI/AB (rct) OR TI/AB (crct) OR TI/AB (cct) |
| Search aspect 5  Exclusion | TI (Child*) OR TI (Teenage#) OR TI (Parent#) OR TI (Pregnant#) OR TI (Juvenile#) OR TI (Student#) |
| (Search aspect 1 AND search aspect 2 AND search aspect 3 AND search aspect 4) NOT search aspect 5 | |
| Search fields:  TI/AB = TI (Title) OR AB (Abstract) | |
| Filter:  Language: english, german | |

Appendix 1. Tab. 4. Search strategy PsycINFO (Ebscohost)

| Search aspect | Search terms |
| --- | --- |
| Search aspect 1  Population /Problem | MH „workplace intervention“ OR TI/AB („Workplace health management“) OR TI/AB („Worksite health management“) OR TI/AB („Occupational health management“) OR TI/AB („Occupational health care“)  OR TI/AB („occupational health service#“) OR TI/AB („Occupational health“) OR TI/AB (staff) OR TI/AB (job) OR TI/AB (labour) OR TI/AB (work) OR TI/AB (workplace-based) OR TI/AB („workplace setting#“) OR TI/AB (Occupation*) OR TI/AB (Workplace#) OR TI/AB (Worksite) OR TI/AB (Workforce) OR TI/AB (Employee#) OR TI/AB (Worker#) OR TI/AB (Employer#) OR TI/AB (Trainee#) OR TI/AB (Apprentice#) |
| Search aspect 2  Intervention | MH „rewards+“ OR MH „incentives+“ OR TI/AB (Lotter*) OR TI/AB (Voucher#) OR TI/AB (Rebate#) OR TI/AB (Nudg*) OR TI/AB (Gift#) OR TI/AB („Token econom*“) OR TI/AB („Incentive design#“) OR TI/AB ((financial OR material OR monetary OR economic OR cash) N1 (incentive# OR motivation# OR value# OR reward# OR bonus* OR compensation# OR gratification# OR payment# OR support#)) |
| Search aspect 3  Outcome | MH „Exercise+“ OR MH „Sports+“ OR MH „recreation+“ OR MH „Physical fitness“ OR MH „Weight loss“ OR MH "Physical activity+“ OR TI/AB („Physical exercise#“) OR TI/AB („Sedentary behavio#r“) OR TI/AB (Exercise#) OR TI/AB (Sport#) OR TI/AB (Activ*) OR TI/AB (Walk*) OR TI/AB (Recreation*) OR TI/AB (Fitness) OR TI/AB ((exercise OR course) N1 (participation OR adherence OR attendance)) OR TI/AB (commut* N1 (ative OR bicycl* OR bik* OR cycl* OR walk*)) |
| Search aspect 4  Study design | TI/AB (Random* N1 (alloc* OR assign*)) OR TI/AB (Controlled N1 (stud* OR trial#)) OR TI/AB (Blind N1 (single OR double OR treble OR triple)) OR TI/AB (Randomi#ed) OR TI/AB (randomisation) OR TI/AB (Randomly) OR TI/AB (placebo) OR TI/AB („Controlled clinical trial*“) OR TI/AB (Cluster) OR TI/AB (rct) OR TI/AB (crct) OR TI/AB (cct) |
| Search aspect 5  Exclusion | TI (Child*) OR TI (Teenage#) OR TI (Parent#) OR TI (Pregnant#) OR TI (Juvenile#) OR TI (Student#) |
| (Search aspect 1 AND search aspect 2 AND search aspect 3 AND search aspect 4) NOT search aspect 5 | |
| Search fields:  MH = thesaurus term; + = including narrower;  TI/AB = TI (Title) OR AB (Abstract) | |
| Filter:  Language: english, german | |

Appendix 1. Tab. 5. Search strategy SPORTDiscus (EBSCOhost)

| Search aspect | Search terms |
| --- | --- |
| Search aspect 1  Population /Problem | MH „Occupational health services“ OR TI/AB („Workplace health management“) OR TI/AB („Worksite health management“) OR TI/AB („Occupational health management“) OR TI/AB („Occupational health care“) OR TI/AB („Occupational health“) OR TI/AB (staff) OR TI/AB (job) OR TI/AB (labour) OR TI/AB (work) OR TI/AB (workplace-based) OR TI/AB („workplace setting#“) OR TI/AB (Occupation*) OR TI/AB (Workplace#) OR TI/AB (Worksite) OR TI/AB (Workforce) OR TI/AB (Employee#) OR TI/AB (Worker#) OR TI/AB (Employer#) OR TI/AB (Trainee#) OR TI/AB (Apprentice#) |
| Search aspect 2  Intervention | MH „Motivation (Psychology)+“ OR TI/AB (Lotter*) OR TI/AB (Voucher#) OR TI/AB (Rebate#) OR TI/AB (Nudg*) OR TI/AB (Gift#) OR TI/AB („Token econom*“) OR TI/AB („Incentive design#“) OR TI/AB ((financial OR material OR monetary OR economic OR cash) N1 (incentive# OR motivation# OR value# OR reward# OR bonus* OR compensation# OR gratification# OR payment# OR support#)) |
| Search aspect 3  Outcome | MH „Exercise+“ OR MH „Sports+“ OR MH „recreation+“ OR MH „Physical fitness+“ OR MH "Physical activity“ OR MH „Weight loss+“ OR TI/AB („Physical exercise#“) OR TI/AB („Sedentary behavio#r“) OR TI/AB (Exercise#) OR TI/AB (Sport#) OR TI/AB (Activ*) OR TI/AB (Walk*) OR TI/AB (Recreation*) OR TI/AB (Fitness) OR TI/AB ((exercise OR course) N1 (participation OR adherence OR attendance)) OR TI OR AB (commut* N1 (ative OR bicycl* OR bik* OR cycl* OR walk*)) |
| Search aspect 4  Study design | TI/AB (Random* N1 (alloc* OR assign*)) OR TI/AB (Controlled N1 (stud* OR trial*)) OR TI/AB (Blind N1 (single OR double OR treble OR triple)) OR TI/AB (Randomi*) OR TI/AB (Randomly) OR TI/AB (placebo) OR TI/AB („Controlled clinical trial*“) OR TI/AB (Cluster) OR TI/AB (rct) OR TI/AB (crct) OR TI/AB (cct) |
| Search aspect 5  Exclusion | TI (Child*) OR TI (Teenage#) OR TI (Parent#) OR TI (Pregnant#) OR TI (Juvenile#) OR TI (Student#) |
| (Search aspect 1 AND search aspect 2 AND search aspect 3 AND search aspect 4) NOT search aspect 5 | |
| Search fields:  MH = thesaurus term; + = including narrower;  TI/AB = TI (=Title) OR AB (=Abstract) | |
| Filter:  Language: english, german | |

Appendix 1. Tab. 6. Search strategy Web Of Science (Clarivate)

| Search aspect | Search terms |
| --- | --- |
| Search aspect 1  Population /Problem | TI=/AB=(„Occupational health service$“) OR TI=/AB=(„Workplace health management“) OR TI=/AB=(„Worksite health management“) OR TI=/AB=(„Occupational health management“) OR TI=/AB=(„Occupational health care“) OR TI=/AB=(„Occupational health“) OR TI=/AB=(staff) OR TI=/AB=(job) OR TI=/AB=(labour) OR TI=/AB=(work) OR TI=/AB=(workplace-based) OR TI=/AB=(„workplace setting$“) OR TI=/AB=(Occupation*) OR TI=/AB=(Workplace$) OR TI=/AB=(Worksite) OR TI=/AB=(Workforce) OR TI=/AB=(Employee$) OR TI=/AB=(Worker$) OR TI=/AB=(Employer$) OR TI=/AB=(Trainee$) OR TI=/AB=(Apprentice$) |
| Search aspect 2  Intervention | TI=/AB=(Lotter*) OR TI=/AB=(Voucher$) OR TI=/AB=(Rebate$) OR TI=/AB=(Nudg*) OR TI=/AB=(Gift$) OR TI=/AB=(„Token econom*“) OR TI=/AB=(„Incentive design$“) OR TI=/AB=((financial OR material OR monetary OR economic OR cash) NEAR/0 (incentive$ OR motivation$ OR value$ OR reward$ OR bonus* OR compensation$ OR gratification$ OR payment$ OR support$)) |
| Search aspect 3  Outcome | TI=/AB=(Exercise$) OR TI=/AB=(Sport$) OR TI=/AB=(Recreation*) OR TI=/AB=(„Physical fitness“) OR TI=/AB=(„Physical conditioning“) OR TI=/AB=(„Weight loss“) OR TI=/AB=(„Physical exercise$“) OR TI=/AB=(„Sedentary behavio$r“) OR TI=/AB=(Activ*) OR TI=/AB=(Walk*) OR TI=/AB=(Fitness) OR TI=/AB=((exercise OR course) NEAR/3 (participation OR adherence OR attendance)) OR TI=/AB=(commut* NEAR/3 (active OR bicycl* OR bik* OR cycl* OR walk*)) |
| Search aspect 4  Study design | TI=/AB=(Random* NEAR/0 (alloc* OR assign*)) OR TI=/AB=(Controlled NEAR/0 (stud* OR trial*)) OR TI=/AB=(Blind NEAR/0 (single OR double OR treble OR triple)) OR TI=/AB=(Randomi*) OR TI=/AB=(Randomly) OR TI=/AB=(placebo) OR TI=/AB=(„Controlled clinical trial$“) OR TI=/AB=(Cluster) OR TI=/AB=(rct) OR TI=/AB=(crct) OR TI=/AB=(cct) |
| Search aspect 5  Exclusion | TI=(Child*) OR TI=(Teenage$) OR TI=(Parent$) OR TI=(Pregnant$) OR TI=(Juvenile$) OR TI=(Student$) |
| (Search aspect 1 AND search aspect 2 AND search aspect 3 AND search aspect 4) NOT search aspect 5 | |
| Search fields:  TI=/AB=() = TI (Title) OR AB (Abstract)  TI=() = Title | |
| Filter:  Language: english, german | |

Appendix 1. Tab. 7. Inclusion and exclusion criteria

| Components | Inclusion criteria | exclusion criteria |
| --- | --- | --- |
| Population | Employees, apprentices, trainees in high-income countries (Hamadeh et al., 2022)  All ages + genders  All socioeconomic backgrounds | Student collective  Patient collective |
| Intervention | Promotion of physical activity through financial incentives in the form of a cash value or voucher | Promotion of physical activity in the form of lottery or through commitment contracts. |
| Comparator | Active control groups (promotion of physical activity without financial incentives)  Passive control groups | No control group |
| Outcome | Physical activity measured by daily exercise time, step count, or participation in a sports class or use of a gym.  For studies that financially reward health-promoting behaviours (e.g., weight loss, healthy eating habits), a portion of the incentive must be used to promote physical activity. | - |
| Study design | Randomized controlled trials  Cluster randomized controlled trials  Non-randomized controlled trials | Observational studies  No peer review process |

Appendix 1. Tab. 8. Hierarchy for outcome selection

| **Ranking order** | **Criteria** |
| --- | --- |
| - 1. | The outcome describes the influence of financial incentives on physical activity. |
| - 2. | The outcome takes into account the criterion for which the financial incentives were given. |
| - 3. | Outcome that is measured objectively is preferable to that measured subjectively |
| - 4. | The outcome should - if possible - reflect the change of the whole group. |
| - 5. | The outcome should be comparable between groups. |
