# Supplementary material for: The impact of financial incentives on physical activity for employees in the context of workplace health promotion: a systematic review
Source: J Occup Health. 2024 Aug 19;66(1):uiae048. doi: 10.1093/joccuh/uiae048 (PMC11662443; doi:10.1093/joccuh/uiae048)
Supplement: Web_Material_uiae048 [file web_material_uiae048.zip › Appendix 2 Data extraction forms.docx]

Appendix 2. Data extraction forms (adapted from the Cochrane Consumers and Communication template (Ryan et al., 2016)).

1. Data extraction form – Carrera et al. (2020)

Appendix 2. Tab. 1.1 (Carrera et al. 2020)*.* *Section 1:* *General review information*

| **Item** | **Description** |
| --- | --- |
| Form version/date | Version 1.8, updated November 29, 2016 |
| Review Title | “The impact of financial incentives on physical activity for employees in the context of workplace health promotion - A systematic review” |
| Study ID | Carrera (2020) |
| Name of review author completing this form | Miriam Alice Vitzthum |
| Date form completed | 03/10/22 |
| Name of review author checking the data extracted to this form | Dr. Christopher Weyh |
| Author contact details for study | Prof. Mariana Carrera via e-mail  (mariana.carrera@montana.edu) on 26/08/22 + 09/09/22 |
| Further information required | Study protocol requested |
| Correspondence with authors successful or not; what information was received and when | Correspondence not successful |
| Will any additional unpublished data supplied by the authors be included in the review?  If so, note that the study will include unpublished data (for entry to RevMan) | - |
| Notes | Information source:  Carrera, M.; Royer, H.; Stehr, M. & Sydnor, J. (2020). The Structure of Health Incentives: Evidence from a Field Experiment. Management Science, 66 (5), p. 1890-1908. |

Appendix 2. Tab. 1.2 (Carrera et al. 2020)*. Section 2: Methods of the study*

| **Item** | **Description** | **Source** |
| --- | --- | --- |
| Aim of study | The aim of this study is to determine how financial incentives should be designed over time. | p. 1890 |
| Study design | This is a randomized controlled trial. (“Individuals were randomized into the treatments detailed in Table 1 (…). Both members and non-members were randomized into control, constant treatment, and kickstart treatment.”) | p. 1896 |
| Number of arms or group | There are 5 experimental arms, including 4 intervention groups and one control group  - Control group: no financial incentives  - Intervention group 1: continuous financial incentives (Constant): 10 USD/ visit for 8 weeks  - Intervention group 2: front-loaded financial incentives (Kickstart): 25 USD/ visit for the first 2 weeks, then 5 USD/ visit for the last 6 weeks  - Intervention group 3: continuous financial incentives in a shorter period (constant short): 10 USD/ visit for 4 weeks   - - Intervention group 4: constant periodic financial incentives (extended-sporadic): 10 USD/ visit for randomly selected 8 weeks in a 16-week period. | Tab. 1, p. 1896 |
| Consumer involvement | n. r. | - |
| Funding source | “This study was funded by the Robert Wood Johnson Foundation [Grant 69923] through the Applying Behavioral Economics to Perplexing Problems in Health and Healthcare initiative via the Center for Health Incentives and Behavioral Economics at the University of Pennsylvania. M. Stehr was supported by the LeBow College of Business Dean’s Research Fellowship.”  There is no information on whether the authors had any conflict of interest | p. 1890 |
| n. r. = not reported | | |

Appendix 2. Tab.1.3 (Carrera et al. 2020). Section 3: Risk of Bias assessment (modified according to Higgins et al. (2019a))

| **Domain** | **Risk of bias judgement** | **Source** |
| --- | --- | --- |
| bias arising from the randomization process | some  concerns | see Appendix 3: Assessment of risk of bias – Carrera et al. (2020) |
| bias due to deviations from the intended interventions (effect of assignment to intervention) | some  concerns |  |
| bias due to missing outcome data | high risk |  |
| bias in measurement of the outcome | low risk |  |
| bias in selection of the reported result | some  concerns |  |
| Overall bias | high risk |  |

Appendix 2**.** Tab. 1.4 (Carrera et al. 2020). Section 4: Study characteristics – Participants

| Item | | **Description** | **Source** |
| --- | --- | --- | --- |
| Description | | 1024 participants were randomly assigned to the experimental arms. 980 participants had usable data and were included in the final analyses. To avoid overloading the gym, 15 cohorts were formed. | pp. 1896 + 1897 |
| Geographic Location | | USA | p. 1895 |
| Setting | | Fortune 500 company in the Midwest | p. 1895 |
| Methods of recruitment of participants | | “We recruited subjects from a list of all employees at the firm’s headquarters by sending an e-mail that contained a link to an online survey on wellness. The e-mail informed participants that they could earn a $25 gift card for completing both this initial survey and a short follow up survey to be administered 8 weeks later.” | p. 1896 |
| Inclusion/Exclusion criteria for participation in study | | Only partial information available on inclusion and exclusion criteria (study protocol missing).  Exclusion criteria:   - unable to exercise for medical reasons - Workplace not at the location where the gym is located - Gym employees, managers, and human resources personnel who may have information about the study program. | p. 1907 |
| Age: range, mean (SD) | | All: non-members: 41.29 (11.43);  members: 40.72 (10.46);  range not reported | Tab. 2, p. 1898 |
| Gender | | Non-members: 48 % female;  members: 52 % female | s. a. |
| Ethnicity | | No information | s. a. |
| Other (social/demographic) details | education (college or more): | Non-members: 0.7; Members: 0.72 | s. a. |
|  | Physical activity (days of exercise last week) | Non-members: 2.24 (1.81); members: 3.57 (1.81) |  |
| Have important populations or groups been excluded from the study | | Before randomization, those who could not exercise for medical reasons and who did not work at the site where the gym was located were excluded ("The number of subjects decreased to 1,024 when we excluded those not medically able to exercise or who did not work at the site where the company gym is located.").  We also excluded gym staff, executives, and human-resource staff who might have been aware of the details of this program. | p. 1907 |

| **Nu****mbers involved** | | |
| --- | --- | --- |
| **Study numbers** | **Description** | **Source** |
| Eligible for inclusion | 1075 | p. 1897 + Appendix Fig. A1 |
| Excluded | 51 | s. a. |
| Refused to take part | - |  |
| Randomised to intervention group(s) | There were 980 participants assigned to the 5 groups.  IG 1: 280  IG 2: 284  IG 3: 138  IG 4: 106 | p. 1897, Appendix Fig. A1 |
| Randomised to control group | CG: 172 | Appendix Fig. A1 |
| Excluded post randomisation (for each group; with reasons if relevant) | After 3 cohorts had gone through the study, changes were made to the group assignment. This subsequently excluded 44 participants from the first 3 cohorts, resulting in a total number of 980 participants who were allocated to the groups. | p. 1908 |
| Withdrawn (for each group; with reasons if relevant) | - |  |
| Lost to follow up (for each group; with reasons) | Intervention groups (with reasons): Of the 980 participants, a total of 135 participants were lost by the time of the follow-up survey.  IG 1: 40  IG 2: 55  IG 3: 26  IG 4: 5  No reasons are given for the Lost to Follow-Up. | p. 1897, Appendix Fig. A1 |
|  |  | s. a. |
|  | Control group (with reasons):  CG: 9  No reasons are given for the Lost to Follow-Up. |  |
| Included in the analysis (for each group, for each outcome) | Primary Outcomes: Number of gym visits/week  IG 1: 280  IG 2: 284  IG 3: 138  IG 4: 106  CG: 172 | p. 1897 + Appendix Fig. A1 |
|  | Secondary Outcome: Effects on health (weight, high blood pressure, pulse).  Weight:  IG 1 – IG 4: 560  CG: 130  High blood pressure:  IG 1 – IG 4: 547  CG: 130  Pulse:  IG 1 – IG 4: 551  CG: 129 | s. a. |
| s. a. = see above; IG = Intervention Group; CG = Control Group | | |

Appendix 2. Tab. 1.5 (Carrera et al. 2020). Section 5: Study characteristics – Interventions (modified according to the current TIDieR checklist (Hoffmann et al., 2014)).

| **Item** | **Description** | **Source** |
| --- | --- | --- |
| Item 1. Brief name | Intervention group 1 (IG 1 - "constant"): constant financial incentives for gym visits  Intervention group 2 (IG 2 - "kickstart/front-load"): financial incentives for gym visits that are initially higher than later on  Intervention group 3 (IG 3 - "constant short"): constant financial incentives for gym visits for a shorter period of time  Intervention group 4 (IG 4 - "extended-sporadic"): financial incentives for gym visits over a longer period of time with random interruptions | Tab. 1, p. 1896 |
| Item 2. Why | By varying the amount and duration of the financial incentives, the aim is to find out which type of financial incentives work best. The background is that financial incentives can overcome the so-called 'present bias', which describes that people tend to value the immediate costs of physical activity higher than the future benefits. Financial incentives could be helpful in reducing the initial costs and driving habit formation. | p. 1891 |
| Item 3. What (Materials) | Nothing is reported about whether material was issued to participants. All information about the intervention seems to have been communicated by e-mail. No information is provided about how the voucher was delivered to participants. | p. 1896 |
| Item 4. What (Procedures) | IG 1: Participants are offered a financial incentive of USD 10 per gym visit. This can be used a maximum of once a day and twice a week. The financial value will be given to participants in the form of vouchers.  IG 2: Participants will be offered a financial incentive of 25 USD per gym visit for the first two weeks, and 5 USD per gym visit for the 6 weeks thereafter. These will be supported with financial incentives a maximum of once per day and a maximum of 2 times per week. The incentive period is 8 weeks. The financial value will be given to participants in the form of vouchers.  IG 3: The content of IG3 differs from IG1 only in the duration of the incentive period. Instead of over 8 weeks, IG3 is provided financial incentives over 4 weeks. Only non-members are assigned to this intervention group.  IG 4: Participants will receive 10 USD for each gym visit (maximum 1x/day and 2x/week). Like IG1, the incentive period lasts 8 weeks. However, the total duration of the program is 16 weeks. Participants will receive an e-mail informing them if the following week is a week in which they will receive financial incentives for gym visits.  The maximum incentive size is 160 USD (except IG 3 with 80 USD).  The control group did not receive any financial incentives or other types of motivation to increase physical activity.  Upon entering the gym, participants enter their company number into one of the two computer terminals located in the main entrance of the gym. This automatically generates the data needed for outcome measurement. There is no provision for logging out. | pp. 1895 + 1896 |
| Item 5. Who provided | No contact is reported between participants and study authors or other intervention providers during the intervention phase. | - |
| Item 6. How | Participants were free to decide on which days of the week they visited the gym (within opening hours: Monday to Friday from 6 a.m. to 6 p.m.) in order to receive the financial incentive. However, when the financial incentive passed to them is not described (possible after a certain number of weeks). | p. 1895 |
| Item 7. Where | The intervention was conducted in the gym at the company location where the participants work. The gym is open Monday through Friday from 6 a.m. to 6 p.m. and offers exercise classes, as well as a workout area with strengthening machines. Membership is subsidized and is 12.96 USD for two weeks. | s. a. |
| Item 8. When and how much | The intervention lasted 8 weeks. During this period, participants were offered a financial incentive of 10 USD per gym visit. They could take advantage of this a maximum of 1x/day and 2x/week. | p. 1891 |
| Item 9. Tailoring | It is not an individualized intervention, which is why there are no adaptation plans for the planned interventions. | - |
| Item 10. Modifications | No modifications of IG 1-IG 3 are reported.  IG4 was modified during the course of the intervention. After three cohorts had gone through the intervention, the study authors received feedback that those with the longer program duration of 16 weeks (as contrast to non-members the 8 weeks in IG1 and 2) did not want to enroll in a membership because the fees had to be paid for longer. Therefore, only those who already had a gym membership were assigned to this group, while the non-members were placed in IG3. This was done at the time after 3 out of 15 cohorts had already gone through the program. In retrospect, therefore, the non-members of the first three cohorts were excluded from IG4. | pp. 1907 + 1908 |
| Item 11. How well (planned) | One strategy to maintain treatment adherence was to offer a 25 USD voucher upon participation in the pre-study survey and the follow-up survey. | p. 1896 |
| Item 12. How well (actual) | 240 participants (approx. 86%) took part in the follow-up survey. | Appendix Fig. A1 |
| s. a. = see above | | |

Appendix 2. Tab.1.6 (Carrera et al. 2020). Section 6: Study characteristics – Outcomes and comparison groups

| **Primary Outcome** | **Method of assessing outcome measures** | **Method of follow up for non-respondents** | **Timing of outcome assessment**  ***(including frequency, length of follow up)*** |
| --- | --- | --- | --- |
| Gym visits (measured as "Any Visits" = at least 1 gym visit per week; binary variable, 0=no visit, 1=at least 1 visit/week). | Log-in data to the gym | ITT analysis | Outcome data were generated each time an individual logged into the gym.  Gym visits/week were assessed during the incentive period (weeks 1-8) and the post-incentive period (weeks 9-16). |
| Source: pp. 1895, 1897 + 1900 | | | |

Appendix 2. Tab.1.7 (Carrera et al. 2020). Section 7: Data and results

| **Pri-mary Out-come** | **Timing of outcome assess-ment** | **IG1**  **Proportion of participants with at least a weekly gym attendance (SE)** | **IG2**  **Proportion of participants with at least a weekly gym attendance (SE)** | **IG3**  **Proportion of participants with at least a weekly gym attendance (SE)** | **IG4**  **Proportion of participants with at least a weekly gym attendance (SE)** |
| --- | --- | --- | --- | --- | --- |
| Gym visits for non-mem-bers | Week 1-4 | 0.116*** (0.027) | 0.113*** (0.027) | 0.064** (0.026) | - |
|  | Week 5-8 | 0.106*** (0.025) | 0.074*** (0.023) | 0.027 (0.020) | - |
|  | Week 9-16 | 0.036** (0.018) | 0.030 (0.018) | 0.017 (0.018) | - |
| Gym visits for  Mem-bers | Week 1-8 | 0.184*** (0.048) | 0.119*** (0.044) | - | 0.111** (0.044) |
|  | Week 9-16 | 0.093*** (0.047) | 0.009 (0.043) | - | 0.137*** (0.046) |
| Statistical calculation using regression analysis; *p < 0.1; **p < 0.05; ***p < 0.01.  Source: Tab. 4 (p. 1901) + Tab. 5 (p. 1903) | | | | | |

1. Data extraction form – Finkelstein et al. (2016)

Appendix 2. Tab. 2.1 (Finkelstein et al. 2016). Section 1: General review information

| **Item** | **Description** |
| --- | --- |
| Form version/date | Version 1.8, updated November 29, 2016 |
| Review Title | “The impact of financial incentives on physical activity for employees in the context of workplace health promotion - A systematic review” |
| Study ID | Finkelstein (2016) |
| Name of review author completing this form | Miriam Alice Vitzthum |
| Date form completed | 04/10/22 |
| Name of review author checking the data extracted to this form | Dr. Christopher Weyh |
| Author contact details for study | Dr. Robert Sloan via ResearchGate on 01/09/22 |
| Further information required | Study protocol requested |
| Correspondence with authors successful or not; what information was received and when | Correspondence successful: Dr. Sloan provided study protocol and additional results tables (wear time-adjusted results) (08/09/22). |
| Will any additional unpublished data supplied by the authors be included in the review?  If so, note that the study will include unpublished data (for entry to RevMan) | - |
| Notes | Information sources:  Finkelstein, E. A.; Haaland, B. A.; Bilger, M.; Sahasranaman, A.; Sloan, R. A.; Khaing Nang, E. E. & Evenson, K. R. (2016). Effectiveness of activity trackers with and without incentives to increase physical activity (TRIPPA): a randomised controlled trial. Lancet Diabetes & Endocrinology, 4 (12), p. 1-13.  Finkelstein, E. A.; Sahasranaman, A.; John, G.; Haaland, B. A.; Bilger, M.; Sloan, R. A.; Khaing Nang, E. E. & Evenson, K. R. (2915). Design and baseline characteristics of participants in the TRial of Economic Incentives to Promote Physical Activity (TRIPPA): A randomized controlled trial of a six month pedometer program with financial incentives  Appendix: Wear time-adjusted results tables  Further source of information (same study):  Sloan, R. A.; Kim, Y.; Sahasranaman, A.; Müller-Riemenschneider, F.; Biddle, S. J. H. & Finkelstein, E. A. (2018). The influence of a consumer‑wearable activity tracker on sedentary time and prolonged sedentary bouts: secondary analysis of a randomized controlled trial. BMC Res Notes, 11 (1), 189. |

Appendix 2. Tab. 2.2 (Finkelstein et al. 2016). Section 2: Methods of the study

| **Item** | **Description** | **Source** |
| --- | --- | --- |
| Aim of study | The aim of the study is to investigate whether activity trackers alone or in combination with financial incentives or charitable donations lead to an increase in physical activity and improvements in health outcomes.. | p. 1 |
| Study design | This was a randomized controlled trial.  However, individuals in existing groups of 4 were also assigned to an experimental group (“Although randomisation was done at the individual level, we allowed exercise companions to sign up in groups of up to four members and be randomly allocated to the same study group.”).  In fact, however, there were not many: “Even though participants were allowed to enrol in groups of up to four people, 771 (96 %) participants enrolled as singletons.” | p. 1  p. 3  p. 7 |
| Number of arms or group | There are 4 experimental arms, including 3 intervention groups and one control group.  CG: no pedometers and no financial incentives.  IG 1: FitBit activity tracker  IG 2: FitBit activity tracker + charitable donations  IG 3: FitBit activity tracker + financial incentives.  The financial incentive, or charity donation for IG 2 and IG 3 are 15 SGD for 50,000 to 70,000 steps/week, and 30 SGD for >70,000 steps/week. | pp. 1 + 4 |
| Consumer involvement | n. r. | - |
| Funding source | The study was funded by the Singapore Ministry of Health. The study authors declare that the funder had no influence on the study design, data collection and interpretation, or the writing of the report.  The authors declare no conflict of interests ("Declaration of interests: We declare no competing interests."). | pp. 1, 2 + 7 |
| n. r. = not reported; IG = Intervention Group; CG = Control Group | | |

Appendix 2. Tab. 2.3 (Finkelstein et al. 2016). Section 3: Risk of Bias assessment (modified according to Higgins et al. (2019a))

| **Domain** | **Risk of bias judgement** | **Source** |
| --- | --- | --- |
| bias arising from the randomization process | low risk | see Appendix 3: Assessment of risk of bias – Finkelstein et al. (2016) |
| bias due to deviations from the intended interventions (effect of assignment to intervention) | low risk |  |
| bias due to missing outcome data | low risk |  |
| bias in measurement of the outcome | low risk |  |
| bias in selection of the reported result | low risk |  |
| Overall bias | low risk |  |

Appendix 2. Tab. 2.4. Section 4: Study characteristics – Participants

| **Item** | | **Description** | | | | | | **Source** |
| --- | --- | --- | --- | --- | --- | --- | --- | --- |
| Description | | The study involved 800 full-time employees. Most of them had a desk job, which offered little opportunity for workplace-related physical activity. | | | | | | p. 3 |
| Geographic Location | | Singapore | | | | | | s. a. |
| Setting | | „(…) done in 13 organisations spanning many industries and sectors of government (…)”  „(…) we recruited 800 full-time employees from 15 worksite.“ | | | | | | s. a.  p. 7 |
| Methods of recruitment of participants | | Companies were approached through existing contacts and "cold calls." If companies responded positively, a study briefing was conducted to inform management of the details of the study. After management confirmed participation, recruitment materials (e.g., electronic direct mail, posters, and newsletters) containing organization-specific information about the study were distributed to employees through internal channels. | | | | | | s. a. |
| Inclusion/Exclusion criteria for participation in study | | Inclusion Criteria:   - English-speaking - employed full time - Aged between 21-65 years - Willingness to be randomly assigned to one of the four study groups - Willing to wear an activity tracker for the duration of the study - Be able to walk at least 10 steps continuously   Exclusion criteria:   - Pregnancy | | | | | | s. a. |
| Age: range, mean (SD) | | IG 1: 35.4 (8.3)  IG 2: 35.5 (8.6)  IG 3: 35.5 (8.4)  CG: 35.6 (8.6) | | | | | | Tab. 1, p. 6 |
| Gender | | IG 1: 49 % female  IG 2: 53 % female  IG 3: 57 % female  CG: 56 % female | | | | | | s. a. |
| Ethnicity | | Chinese  IG 1:  66 %  IG 2:  73 %  IG 3:  67 %  CG: 69 % | Malay  IG 1:  6 %  IG 2:  3 %  IG 3:  4 %  CG:  5 % | Indian  IG 1:  13 %  IG 2:  9 %  IG 3:  16 %  CG:  15 % | Other  IG 1:  13 %  IG 2:  14 %  IG 3:  12 %  CG:  9 % | Not specified  IG 1: 2 %  IG 2: 3 %  IG 3: 1 %  CG: 2 % | | s. a. |
| Other (social/demographic) details | Education | High school or lower  IG 1: 4 %  IG 2: 4 %  IG 3: 6 %  CG: 6 % | Some college  IG 1: 13 %  IG 2: 11 %  IG 3: 14 %  CG:  15 % | College graduate  IG 1:  49 %  IG 2:  50 %  IG 3:  41 %  CG:  49 % | Postgraduate  IG 1:  29 %  IG 2:  30 %  IG 3:  37 %  CG:  28 % | Other  IG 1:  1 %  IG 2:  3 %  IG 3:  2 %  CG:  0 % | Not declared  IG 1:  3 %  IG 2:  3 %  IG 3:  1 %  CG:  2 % | s. a. |
|  | Physical activity | MVPA min per week  IG 1: 131 (114-148)  IG 2: 114 (100-127)  IG 3: 131 (115-148)  CG: 121 (105-137) | | | Daily step count  IG 1: 8000 (7670-8330)  IG 2: 7780 (7460-8090)  IG 3: 8300 (7950-8650)  CG: 8030 (7680-8380) | | |  |
| Have important populations or groups been excluded from the study | | - | | | | | | - |

| **Numbers involved** | | | |
| --- | --- | --- | --- |
| **Study numbers** | **Description** | | **Source** |
| Eligible for inclusion | 1307 participants assessed for eligibility | | Fig. 1, p. 5 |
| Excluded | 507 excluded | | s. a. |
| Refused to take part | - | | - |
| Randomised to intervention group(s) | There were 800 participants assigned to the 4 groups.  IG 1: 203  IG 2: 199  IG 3: 197 | | s. a. |
| Randomised to control group | CG: 201 | | s. a. |
| Excluded post randomisation (for each group; with reasons if relevant) | IG 1: 1 (Participation in a separate weight loss study)  IG 2: 1 (pregnancy)  IG 3: 0  CG: 0 | | s. a. |
| Withdrawn (for each group; with reasons if relevant) | IG 1: 0  IG 2: 4 (declined participation after group assignment)  IG 3: 0  CG: 0 | | s. a. |
| Lost to follow up (for each group; with reasons) | Intervention groups (with reasons) | | s. a. |
|  | After 6 months  IG 1: 17 (14 canceled or cannot be contacted, 2 became pregnant, 1 non-study related injury)  IG 2: 21 (14 canceled or cannot be contacted, 2 became pregnant, 3 emigrated, 1 not available, 1 malfunction of the pedometer)  IG 3: 7 (4 canceled or cannot be contacted, 1 became pregnant, 1 emigrated, 1 malfunction of the pedometer) | After 12 months  IG 1: 26 (21 canceled or cannot be contacted, 3 became pregnant, 1 emigrated, 1 retired)  IG 2: 31 (25 canceled or cannot be contacted, 3 became pregnant, 1 emigrated, 1 not available, 1 malfunction of the pedometer)  IG 3: 22 (17 canceled or cannot be contacted, 2 became pregnant, 1 emigrated, 2 not available) |  |
|  | Control group (with reasons) | |  |
|  | After 6 months  CG: 12 (8 canceled or cannot be contacted, 2 became pregnant, 1 emigrated, 1 malfunction of the pedometer) | After 12 months  CG: 24 (22 canceled or cannot be contacted, 1 emigrated, 1 malfunction of the pedometer) |  |
|  | Relationship of Lost to follow-up and sociodemographic predictors: gender (females 1.8 times more likely to drop out than males) and ethnicity (participants of Indian origin were 3.2 times more likely and participants of other origins were 1.8 times more likely to drop out than participants of Chinese origin) | | p. 7 |
| Included in the analysis (for each group, for each outcome) | Primary outcome: physical activity measured in MVPA min/week.  Secondary Outcomes:  Physical activity measured in daily steps  Physical activity measured in participants who reached the target (more than 70,000 steps/week)  Changes in health outcomes (weight, systolic blood pressure, NET-FVO2max, change in EQ-5D-SL index)  Included in intention-to-treat analysis:  IG 1: 203  IG 2: 199  IG 3: 197  CG: 201 | | Fig. 1, p. 5 |
| s. a. = see above | | | |

Appendix 2. Tab. 2.5 (Finkelstein et al. 2016). Section 5: Study characteristics – Interventions (modified according to the current TIDieR checklist (Hoffmann et al., 2014))

| **Item** | **Description** | **Source** |
| --- | --- | --- |
| Item 1. Brief name | IG 1: Activity tracker and website (Fitbit).  IG 2: Activity tracker and financial incentive that can be donated to charity.  IG 3: Activity tracker and field incentive. | pp. 1 + 4 |
| Item 2. Why | There is insufficient data on the effectiveness of pedometer-based interventions in the workplace. The problem is that the feedback on daily steps taken wears off quickly and the activity trackers are no longer worn, so there can be no positive impact on physical activity. There are various strategies to counteract this, such as the possibility of connecting the activity trackers to social networks to increase their value again. Another strategy is to link the activity trackers with financial incentives to achieve activity goals.  Regarding the strategy of promoting physical activity through financial incentives donated to charity, there is no research to date. Nevertheless, it could be a promising approach because no money is paid for exercise and because it is consistent with the theory of reasoned action, which states that people perform a behavior when they value it positively and believe it is important that others should perform it. | pp. 2 + 3 |
| Item 3. What (Materials) | All participants: “(…) all participants received educational booklets, published by the Singapore Health Promotion Board, entitled Active for Life and Brisk Walk Your Way to Better Health. These booklets give readers a better understanding of the benefits of and strategies for increasing physical activity.”  In addition, all participants received an accelerometer (“All physical activity outcomes, including steps, were measured for participants in all groups via sealed accelerometers (ActiGraph triaxial GT-3x+ accelerometer [ActiGraph, Pensacola, FL, USA]). We instructed participants to wear the accelerometer for at least 10 waking hours on each day for 7 consecutive days during each assessment interval”  Participants of IG1-IG3: “(…) received the Fitbit Zip wireless activity tracker (Fitbit, San Francisco, CA) and access to the Fitbit website, which provides feedback on daily step activity and additional features (eg, badges, competitions) aimed to sustain increased activity levels.” | pp. 3 + 4  s. a. |
| Item 4. What (Procedures) | Duration: 6 months intervention + 6 months follow-up.  Online questionnaire at the beginning of the intervention: “participants were also requested to complete an online baseline questionnaire on basic socio-demographic information and additional self-reported secondary outcomes data.”  Wearing the activity tracker: “We instructed participants to wear the Fitbit in the same position every day.”  “We instructed participants to wear the accelerometer for at least 10 waking hours on each day for 7 consecutive days during each assessment interval.”  “Detailed instructions on accelerometer wear were provided to all participants verbally via a demonstration, and through instruction sheets.”  “(…) and an accelerometer was issued to collect baseline physical activity data for a one-week period.”  IG 2 + IG 3: “they could earn weekly incentives: S$15 if they logged between 50 000 and 70 000 steps per week or S$30 if they logged 70 000 or more steps per week.”  Difference between IG 2 + IG 3: “The key difference between the incentive groups was that the cash group participants received the incentives for themselves, whereas the charity group participants raised money for a charity of their choice from a list of 13 local charities representing (…). Participants in the cash and charity groups also had access to the TRIPPA website where they could track incentives earned.”  IG 1 + CG: “To keep the other participants engaged in the study, control and Fitbit participants earned a weekly participation payment of S$4, irrespective of number of steps recorded.”  All experimental groups: “We distributed these payments, and the incentive payments, at the participants’ worksites every 4–6 weeks. Donations to charities were made via an online portal. Before receiving incentive payments, we asked participants in the charity and cash groups to sign an oath affirming that they (or their designated charity) were being paid for their own physical activity. Research reveals that such oaths reduce the likelihood of participants engaging in dishonest behaviour.”  “All physical activity outcomes, including steps, were measured for participants in all groups via sealed accelerometers (ActiGraph triaxial GT-3x+ accelerometer [ActiGraph, Pensacola, FL, USA]) at baseline, 6 months, and 12 months.”  Procedure for activity measurement: “We quantified mean counts per min measured by the accelerometer by dividing the sum of total counts by the number of min per day of wear time across all adherent days (defined as ≥10 h/day of wear).” | Study protocol, p. 240  p. 4  s.a.  Study protocol  p. 240  s. a.  p. 4  s. a.  s. a.  s. a.  s. a.  s. a. |
| Item 5. Who provided | No contact between participants and the study authors or other intervention providers is necessary for the implementation of the intervention. No such contact is reported. |  |
| Item 6. How | The intervention was offered as an individual intervention (individual participants were not dependent on others participating in their group).  There are no reports of contact being made with participants during the intervention. | s. a. |
| Item 7. Where | The intervention did not require a location other than where participants worked and spent their daily lives (steps were recorded not only during work hours, but throughout the day). “We instructed participants to wear the accelerometer for at least 10 waking hours on each day for 7 consecutive days during each assessment interval.” | p. 4 |
| Item 8. When and how much | The intervention period was 6 months with a subsequent follow-up period of another 6 months.  Activity tracker for IG 1-3: "We instructed participants to wear the accelerometer for at least 10 waking hours on each day for 7 consecutive days during each assessment interval.”  “We distributed these payments, and the incentive payments, at the participants’ worksites every 4-6 weeks.”  Financial incentives for IG 2 + IG 3: “they could earn weekly incentives: S$15 if they logged between 50 000 and 70 000 steps per week or S$30 if they logged 70 000 or more steps per week.”. | p. 3  p. 4  s. a.  s. a. |
| Item 9. Tailoring | It is not an individualized intervention, which is why there are no adaptation plans for the planned interventions. | - |
| Item 10. Modifications | Due to anomalies regarding the wearing time of IG 3 compared to the other groups, results were presented in which the wearing time was taken into account. This was done because the study authors could not separate the competing hypotheses of increased wearing time or physical activity or both. | p. 7 |
| Item 11. How well (planned) | A strategy to maintain treatment adherence: “To encourage Participants to attend follow-up assessments, we gave participants in all four groups a S$25 supermarket voucher and a one in ten chance of receiving a S$50 voucher for completing the month 6 assessment, and a S$35 voucher and one in ten chance of receiving a S$50 voucher for completing the month 12 assessment.”  No other strategies to maintain treatment adherence or its assessment are reported. | p. 4 |
| Item 12. How well (actual) | After 6 months: “(…) 57 (7 %) participants were lost to follow up.”  After 12 months: “(…) 153 (19 %) participants were lost to follow-up.” | p. 7 |
| s. a. = see above | | |

Appendix 2. Tab. 2.6 (Finkelstein et al. 2016). Section 6: Study characteristics – Outcomes and comparison groups

| **Secondary Outcome** | | **Method of assessing outcome measures** | **Method of follow up for non-respondents** | **Timing of outcome assessment (including frequency, length of follow up)** |
| --- | --- | --- | --- | --- |
| Physical activity (measured in average number of daily steps) | | Accelerometer | ITT analysis | Data for outcome were generated each time the activity tracker recorded a movement.  At the post-intervention time point (after 6 months) and after the follow-up period (after 12 months), the outcome was assessed. |
| Effects on health | Weight | Objective measurement using scales | ITT analysis | The outcome was evaluated at the time after the intervention (after 6 months) and after the follow-up period (after 12 months). |
|  | Systolic blood pressure | Objective measurement using a blood pressure monitor | ITT analysis | s. a. |
|  | Cardiorespiratory fitness (maximum oxygen uptake) | VO2max non-exercise test of cardiorespiratory fitness [NET-F] | ITT analysis | s. a. |
|  | Quality of life | EuroQoL (EQ-5D-5L) questionnaire | ITT analysis | s. a. |
| Source: pp. 4 + 5  s. a. = see above | | | | |
| Adverse events: “Because of the low risks involved, the institutional review board did not require collection and reporting of adverse events. (p. 4) | | | | |

Appendix 2. Tab. 2.7 (Finkelstein et al. 2016). Section 7: Data and results

| **Secon-dary Out-come** | **Timing of outcome assess-ment** | **IG 1 (Fitbit)** | | | **IG 3 (Cash)** | | | | **CG** | |
| --- | --- | --- | --- | --- | --- | --- | --- | --- | --- | --- |
|  |  | **Diffe-rence from baseline** | **Diffe-rence to CG** | | **Difference from baseline** | | **Diffe-rence to CG** | | **Difference from baseline** | |
| Physical activity (mea-sured in average number of daily steps) | After the incentive period (6 months) | -130 [-490; 230]; p=0.4495 | 340 [-100; 790];  p=0.1362 | | 570 [210; 930]; p=0.0016* | | 1050 [600; 1490];  p<0.0001* | | -480 [-830; -120]; p=0.0099* | |
|  | After the follow-up period (12 months) | -30 [-420; 370]; p=0.8950 | 450 [-10; 910]; p=0.0561 | | 30 [-370;  430];  p=0.8853 | | 500 [50; 960];  p=0.0289* | | -480 [-880; -80]; p=0.0198* | |
| Data are changes in accelerometer-derived endpoints [95% CI]; p-value. * p<0,05.  Source: Tab. 2 (p. 9) + Tab. 3 (p. 10) | | | | | | | | | | |
| **Secon-dary Outc-omes: Effects on health** | **Timing of outcome assess-ment** | **IG 1 (Fitbit)** | | | | **IG 3 (Cash)** | | | | **CG** |
|  |  | **Difference from baseline** | | **Diffe-rence to CG** | | **Difference from baseline** | | **Diffe-rence to CG** | | **Diffe-rence from baseline** |
| Weight | After the incentive period (6 months) | 0.2 [-0.6; 0.9];  p=0.7959 | | 0.49 [-0.54; 1.53];  p=0.4185 | | 0.4 [-0.3;  1.1];  p=0.3122 | | 0.71 [-0.31; 1.73];  p=0.1805 | | -0.3 [-1.0; 0.4];  p=0.3725 |
|  | After the follow-up period (12 months) | -0.4 [-2.1; 1.2];  p=0.5882 | | 0.9 [-1.2; 3.0]; p=0.4188 | | -0.6 [-2.1; 1.0];  p=0.4575 | | 0.7 [-1.2; 2.7]; p=0.4665 | | -1.3 [-2.8; 0.2];  p=0.0957 |
| Systolic blood pressure | After the incentive period (6 months) | -1.3 [-4.7; 2.1];  p=0.4838 | | -3.80 [-8.09;  0.49];  p=0.0918 | | -0.3 [-3.7; 3.0];  p=0.7488 | | -2.85 [-7.05; 1.35];  p=0.1684 | | 2.5 [-0.8; 5.9];  p=0.1620 |
|  | After the follow-up period (12 months) | -0.2 [-4.1; 3.7];  p=0.9319 | | 0.1 [-4.9; 5.1]; p=0.9666 | | -0.9 [-4.6; 2.8];  p=0.6372 | | -0.6 [-5.5; 4.3]; p=0.8057 | | -0.3 [-3.9; 3.3];  p=0.8798 |
| Cardiorespiratory fitness (max. oxygen uptake) | After the incentive period (6 months) | 0.7 [0.3;  1.1];  p=0.0002 | | 0.35 [-0.21; 0.92];  p=0.1452 | | 0.9 [0.5;  1.3];  p<0.0001 | | 0.51 [-0.06; 1.08];  p=0.0778 | | 0.4 [-0.1; 0.8];  p=0.0809 |
|  | After the follow-up period (12 months) | 1.6 [0.6;  2.6]; p=0.0017 | | 0.2 [-0.8; 1.2]; p=0.7453 | | 1.7 [0.7;  2.6];  p=0.0008 | | 0.2 [-0.8; 1.2]; p=0.6578 | | 1.4 [0.5;  2.4];  p=0.0031 |
| Quality of life | After the incentive period (6 months) | -1.8 [-3.8 0.2];  p=0.0863 | | 0.26 [-2.45;  2.96];  p=0.8517 | | -0.5 [-2.5; 1.4];  p=0.5731 | | 1.52 [-1.14;  4.17];  p=0.2861 | | -2.1 [-4.0 -0.1];  p=0.0515 |
|  | After the follow-up period (12 months) | -1.3 [-3.3; 0.6];  p=0.1848 | | 0.4 [-2.2; 3.0]; p=0.7614 | | -0.1 [-2.0; 1.8];  p=0.9405 | | 1.6 [-1.0; 4.3]; p=0.2237 | | -1.7 [-3.7; 0.3];  p=0.0898 |
| Data are changes in health outcomes [95% CI]; p-value.  Source: Tab. 4 (p. 11) | | | | | | | | | | |

1. Data extraction form – Hunter et al. (2013)

Appendix 2. Tab. 3.1 (Hunter et al. 2013). Section 1: General review information

| **Item** | **Description** |
| --- | --- |
| Form version/date | Version 1.8, updated November 29, 2016 |
| Review Title | “The impact of financial incentives on physical activity for employees in the context of workplace health promotion - A systematic review” |
| Study ID | Hunter (2013) |
| Name of review author completing this form | Miriam Alice Vitzthum |
| Date form completed | 04/10/22 |
| Name of review author checking the data extracted to this form | Dr. Christopher Weyh |
| Author contact details for study | Ruth F. Hunter via e-mail (ruth.hunter@qub.ac.uk) on 29/09/22 |
| Further information required | Study protocol requested |
| Correspondence with authors successful or not; what information was received and when | Correspondence successful.  Prof. F. Hunter writes on 29/09/22: „(…) there is no study protocol published for the AJPM published paper.” |
| Will any additional unpublished data supplied by the authors be included in the review?  If so, note that the study will include unpublished data (for entry to RevMan) | - |
| Notes | Information source:  Hunter, R. F., Tully, M. A., Davis, M., Stevenson, M. & Kee, F. (2013) Physical Activity Loyalty Cards for Behavior Change. A Quasi-Experimental Study, *Am J Prev Med, 45* (1), p. 56-63.  Further source of information (same study):  Dallat, M.A.T., Hunter, R.F., Tully, M.A.; Cairns, K.J & Kee, F. (2013). A lesson in business: cost-effectiveness analysis of a novel financial incentive intervention for increasing physical activity in the workplace. *BMC Public Health 13*, 953. |

Appendix 2. Tab. 3.2 (Hunter et al. 2013). Section 2: Methods of the study

| **Item** | **Description** | **Source** |
| --- | --- | --- |
| Aim of study | The purpose of the study is to examine the effectiveness of financial incentives to increase physical activity in the workplace. | p. 56 |
| Study design | This is a quasi-experimental study: the assignment of the participants to the experimental and control groups is not random, but depends on which of the two buildings they work in. Building A is the experimental group, Building B the control group. („All eligible participants in Building A were assigned to the Incentive Group, and those in Building B were assigned to the No Incentive Group..“) | p. 57 |
| Number of arms or group | There are two study arms, including an intervention group and a control group  IG: Participation in the PAL intervention with financial incentives.  CG: Participation in the PAL intervention without financial incentives. | p.56 |
| Consumer involvement | n. r. | - |
| Funding source | “This research was supported by funding from the National Prevention Research Initiative (NPRI) (grant number G0802045) and their funding partners.”  “No financial disclosures were reported by the authors of this paper..” | p. 62 |
| n. r. = not reported | | |

Appendix 2. Tab. 3.3 (Hunter et al. 2013). Section 3: Risk of Bias assessment (modified according to Sterne et al. (2016a))

| **Domain** | **Risk of bias judgement** | **Source** |
| --- | --- | --- |
| Bias due to confounding | low risk | see Appendix 3: Assessment of risk of bias – Hunter et al. (2013) |
| Bias in selection of participants into the study | low risk |  |
| Bias in classification of interventions | moderate risk |  |
| Bias due to deviations from intended interventions | low risk |  |
| Bias due to missing data | no information |  |
| Bias in measurement of outcomes | moderate risk |  |
| Bias in selection of the reported result | low risk |  |
| Overall Bias | moderate risk |  |

Appendix 2. Tab. 3.4 (Hunter et al. 2013). Section 4: Study characteristics – Participants

| **Item** | | **Description** | **Source** |
| --- | --- | --- | --- |
| Description | | Employees with sedentary jobs and high sick leave. | p. 57 |
| Geographic Location | | Northern Ireland | s. a. |
| Setting | | Two large buildings of the main government agencies in Belfast, set in over 300 acres of parkland. | p. 56 |
| Methods of recruitment of participants | | Employees were recruited via e-mail, posters and web links from the intranet sites. Interested individuals were directed to the study website where they could access more information, register to participate, and complete a screening questionnaire. Eligible participants then gave informed consent, received a PAL card, and were asked to complete an exit questionnaire. | p. 57 |
| Inclusion/Exclusion criteria for participation in study | | Inclusion criteria:   - ≥4 days/week and ≥6 hours/day working in an office setting. - Be between the ages of 16-65 years - Be able to walk moderately fast for 15 minutes (self-reported)   Exclusion criteria:   - Express advice from a primary care physician not to exercise. | s. a. |
| Age: range, mean (SD) | | All: 43.32 (9.37)  IG: 43.30 (9.58)  CG: 43.34 (9.20)  Range is not reported. | p. 59, Tab. 2 (p. 59) |
| Gender | | All: 67 % female  IG: 66 % female  CG: 68 % female | s. a. |
| BMI | | IG: 27.16 (26.34, 28.02)  CG: 26.92 (26.28, 27.54) | Tab. 2 (p. 59) |
| Other (social/demographic) details | Education *(University degree or higher)* | IG: 40.2 %  CG: 38.2 % | s. a. |
|  | Physical activity *(GPAQ: General physical activity*  *questionnaire;)* | IG: 20.1 % high, 23.1 % moderate, 56.8 % low  CG: 20.8 % high, 30.4 % moderate, 48.8 % low |  |
| Have important populations or groups been excluded from the study | | n. r. | - |

| **Numbers involved** | | |
| --- | --- | --- |
| **Study numbers** | **Description** | **Source** |
| Eligible for inclusion | n. r. | - |
| Excluded | n. r. | - |
| Refused to take part | n. r. | - |
| Randomised to intervention group(s) | There were 406 participants assigned to the two groups.  IG: 199 | p. 59 |
| Randomised to control group | CG: 207 | s. a. |
| Excluded post randomisation (for each group; with reasons if relevant) | n. r. | - |
| Withdrawn (for each group; with reasons if relevant | n. r. | - |
| Lost to follow up (for each group; with reasons) | IG: 31 (no reasons reported)  CG: 32 (no reasons reported) | Tab. 3 (p. 60) |
|  | Relationship of Lost to Follow-Up and sociodemographic predictors: n. r. | - |
| Included in the analysis (for each group, for each outcome) | Primary and secondary outcomes:  IG: 199  CG: 207 | Tab. 3 (p. 60) |
| n. r. = not reported, s. a. = see above | | |

Appendix 2. Tab. 3.5 (Hunter et al. 2013). Section 5: Study characteristics – Interventions (modified according to the current TIDieR checklist (Hoffmann et al., 2014))

| **Item** | **Description** | **Source** |
| --- | --- | --- |
| Item 1. Brief name | Intervention Group (IG): PAL intervention + financial incentive | p. 58 |
| Item 2. Why | The pandemic of inactivity is causing many health challenges in the population. New approaches are being sought to meet physical activity recommendations. One of these approaches is that of the supportive environment, such as the workplace, because higher levels of physical activity in the workplace could have physical and mental benefits for employees, as well as lead to economic benefits through reduced absenteeism and increased productivity on the part of employers. There are some studies that have investigated the positive effects of financial incentives on physical activity. In terms of incentive design, the authors draw inspiration from the successful approach of retailers who use loyalty and reward cards to motivate consumers to make more purchases. How successfully this can be applied to influencing health behavior is investigated in this study using Physical Activity Loyalty (PAL) cards. | pp. 56 + 57 |
| Item 3. What (Materials) | All participants were given "Introduction pack" which included PAL card and information on how to use it. | p. 57 |
| Item 4. What (Procedures) | Duration: Primary outcome: 12 weeks intervention; Secondary outcomes: 12 weeks intervention + 6 months follow-up.  When participants wanted to engage in physical activity during their workday, they scanned their PAL card at near-field communication (NFC) sensors placed along the sidewalks and at the entrance to the workplace gym. Each time participants swiped their card, a timestamp was created that recorded the date and time of each swipe. The minutes between each timestamp were then added together to determine the total minutes of physical activity for each workout completed. Participants in both groups were able to receive real-time feedback of their physical activity, allowing them to self-monitor their activity at work by logging into their personal account on the study website.  IG: In the incentive group, unlike the CG, minutes of physical activity were also converted into points (1 minute = 1 point; upper limit: 30 points per day, equivalent to one pound), and these points could be redeemed for rewards (shopping vouchers) at weeks 6 and 12.  The PAL card did not record activities undertaken outside the workplace premises. | pp. 57 + 58, Dallat (2013), p. 3 |
| Item 5. Who provided | No contact between the participants and the study authors or other intervention providers is necessary for the implementation of the intervention. No such contact is reported. | - |
| Item 6. How | The intervention was offered as an individual intervention (individual participants were not dependent on others participating in their group). | - |
| Item 7. Where | The intervention was conducted on designated walking paths in the workplace area, where sensors were placed to monitor activity. | p. 57 |
| Item 8. When and how much | The intervention period was 12 weeks with a subsequent follow-up period of 6 months.  The IG participants were able to redeem the points they had collected for rewards (shopping vouchers) in weeks 6 and 12. | pp. 58 + 59 |
| Item 9. Tailoring | It is not an individualized intervention, which is why there are no adaptation plans for the planned interventions. | - |
| Item 10. Modifications | No modifications of the intervention during the study are reported. | - |
| Item 11. How well (planned) | No strategies for maintaining adherence or assessing it are reported. | - |
| Item 12. How well (actual) | - | - |

Appendix 2. Tab. 3.6 (Hunter et al. 2013). Section 6: Study characteristics – Outcomes and comparison groups

| **Primary Outcome** | **Method of assessing outcome measures** | **Method of follow up for non-respondents** | **Timing of outcome assessment**  ***(including frequency, length of follow up)*** |
| --- | --- | --- | --- |
| Physical activity | Objective measurement by using PA tracking system | ITT analysis | Data for outcome were recorded continuously during the 12-week intervention, each time participants swiped their PAL card at a sensor.  Differences between the IG and the CG were compared at baseline and at weeks 6 and 12. |
| Source: pp. 58 + 59 | | | |
| Adverse events: not reported | | | |
| **Secondary Outcome** | **Method of assessing outcome measures** | **Method of follow up for non-respondents** | **Timing of outcome assessment**  ***(including frequency, length of follow up)*** |
| Health (mental and physical component) | SF-8 (www.sf-36.org/tools/  sf8.shtml) | ITT analysis | Data for these outcomes were included at baseline, after the 12-week intervention, and after 6 months of follow-up. |
| Source: p. 58 | | | |

Appendix 2. Tab. 3.7 (Hunter et al. 2013). Section 7: Data and results

| **Primary Outcome** | | **Timing of outcome assessment (days/months)** | **IG** | **CG** | **p-value of the difference IG vs. CG** |
| --- | --- | --- | --- | --- | --- |
| Physical activity (in min/week [95% CI]) | | Baseline | 44.79 [36.95; 52.62] | 49.06 [41.42; 56.70] | 0.44 |
|  |  | After 6 weeks | 26.18 [20.06; 32.29] | 24.00 [17.45; 30.54] | 0.45 |
|  |  | After 12 weeks | 17.52 [12.49; 22.56] | 16.63 [11.76; 21.51] | 0.59 |
| Source: Tab. 2 (p. 59) + Tab. 3 (p. 60) | | | | | |
| **Secondary Outcome** | | **Timing of outcome assessment (days/months)** | **IG** | **CG** | **p-value of the difference IG vs. CG** |
| Health | Mental component [95% CI] | Baseline | 49.48 [48.22; 50.75] | 50.08 [48.83; 51.33] | 0.29 |
|  |  | After 12 weeks | 50.63 [49.75; 51.52] | 50.70 [49.80; 51.61] | 0.91 |
|  |  | After 6 months | 49.60 [48.71; 50.49] | 49.44 [48.57; 50.31] | 0.80 |
|  | Physical component [95% CI] | Baseline | 52.78 [51.85; 53.71] | 53.30 [52.51; 54.10] | 0.40 |
|  |  | After 12 weeks | 51.63 [50.98; 52.28] | 51.63 [50.96; 52.30] | 0.99 |
|  |  | After 6 months | 52.74 [52.13; 53.35] | 53.45 [52.85; 54.05] | 0.10 |
| Source: Tab. 2 (p. 59) + Tab. 3 (p. 60) | | | | | |

1. Data extraction form – Omran et al. (2018)

Appendix 2. Tab. 4.1 (Omran et al. 2018). Section 1: General review information

| **Item** | **Description** |
| --- | --- |
| Form version/date | Version 1.8, updated November 29, 2016 |
| Review Title | “The impact of financial incentives on physical activity for employees in the context of workplace health promotion - A systematic review” |
| Study ID | Omran (2018) |
| Name of review author completing this form | Miriam Alice Vitzthum |
| Date form completed | 22/09/22 |
| Name of review author checking the data extracted to this form | Dr. Christopher Weyh |
| Author contact details for study | Janine Omran via ResearchGate on 26/08/22; Dr. Guy Faulkner via e-mail ([guy.faulkner@ubc.ca](mailto:guy.faulkner@ubc.ca)) on 22/09/22 |
| Further information required | Study protocol requested |
| Correspondence with authors successful or not; what information was received and when | Correspondence with Dr. Faulkner reveals that there is no pre-published study protocol for this study (reason: “not as common at that time“) (23/09/22).  The following points, which could not be conclusively clarified from the study report, were inquired about in further correspondence (09/24/22):  1. was there any other (than the study protocol) predefined and written form of the study's procedure that I can use to check the risk of bias (e.g., the risk of bias in selection of the reported result)?  2. who funded the study? Can it be ruled out that the funder had influence on the design and conduct of the study, as well as on the preparation of the study report?  3. What are the reasons for the dropout of the participants?  4. How can I interpret the expression "use the action planning tool" (p. 20)? Does it mean that participants had to have completed at least 1 action plan per week?  5. Can you provide me with all the outcome data for both the outcome "engagement with the action planning tool" and the outcome "daily step count"? Unfortunately, there are no tables of all outcome data in the report, only selected outcomes in the body text. For my work it would be important to be able to understand, for example, the changes in step counts in the intervention and control groups during phases II + III as well as phases IV + V (All results of daily step counts used to create Fig. 2).  Dr. Faulkner responded on 01/10/22 as follows:  1. No  2. Not funded  3. Information not collected  4. Yes  5. No, I left this institution and no longer have access to the data. You could try looking for Janine Omran who was the first author. I don’t have her contact details. |
| Will any additional unpublished data supplied by the authors be included in the review?  If so, note that the study will include unpublished data (for entry to RevMan) | - |
| Notes | Information source:  Omran, J.; Trinh, L.; Arbout-Nicitopoulos, K. P.; Mitchell, M.S. & Faulkner, G.E. (2018). Do Incentives Promote Action Planning in a Web-based Walking Intervention? Am J Health Behav., 24 (4), p. 13-22. |

Appendix 2. Tab.4.2 (Omran et al. 2018). Section 2: Methods of the study

| **Item** | **Description** | **Source** |
| --- | --- | --- |
| Aim of study | With this study, the study authors aim to investigate the effect of financial incentives on self-regulatory behavior (e.g., action planning). | p. 13 |
| Study design | This is a randomized controlled trial. | p. 17 |
| Number of arms or group | There are two study arms  Intervention group (IG): intervention + incentive  Control group (CG): intervention | p. 13 |
| Consumer involvement | n. r. | - |
| Funding source | No information on funding sources is provided.  The authors declare that there is no conflict of interest. | p. 21 |
| n. r. = not reported | | |

Appendix 2. Tab. 4.3 (Omran et al. 2018). Section 3: Risk of Bias assessment (modified according to Higgins et al. (2019a))

| **Domain** | **Risk of bias judgement** | **Source** |
| --- | --- | --- |
| bias arising from the randomization process | low risk | see Appendix 3: Assessment of risk of bias – Omran et al. (2018) |
| bias due to deviations from the intended interventions (effect of assignment to intervention) | low risk |  |
| bias due to missing outcome data | low risk |  |
| bias in measurement of the outcome | low risk |  |
| bias in selection of the reported result | low risk |  |
| Overall bias | low risk |  |

Appendix 2. Tab. 4.4 (Omran et al. 2018). Section 4: Study characteristics – Participants

| **Item** | | **Description** | | **Source** |
| --- | --- | --- | --- | --- |
| Description | | 69 employees of a university with an office job participated in the study. | | p. 15 |
| Geographic Location | | Canada | | s. a. |
| Setting | | Canadian University | | s. a. |
| Methods of recruitment of participants | | Through a series of promotional activities at a major Canadian university, participants were recruited for the study in January 2015. | | s. a. |
| Inclusion/Exclusion criteria for participation in study | | Inclusion criteria:   - Internet access   Exclusion Criteria:   - Inability to participate in PA because of a Pre-existing health problem, unless physician approval is obtained - Self-assessed compliance with public health PA guidelines (≥ 150 minutes of moderate to vigorous PA per week or ≥10,000 steps/day). | | s. a. |
| Age: range, mean (SD) | | All: 40.46 (10.6)  IG: 41 (10.39)  CG: 40 (10.93)  Range is not reported. | | p. 13  Tab. 2, p. 18 |
| BMI: mean (SD) | | IG: 24.28 (4.15)  CG: 25.79 (5.59) | | Tab. 2, p. 18 |
| Gender | | All: 88 % female  IG: 91.2 % female  CG: 85.7 % female | | p. 13  Tab. 2, p. 18 |
| Other (social/demographic) details | Education | *≤ High school, Diploma/Certificate*  IG: 11.76 %  CG: 5.71 % | *≥ University*  IG: 88.24 %  CG: 94.29 % | Tab. 2, p. 18 |
|  | Physical activity: mean (SD) | Average daily number of steps  IG: 7148 (2317.52)  CG: 7400 (2992.44) | |  |
| Have important populations or groups been excluded from the study | | n. r. | | - |

| **Numbers involved** | | |
| --- | --- | --- |
| **Study numbers** | **Description** | **Source** |
| Eligible for inclusion | 137 participants assessed for eligibility | Fig. 1, p. 17 |
| Excluded | 68 excluded | s. a. |
| Refused to take part | n. r. | - |
| Randomised to intervention group(s) | There were 69 participants assigned to the two groups.  IG: 34 | s. a. |
| Randomised to control group | CG: 35 | s. a. |
| Excluded post randomisation (for each group; with reasons if relevant) | IG: 0  CG: 0 | s. a. |
| Withdrawn (for each group; with reasons if relevant) | IG: 0  CG: 0 | s. a. |
| Lost to follow up (for each group; with reasons) | Intervention groups (with reasons - n. r.) | s. a. |
|  | Phase 1: n=1  Phase 2: n=0  Phase 3: n=1  Phase 4: n=2  Phase 5: n=6 |  |
|  | Control group (with reasons - n. r.) |  |
|  | Phase 1: n=0  Phase 2: n=0  Phase 3: n=2  Phase 4: n=5  Phase 5: n=2 |  |
| Included in the analysis (for each group, for each outcome) | Included in intention-to-treat analysis:  IG: 34  CG: 35 | s. a. |
| n. r. = not reported; s. a. = see above | | |

Appendix 2. Tab.4.5 (Omran et al. 2018). Section 5: Study characteristics – Interventions (modified according to the current TIDieR checklist (Hoffmann et al., 2014))

| **Item** | **Description** | **Source** |
| --- | --- | --- |
| Item 1. Brief name | Intervention group: experimental condition (incentive). | p. 13 |
| Item 2. Why | Physical activity at work plays a major role in meeting daily activity recommendations, as it has been shown that predominantly sedentary workers find it difficult to compensate via increased physical activity during leisure time. At the same time, the Internet is becoming an increasingly popular place to deliver physical activity and health-related interventions (increase in web-based workplace interventions from 13% to 44%). More and more studies are being conducted that find that financial incentives influence health behaviors, such as physical activity, reinforcing behavioral economics' assumptions about "present bias." However, there are also concerns about financial incentives undermining intrinsic motivation and thus harming the potential for sustained increases in physical activity. The idea of this study, therefore, is to offer the financial incentives not for behavioral outcomes (e.g., daily step count) but for self-regulatory behaviors, which in turn could more sustainably promote the maintenance of physical activity after the incentive period. In this regard, action planning should be financially supported because it has the potential to promote self-efficacy and motivation to engage in physical activity. Action planning is a strategy within self-regulation that helps translate intention to physical activity into action during the volitional phase of behavior change. | pp. 13 + 14 |
| Item 3. What (Materials) | *Action planning tool*: This tool encourages participants to create a detailed outline of the steps they will take to engage in a specific activity. The tool is designed to help participants incorporate short planned walks into their daily routine to achieve the Phase II goal of 2000 steps per day beyond the baseline step count. Participants are guided through a three-step process of choosing an appropriate amount of time for their planned activity, identifying the activity they are interested in, such as walking to work or walking to a meeting, and then completing a "personal walking plan." This plan describes the details of the activity, including: (1) what they plan to do, (2) where they will do the activity, (3) the day and time they will do the activity, and (4) the number of steps they plan to accumulate during the activity. Participants were able to create one action plan per day. In Phase III, obstacle identification and obstacle overcoming plans will be added to the action plan details, and the step goal will be increased by 1000 steps/day.  IG + CG: All participants were provided with a pedometer (Yamax SW-200 (Warminster, PA)) and informed that they are responsible for entering their step count daily on the website.  Participants received a briefing on the study and were instructed on how to use the pedometer based on instructions on a handout and in person when they picked up the pedometer. | p. 16 |
| Item 4. What (Procedures) | Baseline phase (1 week):   - Collection of demographic data (age, gender, height + weight (to calculate BMI), highest level of education) via a self-report questionnaire through the web-based platform. - No change in behavior - Become familiar with self-monitoring using a pedometer and a website to record daily step counts.   Phase I (2 weeks):   - Increase step count by 1000 steps in addition to baseline. - Tips for taking more steps + app to encourage standing breaks available for download - Financial incentives in the form of $5 CAD per action plan per week (see below).   Phase II (2 weeks):   - Increase step count by an additional 1000 steps to 2000 steps in addition to baseline. - Action planning tool + mapped walking routes available at the university - Financial incentives in the form of 5 CAD per action plan per week (see below).   Phase III (2 weeks):   - Increased step count by an additional 1000 steps to 3000 steps in addition to baseline. - Additional features for action planning tool (barriers and coping strategies) + longer walking routes available   Phase IV + V (2 weeks each):   - Maintaining the 3000 steps in addition to the baseline. - Participants are encouraged to continue using all the features offered on the platform.   IG und CG: weekly automated e-mails asking participants to use the program's website for self-monitoring, introducing them to various evidence-based support strategies that could be used during the intervention, and encouraging them to use these support strategies, which were unlocked with each phase, and to participate in the program on an ongoing basis.  IG: In addition to the weekly automated e-mails, all IG participants received an additional e-mail on the first day of Phases II and III specifically notifying them that participants who created and completed at least one new action plan each week in Phase II and Phase III were eligible to receive a 5 CAD electronically delivered gift card (e-gift) to a popular coffee shop on campus. They were also told that individuals who created and completed at least one action plan per week in each week of Phases II and III could receive an additional 5 CAD "perfect planner" e-gift card "bonus" incentive.  "Perfect planners" were participants in the incentive condition who created and completed at least one action plan per week during Phase II and Phase III. Participants received the incentive only if action plans were rated as "complete.". Completion of action plans was verified, and the number of action plans completed by each participant was documented at the end of each week during Phases II to V. An action plan was considered complete if all fields of the action plan were filled in (i.e., what, where, when, number of steps) and the "completed action plan" box was checked. Participants self-reported whether they had completed the action plan.  Immediacy of the financial incentive: The electronic gift card was chosen as an incentive because it provided an easy way to reward participants immediately at the end of each week in Phase II and Phase III (rather than at the end of the intervention), and because it could be redeemed at several cafés on campus. | pp. 15 + 16 |
| Item 5. Who provided | No contact between the participants and the study authors or other intervention providers is necessary for the implementation of the intervention. The e-mails that the participants receive are sent automatically. | p. 16 |
| Item 6. How | The intervention was offered as an individual intervention (individual participants were not dependent on others participating in their group).  There are no reports of contact being made with participants during the intervention. | - |
| Item 7. Where | In addition to the usual work and leisure environment, walking routes near the workplace were presented for participants to use to implement the intervention. | p. 15 |
| Item 8. When and how much | The intervention period lasted ten weeks (phases I-V), during which financial incentives were issued for four weeks (in phases II + III, see above).  During this time, participants were to wear the pedometer from morning to night. Exceptions are bathing, water activities and sleeping. | p. 16  p. 17 |
| Item 9. Tailoring | It is not an individualized intervention, which is why there are no adaptation plans for the planned interventions. | - |
| Item 10. Modifications | No modifications of the intervention are reported during the course of. | - |
| Item 11. How well (planned) | No strategy is reported to maintain treatment adherence. | - |
| Item 12. How well (actual) | n. r. | - |
| n. r. = not reported | | |

Appendix 2. Tab.4.6 (Omran et al. 2018). Section 6: Study characteristics – Outcomes and comparison groups

| **Secondary Outcome** | **Method of assessing outcome measures** | **Method of follow up for non-respondents** | **Timing of outcome assessment**  **(including frequency, length of follow up)** |
| --- | --- | --- | --- |
| Physical activity (daily number of steps) | Pedometer | ITT analysis | Data for the outcome were generated each time participants entered their step count on the website.  The outcome was assessed for the incentive period (phase II + III) and for the post-incentive period (phase IV + V) |
| Source: pp. 15, 16 + 18 | | | |
| Adverse events: No adverse events are reported. | | | |

Appendix 2. Tab.4.7 (Omran et al. 2018). Section 7: Data and results

| **Outcome** | **Timing of outcome assessment)** | **IG** | | **CG** | | **IG vs. CG** |
| --- | --- | --- | --- | --- | --- | --- |
|  |  | **Change in daily step count from baseline; mean (SD)** | **Effect size (cohen's d) at baseline** | **Change in daily step count from baseline; mean (SD)** | **Effect size (cohen's d) at baseline** | **Effect size (cohen's d)** |
| Daily step count | Phase II + III (incentive) | n. r. | n. r. | n. r. | n. r. | d=0.1 (in favor of the IG) |
|  | Phase IV + V (post-incentive) | 1793 (2408.72) | d=0.62 | 686 (2887.62) | d=0.24 | d=0.28 (in favor of the IG) |
| Source: p. 20  n. r. = not reported | | | | | | |

1. Data extraction form – Patel et al. (2016)

Appendix 2. Tab. 5.1 (Patel et al. 2016). Section 1: General review information

| **Item** | **Description** |
| --- | --- |
| Form version/date | Version 1.8, updated November 29, 2016 |
| Review Title | “The impact of financial incentives on physical activity for employees in the context of workplace health promotion - A systematic review” |
| Study ID | Patel (2016) |
| Name of review author completing this form | Miriam Alice Vitzthum |
| Date form completed | 06/10/22 |
| Name of review author checking the data extracted to this form | Dr. Christopher Weyh |
| Author contact details for study | Dr. Mitesh S. Patel via e-mail (mpatel@upenn.edu) on 22/08/22 and on 22/09/22 |
| Further information required | Study protocol requested |
| Correspondence with authors successful or not; what information was received and when | Correspondence not successful |
| Will any additional unpublished data supplied by the authors be included in the review?  If so, note that the study will include unpublished data (for entry to RevMan) | - |
| Notes | Information source:  Patel, M.S.; Asch, D.A.; Rosin, R.; Small, D.S.; Bellamy, S.L.; Heuer, J.; Sproat, S.; Hyson, C.; Haff, N.; Lee, S.M.; Wesby, L.; Hoffer, K.; Shuttleworth, D.; Taylor, D.H.; Hilbert, V.; Zhu, J.; Yang, L.; Wang, X. & Volpp, K.G. (2016). Framing financial incentives to increase physical activity among overweight and obese adults: a randomized, controlled trial. *Ann Intern Med., 164* (6), p. 385–394.  Important: This study report is only available as an "Author Manuscript", which means that the page numbers differ from the source given above. |

Appendix 2. Tab. 5.2 (Patel et al. 2016). Section 2: Methods of the study

| **Item** | **Description** | **Source** |
| --- | --- | --- |
| Aim of study | The goal of the study is to test the effectiveness of three different methods to increase physical activity through financial incentives. | p. 2 |
| Study design | This is a randomized controlled trial. | p. 2 |
| Number of arms or group | There are four experimental arms, including three intervention groups and one control group  CG: daily feedback  IG 1: daily feedback + lottery incentive  IG 2: daily feedback + lottery incentive  IG 3: daily feedback + loss incentive | p. 2 |
| Consumer involvement | n. r. | - |
| Funding source | The study was funded by the National Institute on Aging. Additional support came from the U.S. Department of Veterans Affairs and the Robert Wood Johnson Foundation.  The study authors state that the funders had no influence on the design and conduct of the study; the collection, management, analysis, and interpretation of the data; or the preparation, review, or approval of the manuscript. | pp. 2 + 6 |
| n. r. = not reported | | |

Appendix 2. Tab. 5.3 (Patel et al. 2016). Section 3: Risk of Bias assessment (modified according to Higgins et al. (2019a))

| **Domain** | **Risk of bias judgement** | **Source** |
| --- | --- | --- |
| bias arising from the randomization process | low risk | see Appendix 3: Assessment of risk of bias – Patel et al. (2016) |
| bias due to deviations from the intended interventions (effect of assignment to intervention) | some concerns |  |
| bias due to missing outcome data | low risk |  |
| bias in measurement of the outcome | low risk |  |
| bias in selection of the reported result | some concerns |  |
| Overall bias | some concerns |  |

Appendix 2. Tab.5.4 (Patel et al. 2016). Section 4: Study characteristics – Participants

| **Item** | | **Description** | | | | **Source** |
| --- | --- | --- | --- | --- | --- | --- |
| Description | | 281 employees participated in the study. | | | | p. 3 |
| Geographic Location | | Philadelphia, United States | | | | p. 2 |
| Setting | | University of Pennsylvania | | | | p. 2 |
| Methods of recruitment of participants | | Recruitment was conducted via e-mail to all University of Pennsylvania employees during February-March 2014. All eligible participants provided electronic informed consent, completed a sociodemographic questionnaire, self-reported height and weight, and reported recent physical activity using the long form of the International Physical Activity Questionnaire. | | | | p. 4 |
| Inclusion/Exclusion criteria for participation in study | | Inclusion Criteria:   - Employees of the University of Pennsylvania in Philadelphia. - Aged 18 years or older - BMI ≥ 27 kg/m3   Exclusion Criteria:   - Existing or completed participation in a physical activity study - Unable or unwilling to carry an iPhone (Apple) or Android (Google) smartphone with a mobile application installed - Existing pregnancy/breastfeeding or intent to become pregnant in the next 6 months | | | | p. 4 |
| Age: range, mean (SD) | | All: 39.7 (11.6)  IG 1: 37.1 (10.9)  IG 2: 40.3 (11.2)  IG 3: 41.9 (11.6)  CG: 39.4 (12.2)  Range is not reported. | | | | p. 7 + Tab. 1, p. 16 |
| BMI: mean (SD) | | All: 33.2 (5.6)  IG 1: 32.6 (4.5)  IG 2: 33.1 (5.6)  IG 3: 33.8 (6.8)  CG: 33.2 (5.3) | | | | s. a. |
| Gender | | All: 78% were women  IG 1: 79,7 % female  IG 2: 75,7 % female  IG 3: 77,1 % female  CG: 78,6 % female | | | | s. a. |
| Ethnicity | | White non-Hispanic  IG 1: 59,4 %  IG 2: 62,9 %  IG 3: 70,0 %  CG: 64,3 % | African American non-Hispanic  IG 1: 23,2 %  IG 2: 22,9 %  IG 3: 18,6 %  CG: 22,9 % | Other non-Hispanic  IG 1: 14,5 %  IG 2: 10,0 %  IG 3: 8,6 %  CG: 8,6 % | Hispanic  IG 1:  2,9 %  IG 2:  4,3 %  IG 3:  2,9 %  CG:  4,3 % | Tab. 1, p. 16 |
| Other (social/demographic) details | Education | Less than college  IG 1: 2,9 %  IG 2: 2,9 %  IG 3: 2,9 %  CG: 1,4 % | Some college  IG 1: 21,4 %  IG 2: 18,6 %  IG 3: 17,1 %  CG: 21,4 % | College graduate  IG 1: 75,4 %  IG 2: 78,6 %  IG 3: 80,0 %  CG: 77,1 % | | s. a. |
|  | Physical activity | Median physical activity in the past seven days (MET-min)  IG 1: 2812.2 (1408.0–4806.0)  IG 2: 2785.0 (1336.5–4965.5)  IG 3: 2764.5 (933.0–4456.0)  CG: 2568.8 (1077.0–4941.0) | | | |  |
| Have important populations or groups been excluded from the study | | n. r. | | | | - |

| **Numbers involved** | | | |
| --- | --- | --- | --- |
| **Study numbers** | **Description** | | **Source** |
| Eligible for inclusion | 732 participants assessed for eligibility | | Fig. 1, p. 987 |
| Excluded | 452 excluded | | s. a. |
| Refused to take part | n. r. | | - |
| Randomised to intervention group(s) | 281 participants were assigned to the four groups.  IG 1: 70  IG 2: 71  IG 3: 70 | | s. a. |
| Randomised to control group | CG: 70 | | s. a. |
| Excluded post randomisation (for each group; with reasons if relevant) | IG 1: 1 (enrolment in another physical activity study)  IG 2: 1 (switched to a phone that was not eligible for use)  IG 3: 0  CG: 0 | | s. a. |
| Withdrawn (for each group; with reasons if relevant) | IG 1: 1 (no longer interested)  IG 2: 3 (no longer interested)  IG 3: 3 (no longer interested)  CG: 3 (no longer interested) | | s. a. |
| Lost to follow up (for each group; with reasons) | Intervention groups (with reasons) | | Fig. 1, p. 14 |
|  | After 13 weeks  IG 1: s. a.  IG 2: s. a.  IG 3: s. a. | After 26 weeks  IG 1: 0  IG 2: 2 (no longer interested)  IG 3: 0 |  |
|  | Control group (with reasons) | |  |
|  | After 13 weeks  CG: s. a. | After 26 weeks  CG: 0 |  |
| Included in the analysis (for each group, for each outcome) | Included in intention-to-treat analysis (for each outcome):  IG 1: 69  IG 2: 70  IG 3: 70  CG: 70 | | Fig. 1, p. 14, p. 5 |
| n. r. = not reported, s. a. = see above | | | |

Appendix 2. Tab. 5.5 (Patel et al. 2016). Section 5: Study characteristics – Interventions (modified according to the current TIDieR checklist (Hoffmann et al., 2014))

| **Item** | **Description** | **Source** |
| --- | --- | --- |
| Item 1. Brief name | IG 1: daily feedback + gain incentive.  IG 2: daily feedback + lottery incentive  IG 3: daily feedback + loss incentive.  All groups had the same step goal (7,000 steps/day) and received the same economic value over the intervention period. | pp. 3 + 5 |
| Item 2. Why | To understand why people make decisions that are inconsistent with long-term health goals, the insights of behavioral economics can help. These state that there are certain decision errors that arise, for example, from the tendency for people to be motivated to behave in a certain way by direct rather than delayed rewards, just as they are motivated by losses rather than gains. In addition, people tend to want to avoid feeling regret. All of these findings indicate that the design of financial incentives can have an important impact on effectiveness. Since the optimal design of financial incentives has been poorly researched, this study aims to investigate the effectiveness of three different incentive designs. | p. 3 |
| Item 3. What (Materials) | Prior to the start of the study, potential participants were contacted via e-mail and asked to complete a sociodemographic questionnaire and to indicate their own measurements of height and weight. Furthermore, current physical activity was to be reported via the long form of the International Physical Activitiy Questionnaire.  Participants were provided with an app for their smartphone (Moves smart-phone application (ProtoGeo Oy)) and given a personal identification number ("Each participant was given a unique personal identification number to enter into the smartphone application and verify permission that the study team could access step-count data.").  During the intervention, participants were contacted via the Way to Health platform using messages (via SMS or e-mail).  For enrollment, each participant received 25 USD and for the survey at the end of the intervention (after 13 weeks), each participant received 75 USD. There were no participation incentives for the follow-up period.  The money was given to the participants at the end of the month with all accumulated winnings via bank check. | p. 4 |
| Item 4. What (Procedures) | Participants were told to carry their smartphone with the app installed (e.g., in a pocket, on a belt, or in an armband).  On each day during the 26 weeks of the study, participants in all four study groups were informed whether they had achieved the step goal of 7,000 steps on the previous day.  IG 1: Participants received a USD 1.40 prize (gain incentive) each day of the 13-week intervention period on which they met the step goal.  IG 2: Participants chose a two-digit number between 00 and 99. A winning number was randomly selected each day during the intervention period. If a participant's number matched a single-digit number (an 18% chance), he or she won 5 USD. If the participant's number matched a two-digit number (a 1% chance), he or she won 50 USD. Participants were eligible to receive the reward only if they had reached the 7,000-step goal the previous day. Ineligible participants were told what they would have won if they had met the goal, based on the approach that the desire to avoid regret can be motivating.  IG 3: Participants had USD 1.40 deducted from a pre-allocated monthly amount of 42 USD for each day they failed to meet the step goal (loss incentive)  Incentives were offered only during the 13-week intervention, but daily performance feedback was provided throughout the 26 weeks. | p. 4  p. 5 |
| Item 5. Who provided | Implementation of the intervention requires daily contact from the study authors to inform participants whether they have achieved the step goal on the previous day. | p. 5 |
| Item 6. How | The intervention was offered as an individual intervention (individual participants were not dependent on others participating in their group).  Contact between the study authors and participants was maintained during the intervention and the follow-up period using the platform (Way to Health). They were contacted either by text message or e-mail. | p. 4 |
| Item 7. Where | For the intervention, no other place was necessary than the one where the participants worked and spent their daily lives (steps were recorded not only during working hours, but throughout the day).  „(…) have the phone powered on, and carry it with them (for example, in a pocket or on a belt clip or arm band) while they were active.“ | s. a. |
| Item 8. When and how much | The intervention period was 13 weeks with a subsequent follow-up period of another 13 weeks.  There was an opportunity to win or lose money daily (see above). The accumulated money was handed over at the end of the month by bank check („Participants were mailed a bank check at the end of each month with all accumulated earnings.”). | p. 2  p. 4 |
| Item 9. Tailoring | It is not an individualized intervention, which is why there are no adaptation plans for the planned interventions. | - |
| Item 10. Modifications | No modifications to the interventions are reported. | - |
| Item 11. How well (planned) | A strategy to maintain treatment adherence: “All participants received $25 for enrolling and $75 for participating through the primary end point at 13 weeks along with completion of a survey on their experience. However, there was no participation incentive for the follow-up period.”  No other strategies to maintain treatment adherence or its assessment are reported. | p. 4 |
| Item 12. How well (actual) | After 13 weeks: 10 (3.6%) participants quit the intervention.  After 26 weeks: 12 (4.3%) participants quit the intervention and follow-up period. | Fig. 1, p. 14 |

Appendix 2. Tab.5.6 (Patel et al. 2016). Section 6: Study characteristics – Outcomes and comparison groups

| **Secondary Outcome** | **Method of assessing outcome measures** | **Method of follow up for non-respondents** | **Timing of outcome assessment**  **(including frequency, length of follow up)** |
| --- | --- | --- | --- |
| Daily step count during the intervention and follow-up periods. | Smartphone + Moves smartphone application (ProtoGeo Oy) | ITT analysis | Data for outcome were generated daily by wearing the patient's own smartphone.  At the time point after the intervention (after 13 weeks) and after the follow-up period (after 26 weeks), the outcome was assessed. |
| Source: p. 5, *binary variable: Value 0 if the target was not reached; value 1 if the target was reached. | | | |
| Adverse events: „No adverse events were reported during the entire study.“, (p. 7) | | | |

Appendix 2. Tab. 5.7 (Patel et al. 2016). Section 7: Data and results

| **Out-come** | **Timing of outcome assess-ment** | **IG 1 (gain incentive)** | | **IG 2 (lottery incentive)** | | **IG 3 (loss incentive)** | | **CG** |
| --- | --- | --- | --- | --- | --- | --- | --- | --- |
|  |  | **Mean 95% CI]^+^** | **Difference^#^ to CG [95% CI]; p-value** | **Mean (95% CI]^+^** | **Difference^#^ to CG [95% CI]; p-value** | **Mean [95% CI]^+^** | **Difference^#^ to CG [95% CI]; p-value** | **Mean [95% CI]^+^** |
| Daily step count | After the incentive period (13 weeks) | 5406 [4799; 6013] | 406 [−380; 1193]; p=0.31 | 5251 [4625; 5876] | 245 [−556; 1046]; p=0.55 | 5880 [5265; 6497] | 713 [−78; 1505]; p=0.077 | 5031 [4406; 5656] |
|  | After the follow-up period (26 weeks) | 4692 [4084; 5301] | 30 [−741; 802]; p=0.94 | 4529 [3901; 5158] | −91 [−873; 691]; p=0.82 | 5065 [4466; 5664] | 468 [−293; 1229]; p=0.23 | 4539 [3916; 5162] |
| + Unadjusted data: The days on which no step data are transmitted are not included in the analysis.  # Cleaned data by type of smartphone and with missing data coded as target not reached.  Source: Tab. 2 (p. 17), Tab. 3 (p. 18) + Tab. 4 (p. 19) | | | | | | | | |

1. Data extraction form – Royer et al. (2015)

Appendix 2. Tab. 6.1 (Royer et al. 2015). Section 1: General review information

| **Item** | **Description** |
| --- | --- |
| Form version/date | Version 1.8, updated November 29, 2016 |
| Review Title | “The impact of financial incentives on physical activity for employees in the context of workplace health promotion - A systematic review” |
| Study ID | Royer (2015) |
| Name of review author completing this form | Miriam Alice Vitzthum |
| Date form completed | 24/09/22 |
| Name of review author checking the data extracted to this form | Dr. Christopher Weyh |
| Author contact details for study | The author team via ResearchGate on 8/26/22, and Prof. Heather Royer via e-mail (heather.royer@ucsb.edu) on 9/24/22. |
| Further information required | Study protocol requested |
| Correspondence with authors successful or not; what information was received and when | Correspondence not successful. |
| Will any additional unpublished data supplied by the authors be included in the review?  If so, note that the study will include unpublished data (for entry to RevMan) | - |
| Notes | Information source:  Royer, H.; Stehr, M. & Sydnor, J. (2015). Incentives, Commitments, and Habit Formation in Exercise: Evidence from a Field Experiment with Workers at a Fortune-500 Company. American Economic Journal: Applied Economics, 7(3), p. 51–84.  Further information:  Online appendix, retrieved from:  <https://www.aeaweb.org/aej/app/app/0703/2013-0327_app.pdf>  Authors' disclosure statements, retrieved from: https://www.aeaweb.org/aej/app/ds/0703/2013-0327_ds.zip |

Appendix 2. Tab.6.2 (Royer et al. 2015). Section 2: Methods of the study

| **Item** | **Description** | **Source** |
| --- | --- | --- |
| Aim of study | The purpose of the study is to examine whether the option of a self-funded commitment contract improves the long-term effects of an incentive program on physical activity. | pp. 51 + 52 |
| Study design | This is a randomized controlled trial.  “(…) we randomized individuals into treatment and control groups.“ | p. 56 |
| Number of arms or group | There are two experimental arms, including an intervention group and a control group  CG: no incentives  IG a: incentive-only  IG b: incentive + commitment | pp. 57 + 58 |
| Consumer involvement | n. r. | - |
| Funding source | National Science Foundation grant numbers 0819804 and 1025846, the Upjohn Institute grant number 07-106-10, and the Case Western Reserve University ACES fund grant number 0245054.  The authors declare that there is no conflict of interest (“I hereby declare that I have no relevant or material financial interests that relate to the research described in this paper”). | p. 51  Authors' disclosure statements |
| n. r. = not reported | | |

Appendix 2. Tab.6.3 (Royer et al. 2015). Section 3: Risk of Bias assessment (modified according to Higgins et al. (2019a))

| **Domain** | **Risk of bias judgement** | **Source** |
| --- | --- | --- |
| bias arising from the randomization process | some  concerns | see Appendix 3: Assessment of risk of bias – Royer et al. (2015) |
| bias due to deviations from the intended interventions (effect of assignment to intervention) | some  concerns |  |
| bias due to missing outcome data | high risk |  |
| bias in measurement of the outcome | low risk |  |
| bias in selection of the reported result | some  concerns |  |
| Overall bias | high risk |  |

Appendix 2. Tab. 6.4 (Royer et al. 2015). Section 4: Study characteristics – Participants

| **Item** | | **Description** | | **Source** |
| --- | --- | --- | --- | --- |
| Description | | 1,000 full-time employees participated in the study. These were divided into 15 cohorts and completed the intervention one after the other. | | p. 55 |
| Geographic Location | | USA | | s. a. |
| Setting | | Fortune 500 company in the Midwest | | s. a. |
| Methods of recruitment of participants | | To recruit subjects for each cohort, a random sample of employees was drawn from the company's full list of employees at its headquarters. Employees were then invited via e-mail to participate in two online well-being surveys (initial and follow-up), which were administered five weeks apart. Employees were compensated 25 USD if they participated in both surveys. Due to expected heterogeneity in response to incentives, randomization was stratified into four groups within each cohort: A cross of (i) whether the subject was already a member of the company gym and (ii) whether they indicated in the initial survey that their current training was at or above their personal training goal. | | pp. 55 + 56 |
| Inclusion/Exclusion criteria for participation in study | | Exclusion criteria:   - High-level executives, human resources staff, and fitness studios who were privy to details of the study. | | p. 56 |
| Age: range, mean | | Members  CG: 40.12  IG a: 38.95  IG b: 40.11  Range is not reported. | Non-members  CG: 39.62  IG a: 38.87  IG b: 39.78  Range is not reported. | Tab. 1A + 1B, pp. 60 + 61 |
| Gender | | Members  CG: 54 % female  IG a: 50 % female  IG b: 46 % female | Non-members  CG: 48 % female  IG a: 47 % female  IG b: 49 % female | s. a. |
| BMI | | Members  CG: 28.31  IG a: 27.71  IG b: 28.02 | Non-members  CG: 28.22  IG a: 27.73  IG b: 28.62 | s. a. |
| Other (social/demographic) details | Education (*College degree or more)* | Members  CG: 61 %  IG a: 70 %  IG b: 66 % | Non-members  CG: 64 %  IG a: 67 %  IG b: 74 % | s. a. |
|  | Physical activity *(Average days of overall exercise)* | Members  CG: 3.36  IG a: 3.26  IG b: 3.48 | Non-members  CG: 1.98  IG a: 1.85  IG b: 1.89 |  |
| Have important populations or groups been excluded from the study | | n. r. | | - |

| **Numbers involved** | | |
| --- | --- | --- |
| **Study numbers** | **Description** | **Source** |
| Eligible for inclusion | 1716 participants assessed for eligibility | Online Appendix, Fig. 2 |
| Excluded | 62 excluded  59 e-mail address did not work | s. a. |
| Refused to take part | - | - |
| Randomised to intervention group(s) | 1,000 participants were assigned to the three groups.  IG a: 363  IG b: 347 | s. a. |
| Randomised to control group | CG: 290 | s. a. |
| Excluded post randomisation (for each group; with reasons if relevant) | n. r. | - |
| Withdrawn (for each group; with reasons if relevant) | n. r. | - |
| Lost to follow up (for each group; with reasons) | Intervention groups (with reasons - n. r.) | s. a. |
|  | IG a: 37  IG b: 34 |  |
|  | Control group (with reasons - n. r.) |  |
|  | CG: 15 |  |
| Included in the analysis (for each group, for each outcome) | An intention-to-treat analysis was performed, so it can be assumed that all randomly assigned participants were included in the analysis. | p. 67 |
| n. r. = not reported, s. a. = see above | | |

Appendix 2. Tab.6.5 (Royer et al. 2015). Section 5: Study characteristics – Interventions (modified according to the current TIDieR checklist (Hoffmann et al., 2014))

| **Item** | **Description** | **Source** |
| --- | --- | --- |
| Item 1. Brief name | IG a: incentive-only  IG b: incentive + commitment | pp. 57 + 58 |
| Item 2. Why | The studies dealing with the influence of financial incentives on physical activity point to a problem regarding the long-term effects: After the incentive program ends, participants in the studies fall back into old behavior patterns. The present study addresses whether the design of a self-funded commitment contract could enhance the long-term effects. This works by having participants put a certain amount of money on the line, which they lose if they do not meet the activity goal. This approach is motivated by the theory of time inconsistency. | p. 52 |
| Item 3. What (Materials) | IG a + b for the incentive period: 10 USD for a visit to the company gym (max. 1 visit/day; up to 3 visits/week) and a free gym membership during the incentive period worth 25.92 USD. In addition, new members were offered 20 USD as new membership takes a period of one hour. Subjects were informed of this by both e-mail and a letter sent in the company mail. | p. 57 |
| Item 4. What (Procedures) | Week 1-4 (In-Treatment):  Financial incentives per gym visit (10 USD for a visit to the company gym (max. 1 visit/day; up to 3 visits/week)).  In the last week of the incentive program, all subjects who had participated in the first survey (including the control group) were asked to complete the follow-up survey (largely the same questions as in the first survey (fitness level, exercise habits, subjective well-being, excluding demographic information)).  The use of the gym was measured using the login data. It was not possible to record which activity was performed for how long and at what intensity, as the gym only uses a login procedure and does not require people to log out when they leave.  Week 5-13 (Early Post-Treatment):  At the conclusion of the incentive period, one half of the intervention group was randomly selected and offered the opportunity to set up a self-funded commitment contract in which they could use as much money as they liked. By doing so, they committed to continue using the gym for the eight weeks following the initial incentive period (during which they were not allowed to visit the company's gym for more than 14 consecutive calendar days). If participants kept their commitment, they kept their money - if not, the money was donated to charity.  The offer of a commitment contract was made to participants when they were asked for the mailing address needed for the gym incentives and survey payment.  All incentive payments for gym visits, including those for the incentive-only group, were mailed after the eight-week commitment period, so a subject who chose to enter into a commitment contract did not experience a delay in receiving their incentive payment.  Participants in IGa received an e-mail almost identical to that of IGb, asking them to commit to not missing more than 14 days in a row from the gym in the following eight weeks. However, this e-mail did not mention that money was at stake for this goal. Thus, the difference during the commitment period between IGa and IGb measures the effect of the commitment offer, not the combined effect of the incentive and commitment offer.  Week 14-26 (Late Post-Treatment):  Omission of the commitment contract | pp. 52 + 56  pp. 58 + 59 |
| Item 5. Who provided | For the implementation of the intervention, only the contact via e-mail described above is necessary. Otherwise, there is no contact between the participants and the study authors or other intervention providers. | - |
| Item 6. How | The intervention was offered as an individual intervention (individual participants were not dependent on others participating in their group). | - |
| Item 7. Where | The intervention was conducted in the gym at the headquarters location where participants work. The gym provides showers, lockers, and a towel service. The gym consists of two main rooms: one for classes such as yoga and aerobics and one with fitness equipment (e.g., treadmills, stationary bikes, and weights). | p. 55 |
| Item 8. When and how much | The intervention period spanned two years (February 2009 to March 2011). The intervention was carried out in 15 waves, minimizing the size of the cohorts to exclude gym overload. | p. 55 |
| Item 9. Tailoring | It is not an individually adapted intervention, which is why there are no adaptation plans for the planned interventions. | - |
| Item 10. Modifications | No modifications of the intervention during the study are reported. | - |
| Item 11. How well (planned) | No strategies for maintaining adherence or assessing it are reported. | - |
| Item 12. How well (actual) | - | - |

Appendix 2. Tab.6.6 (Royer et al. 2015). Section 6: Study characteristics – Outcomes and comparison groups

| **Primary Outcome** | **Method of assessing outcome measures** | **Method of follow up for non-respondents** | **Timing of outcome assessment**  ***(including frequency, length of follow up)*** |
| --- | --- | --- | --- |
| Gym visits (measured as "Any Visit" = at least 1 gm visit per week; binary variable, 0=no visit, 1=at least 1 visit/week). | Log-in data to the gym | ITT analysis | Outcome data were generated each time a subject logged into the gym.  Gym visits/week were assessed during the in-treatment period (weeks 1-4), the early posttreatment period (weeks 5-13), and the late posttreatment period (weeks 14-26). |
| Source: pp. 55 + 65 | | | |
| Adverse events: “Because of the low risks involved, the institutional review board did not require collection and reporting of adverse events. (p. 986) | | | |

Appendix 2. Tab. 6.7 (Royer et al. 2015). Section 7: Data and results

| **Primary Outcome** | **Timing of outcome assessment** | **CG**  **Proportion of participants with at least a weekly gym attendance (SE)** | **IG a**  **Proportion of participants with at least a weekly gym attendance (SE)** | **IG b**  **Proportion of participants with at least a weekly gym attendance (SE)** |
| --- | --- | --- | --- | --- |
| Gym visits for all participants | Baseline | 0.20 | -0.01 (0.02) | 0.00 (0.02) |
|  | Weeks 1-4 | 0.02 (0.01) | 0.18*** (0.02) | 0.20*** (0.02) |
|  | Weeks 5-13 | 0.03 (0.01) | 0.04** (0.02) | 0.09*** (0.02) |
|  | Weeks 14-26 | 0.04 (0.02) | 0.04** (0.02) | 0.07*** (0.02) |
|  | Weeks 27-52 | -0.01 (0.01) | 0.02 (0.02) | 0.04*** (0.02) |
|  | Weeks 53-104 | -0.01 (0.02) | 0.02 (0.02) | 0.04** (0.02) |
| Gym visits for members | Baseline | 0.62 | -0.02 (0.05) | 0.01 (0.05) |
|  | Weeks 1-4 | 0.00 (0.03) | 0.23*** (0.04) | 0.21*** (0.04) |
|  | Weeks 5-13 | 0.00 (0.03) | 0.03 (0.03) | 0.10*** (0.03) |
|  | Weeks 14-26 | 0.01 (0.05) | 0.04 (0.04) | 0.08** (0.04) |
|  | Weeks 27-52 | -0.04 (0.04) | -0.01 (0.04) | 0.03 (0.04) |
|  | Weeks 53-104 | -0.06 (0.05) | 0.02 (0.04) | 0.05 (0.04) |
| Gym visits for non-members | Baseline | - | - | - |
|  | Weeks 1-4 | 0.03*** (0.01) | 0.15*** (0.02) | 0.20*** (0.03) |
|  | Weeks 5-13 | 0.05*** (0.01) | 0.04** (0.02) | 0.09*** (0.02) |
|  | Weeks 14-26 | 0.06*** (0.02) | 0.03** (0.02) | 0.06*** (0.02) |
|  | Weeks 27-52 | 0.01 (0.01) | 0.03** (0.01) | 0.05*** (0.02) |
|  | Weeks 53-104 | 0.02* (0.01) | 0.02 (0.02) | 0.04** (0.02) |
| Statistical calculation using regression analysis; *p < 0.1; **p < 0.05; ***p < 0.01.  Source: Tab. 2 (p. 66) | | | | |
