# Supplementary material for: The impact of financial incentives on physical activity for employees in the context of workplace health promotion: a systematic review
Source: J Occup Health. 2024 Aug 19;66(1):uiae048. doi: 10.1093/joccuh/uiae048 (PMC11662443; doi:10.1093/joccuh/uiae048)
Supplement: Web_Material_uiae048 [file web_material_uiae048.zip › Appendix 3 Assessment of risk of bias.docx]

Appendix 3. Assessment of risk of bias (using RoB 2) – Carrera et al. (2020)

Appendix 3. Tab. 1. Study details

| \| Reference \| Carrera, M.; Royer, H.; Stehr, M. & Sydnor, J. (2020). The Structure of Health Incentives: Evidence from a Field Experiment. *Management Science, 66* (5), p. 1890-1908. \| \| --- \| --- \|   Study design   \| X \| Individually-randomized parallel-group trial \| \| --- \| --- \| \| □ \| Cluster-randomized parallel-group trial \| \| □ \| Individually randomized cross-over (or other matched) trial \|   For the purposes of this assessment, the interventions being compared are defined as   \| Experimental: \| Intervention group 1 ("Constant")  Intervention group 2 ("kick-start") \| Comparator: \| Control group \| \| --- \| --- \| --- \| --- \|  \| Specify which outcome is being assessed for risk of bias \| Gym visits (measured as "Any Visit" = at least 1 gym visit per week). \| \| --- \| --- \|  \| Specify the numerical result being assessed. In case of multiple alternative analyses being presented, specify the numeric result (e.g. RR = 1.52 (95 % CI 0.83 to 2.77) and/or a reference (e.g. to a table, figure or paragraph) that uniquely defines the result being assessed. \| Intervention group 1 ("constant"):  Gym visits among non-members (Tab. 4, p. 1901).  Week 1-4: 0.116 (0.027)  week 5-8: 0.106 (0.025)  Week 9-16: 0.036 (0.018)  Gym visits among members (Tab. 5, p. 1903):  Week 1-8: 0.184 (0.048)  Week 9-16: 0.093 (0.047)  Intervention group 2 ("kick-start"):  Gym visits among non-members (Tab. 4, p. 1901).  Week 1-4: 0.113 (0.027)  Week 5-8: 0.074 (0.023)  Week 9-16: 0.030 (0.018)  Gym visits among members (Tab. 5, p. 1903):  Week 1-8: 0.119 (0.044)  Week 9-16: 0.009 (0.043) \| \| --- \| --- \|   Is the review team’s aim for this result…?   \| X \| to assess the effect of *assignment to intervention* (the ‘intention-to-treat’ effect) \| \| --- \| --- \| \| □ \| to assess the effect of *adhering to intervention* (the ‘per-protocol’ effect) \|   If the aim is to assess the effect of *adhering to intervention*, select the deviations from intended intervention that should be addressed (at least one must be checked):  □ occurrence of non-protocol interventions  □ failures in implementing the intervention that could have affected the outcome  □ non-adherence to their assigned intervention by trial participants  Which of the following sources were obtained to help inform the risk-of-bias assessment? (tick as many as apply)  X Journal article(s) with results of the trial  □ Trial protocol  □ Statistical analysis plan (SAP)  □ Non-commercial trial registry record (e.g. ClinicalTrials.gov record)  □ Company-owned trial registry record (e.g. GSK Clinical Study Register record)  □ “Grey literature” (e.g. unpublished thesis)  □ Conference abstract(s) about the trial  □ Regulatory document (e.g. Clinical Study Report, Drug Approval Package)  □ Research ethics application  □ Grant database summary (e.g. NIH RePORTER or Research Councils UK Gateway to Research)  □ Personal communication with trialist  □ Personal communication with the sponsor |
| --- | --- | --- | --- | --- | --- | --- | --- | --- | --- | --- | --- | --- | --- | --- | --- | --- | --- | --- | --- | --- |

Appendix 3. Tab. 2. Domain 1: Risk of bias arising from the randomization process

| **Signalling questions** | **Comments** | **Response options** |
| --- | --- | --- |
| 1.1 Was the allocation sequence random? | NI: The only information about the randomization method is that the study was randomized.  NI | Y / PY / PN / N / NI |
| 1.2 Was the allocation sequence concealed until participants were enrolled and assigned to interventions? |  | Y / PY / PN / N / NI |
| 1.3 Did baseline differences between intervention groups suggest a problem with the randomization process? | N: There are no differences between groups that would indicate irregularities in the randomization process.  (“Only one of 26 p-values testing whether the pretreatment means are equivalent across groups is at or below 0.05.” p. 1897), s. Tab. 2, p. 1898 | Y / PY / PN / N / NI |
| Risk-of-bias judgement | some concerns | low / high / some  concerns |
| Optional: What is the predicted direction of bias arising from the randomization process? | - | NA / Favours experimental / Favours comparator / Towards null /Away from null / Unpredictable |

Appendix 3. 3. Domain 2: Risk of bias due to deviations from the intended interventions (effect of assignment to intervention)

| **Signalling questions** | **Comments** | **Response options** |
| --- | --- | --- |
| 2.1. Were participants aware of their assigned intervention during the trial? | 2.1: PY: “Another issue that could meaningfully affect the interpretation of our results is whether awareness of the treatment patterns, or generally the effects of being in a study, are affecting behavior of our control group or spilling over across treatment groups.”, p. 1905  2.2: NI | Y / PY / PN / N / NI |
| 2.2. Were carers and people delivering the interventions aware of participants' assigned intervention during the trial? |  | Y / PY / PN / N / NI |
| 2.3. If Y/PY/NI to 2.1 or 2.2: Were there deviations from the intended intervention that arose because of the trial context? | NI: No deviations from the planned intervention can be identified from the study report. However, due to the fact that no study protocol and thus not enough information is available, this question cannot be answered conclusively. | NA / Y / PY / PN / N / NI |
| 2.4 If Y/PY to 2.3: Were these deviations likely to have affected the outcome? | - | NA / Y / PY / PN / N / NI |
| 2.5. If Y/PY/NI to 2.4: Were these deviations from intended intervention balanced between groups? | - | NA / Y / PY / PN / N / NI |
| 2.6 Was an appropriate analysis used to estimate the effect of assignment to intervention? | PY: No information is reported on suitable analysis methods that would estimate the effect of assignment to an intervention (e.g., an ITT analysis). However, all participants randomly assigned to the intervention groups are also analyzed in the respective group, which is why it can be assumed that an ITT analysis was performed. (Appendix Fig. A1) | Y / PY / PN / N / NI |
| 2.7 If N/PN/NI to 2.6: Was there potential for a substantial impact (on the result) of the failure to analyse participants in the group to which they were randomized? | - | NA / Y / PY / PN / N / NI |
| Risk-of-bias judgement | some concerns | low / high / some concerns |
| Optional: What is the predicted direction of bias due to deviations from intended interventions? | - | NA / Favours experimental / Favours comparator / Towards null /Away from null / Unpredictable |

Appendix 3. Tab. 4. Domain 2: Risk of bias due to deviations from the intended interventions (effect of adhering to intervention)

| **Signalling questions** | **Comments** | **Response options** |
| --- | --- | --- |
| 2.1. Were participants aware of their assigned intervention during the trial? | - | Y / PY / PN / N / NI |
| 2.2. Were carers and people delivering the interventions aware of participants' assigned intervention during the trial? |  | Y / PY / PN / N / NI |
| 2.3. [If applicable:] If Y/PY/NI to 2.1 or 2.2: Were important non-protocol interventions balanced across intervention groups? | - | NA / Y / PY / PN / N / NI |
| 2.4. [If applicable:] Were there failures in implementing the intervention that could have affected the outcome? | - | NA / Y / PY / PN / N / NI |
| 2.5. [If applicable:] Was there non-adherence to the assigned intervention regimen that could have affected participants’ outcomes? | - | NA / Y / PY / PN / N / NI |
| 2.6. If N/PN/NI to 2.3, or Y/PY/NI to 2.4 or 2.5: Was an appropriate analysis used to estimate the effect of adhering to the intervention? | - | NA / Y / PY / PN / N / NI |
| Risk-of-bias judgement | - | low / high / some concerns |
| Optional: What is the predicted direction of bias due to deviations from intended interventions? | - | NA / Favours experimental / Favours comparator / Towards null /Away from null / Unpredictable |

Appendix 3. Tab. 5. Domain 3: Risk of bias due to missing outcome data

| **Signalling questions** | **Comments** | **Response options** |
| --- | --- | --- |
| 3.1 Were data for this outcome available for all, or nearly all, participants randomized? | N: “Of those 980, a total of 845, or 86 %, filled out the follow-up survey (…)”, p. 1897  According to Higgins et al. (2019)*, an availability of at least 95% of the data is to be classified as "nearly all". | Y / PY / PN / N / NI |
| 3.2 If N/PN/NI to 3.1: Is there evidence that the result was not biased by missing outcome data? | PN: No information is provided on analysis methods that would indicate whether missing outcome data bias the results. | NA / Y / PY / PN / N |
| 3.3 If N/PN to 3.2: Could missingness in the outcome depend on its true value? | NI: No information is provided on why participants dropped out. This also makes it impossible to find out whether their dropout is related to the study content.  NI: No reasons for the absence of outcome data are reported. | NA / Y / PY / PN / N / NI |
| 3.4 If Y/PY/NI to 3.3: Is it likely that missingness in the outcome depended on its true value? |  | NA / Y / PY / PN / N / NI |
| Risk-of-bias judgement | high risk | low / high / some  concerns |
| Optional: What is the predicted direction of bias due to missing outcome data? | - | NA / Favours experimental / Favours comparator / Towards null /Away from null / Unpredictable |
| *Source: Higgins, J. P. T., Savović, J., Page, M. J., & Sterne, J. A. C. (Eds.). (2019a). Revised Cochrane risk-of-bias tool for randomized trials (RoB 2). Retrieval from https://drive.google.com/file/d/19R9savfPdCHC8XLz2iiMvL_71lPJERWK/view?pli=1 | | |

Appendix 3. Tab. 6. Domain 4: Risk of bias in measurement of the outcome

| **Signalling questions** | **Comments** | **Response options** |
| --- | --- | --- |
| 4.1 Was the method of measuring the outcome inappropriate? | N: “When employees enter the gym, they type their company identification number into one of two computer terminals inside the gym’s main entrance. These computerized login records provide high-quality data on visits made to the gym and provide both the data on which program incentives are based and our primary outcome measures.” p. 1895 | Y / PY / PN / N / NI |
| 4.2 Could measurement or ascertainment of the outcome have differed between intervention groups? | N: Gym visits from all participants were recorded via login data to the gym, regardless of group affiliation. | Y / PY / PN / N / NI |
| 4.3 If N/PN/NI to 4.1 and 4.2: Were outcome assessors aware of the intervention received by study participants? | NI: No information about it is provided. | NA / Y / PY / PN / N / NI |
| 4.4 If Y/PY/NI to 4.3: Could assessment of the outcome have been influenced by knowledge of intervention received? | PN: Due to the fact that the results were collected automatically by the login data, the influence by the knowledge about the group membership can be estimated as low. | NA / Y / PY / PN / N / NI |
| 4.5 If Y/PY/NI to 4.4: Is it likely that assessment of the outcome was influenced by knowledge of intervention received? |  | NA / Y / PY / PN / N / NI |
| Risk-of-bias judgement | low risk | low / high / some concerns |
| Optional: What is the predicted direction of bias in measurement of the outcome? | - | NA / Favours experimental / Favours comparator / Towards null /Away from null / Unpredictable |

Appendix 3. Tab. 7. Domain 5: Risk of bias in selection of the reported result

| **Signalling questions** | **Comments** | **Response options** |
| --- | --- | --- |
| 5.1 Were the data that produced this result analysed in accordance with a pre-specified analysis plan that was finalized before unblinded outcome data were available for analysis? | NI: Due to the fact that the study protocol is not available, no statements can be made as to whether the outcome measurements and analyses performed were planned and determined beforehand. Furthermore, it cannot be ensured that the analysis intentions were completed before the outcome data were available. | Y / PY / PN / N / NI |
| Is the numerical result being assessed likely to have been selected, on the basis of the results, from... |  |  |
| 5.2. ... multiple eligible outcome measurements (e.g. scales, definitions, time points) within the outcome domain? | NI | Y / PY / PN / N / NI |
| 5.3 ... multiple eligible analyses of the data? | NI | Y / PY / PN / N / NI |
| Risk-of-bias judgement | some concerns | low / high / some concerns |
| Optional: What is the predicted direction of bias due to selection of the reported result? | - | NA / Favours experimental / Favours comparator / Towards null /Away from null / Unpredictable |

Appendix 3. Tab. 8. Overall risk of bias

| Risk-of-bias judgement | high risk | low / high / some concerns |
| --- | --- | --- |
| Optional: What is the overall predicted direction of bias for this outcome? | - | NA / Favours experimental / Favours comparator / Towards null /Away from null / Unpredictable |

Appendix 3. Tab. 9. Study details

| \| Reference \| Carrera, M.; Royer, H.; Stehr, M. & Sydnor, J. (2020). The Structure of Health Incentives: Evidence from a Field Experiment. *Management Science, 66* (5), p. 1890-1908. \| \| --- \| --- \|   Study design   \| X \| Individually-randomized parallel-group trial \| \| --- \| --- \| \| □ \| Cluster-randomized parallel-group trial \| \| □ \| Individually randomized cross-over (or other matched) trial \|   For the purposes of this assessment, the interventions being compared are defined as   \| Experimental: \| Intervention group 3 ("constant short")  Intervention group 4 ("extended-sporadic") \| Comparator: \| Control group \| \| --- \| --- \| --- \| --- \|  \| Specify which outcome is being assessed for risk of bias \| Gym visits (measured as "Any Visits" = at least 1 gym visit per week). \| \| --- \| --- \|  \| Specify the numerical result being assessed. In case of multiple alternative analyses being presented, specify the numeric result (e.g. RR = 1.52 (95 % CI 0.83 to 2.77) and/or a reference (e.g. to a table, figure or paragraph) that uniquely defines the result being assessed. \| Intervention group 3 (“constant short”)  Gym visits among non-members (Tab. 4, p. 1901).  Week 1-4: 0.064 (0.026)  Week 5-8: 0.027 (0.020)  Week 9-16: 0.017 (0.018)  Intervention group 4 (“extended-sporadic")  Gym visits among members (Tab. 5, p. 1903):  Week 1-8: 0.111 (0.044)  Week 9-16: 0.137 (0.046). \| \| --- \| --- \|   Is the review team’s aim for this result…?   \| X \| to assess the effect of *assignment to intervention* (the ‘intention-to-treat’ effect) \| \| --- \| --- \| \| □ \| to assess the effect of *adhering to intervention* (the ‘per-protocol’ effect) \|   If the aim is to assess the effect of *adhering to intervention*, select the deviations from intended intervention that should be addressed (at least one must be checked):  □ occurrence of non-protocol interventions  □ failures in implementing the intervention that could have affected the outcome  □ non-adherence to their assigned intervention by trial participants  Which of the following sources were obtained to help inform the risk-of-bias assessment? (tick as many as apply)  X Journal article(s) with results of the trial  □ Trial protocol  □ Statistical analysis plan (SAP)  □ Non-commercial trial registry record (e.g. ClinicalTrials.gov record)  □ Company-owned trial registry record (e.g. GSK Clinical Study Register record)  □ “Grey literature” (e.g. unpublished thesis)  □ Conference abstract(s) about the trial  □ Regulatory document (e.g. Clinical Study Report, Drug Approval Package)  □ Research ethics application  □ Grant database summary (e.g. NIH RePORTER or Research Councils UK Gateway to Research)  □ Personal communication with trialist  □ Personal communication with the sponsor |
| --- | --- | --- | --- | --- | --- | --- | --- | --- | --- | --- | --- | --- | --- | --- | --- | --- | --- | --- | --- | --- |

Appendix 3. Tab. 10. Domain 1: Risk of bias arising from the randomization process

| **Signalling questions** | **Comments** | **Response options** |
| --- | --- | --- |
| 1.1 Was the allocation sequence random? | NI: The only information about the randomization method is that the study was randomized .  NI | Y / PY / PN / N / NI |
| 1.2 Was the allocation sequence concealed until participants were enrolled and assigned to interventions? |  | Y / PY / PN / N / NI |
| 1.3 Did baseline differences between intervention groups suggest a problem with the randomization process? | N: There are no differences between groups that would indicate irregularities in the randomization process. (“Only one of 26 p-values testing whether the pretreatment means are equivalent across groups is at or below 0.05.” p. 1897), s. Tab. 2, p. 1898 | Y / PY / PN / N / NI |
| Risk-of-bias judgement | some concerns | low / high / some  concerns |
| Optional: What is the predicted direction of bias arising from the randomization process? | - | NA / Favours experimental / Favours comparator / Towards null /Away from null / Unpredictable |

Appendix 3. 11. Domain 2: Risk of bias due to deviations from the intended interventions (effect of assignment to intervention)

| **Signalling questions** | **Comments** | **Response options** |
| --- | --- | --- |
| 2.1. Were participants aware of their assigned intervention during the trial? | 2.1: PY: “Another issue that could meaningfully affect the interpretation of our results is whether awareness of the treatment patterns, or generally the effects of being in a study, are affecting behavior of our control group or spilling over across treatment groups.”, S. 1905  2.2: NI | Y / PY / PN / N / NI |
| 2.2. Were carers and people delivering the interventions aware of participants' assigned intervention during the trial? |  | Y / PY / PN / N / NI |
| 2.3. If Y/PY/NI to 2.1 or 2.2: Were there deviations from the intended intervention that arose because of the trial context? | Y: In IG 4 ("extended-sporadic"), there were deviations from the planned intervention that arose from the context of the study: After three cohorts had gone through the intervention, the study authors received feedback that, with the longer program duration of 16 weeks (as opposed to the 8 weeks in IG1 and 2), non-members did not want to take up memberships because the fees had to be paid for longer. Therefore, only those who already had a gym membership were assigned to this group, while the non-members were placed in IG3. This was done at the time after 3 out of 15 cohorts had already gone through the program. Therefore, in retrospect, the non-members of the first three cohorts were excluded from IG4 (pp. 1907 + 1908) | NA / Y / PY / PN / N / NI |
| 2.4 If Y/PY to 2.3: Were these deviations likely to have affected the outcome? | N: All participants who were assigned to the groups were also analyzed in them. | NA / Y / PY / PN / N / NI |
| 2.5. If Y/PY/NI to 2.4: Were these deviations from intended intervention balanced between groups? | - | NA / Y / PY / PN / N / NI |
| 2.6 Was an appropriate analysis used to estimate the effect of assignment to intervention? | PY: Although no information is reported on suitable analysis methods that would estimate the effect of assignment to an intervention (e.g., an ITT analysis). However, all participants randomly assigned to the intervention groups are also analyzed in the respective group, which is why it can be assumed that an ITT analysis was performed. (Appendix Fig. A1) | Y / PY / PN / N / NI |
| 2.7 If N/PN/NI to 2.6: Was there potential for a substantial impact (on the result) of the failure to analyse participants in the group to which they were randomized? | - | NA / Y / PY / PN / N / NI |
| Risk-of-bias judgement | some concerns | low / high / some concerns |
| Optional: What is the predicted direction of bias due to deviations from intended interventions? | - | NA / Favours experimental / Favours comparator / Towards null /Away from null / Unpredictable |

Appendix 3. Tab. 12. Domain 2: Risk of bias due to deviations from the intended interventions (effect of adhering to intervention)

| **Signalling questions** | **Comments** | **Response options** |
| --- | --- | --- |
| 2.1. Were participants aware of their assigned intervention during the trial? | - | Y / PY / PN / N / NI |
| 2.2. Were carers and people delivering the interventions aware of participants' assigned intervention during the trial? |  | Y / PY / PN / N / NI |
| 2.3. [If applicable:] If Y/PY/NI to 2.1 or 2.2: Were important non-protocol interventions balanced across intervention groups? | - | NA / Y / PY / PN / N / NI |
| 2.4. [If applicable:] Were there failures in implementing the intervention that could have affected the outcome? | - | NA / Y / PY / PN / N / NI |
| 2.5. [If applicable:] Was there non-adherence to the assigned intervention regimen that could have affected participants’ outcomes? | - | NA / Y / PY / PN / N / NI |
| 2.6. If N/PN/NI to 2.3, or Y/PY/NI to 2.4 or 2.5: Was an appropriate analysis used to estimate the effect of adhering to the intervention? | - | NA / Y / PY / PN / N / NI |
| Risk-of-bias judgement | - | low / high / some concerns |
| Optional: What is the predicted direction of bias due to deviations from intended interventions? | - | NA / Favours experimental / Favours comparator / Towards null /Away from null / Unpredictable |

Appendix 3. Tab. 13. Domain 3: Risk of bias due to missing outcome data

| **Signalling questions** | **Comments** | **Response options** |
| --- | --- | --- |
| 3.1 Were data for this outcome available for all, or nearly all, participants randomized? | N: “Of those 980, a total of 845, or 86 %, filled out the follow-up survey (…)”, p. 1897  According to Higgins et al. (2019)*, an availability of at least 95% of the data is to be classified as "nearly all". | Y / PY / PN / N / NI |
| 3.2 If N/PN/NI to 3.1: Is there evidence that the result was not biased by missing outcome data? | PN: No information is provided on analysis methods that would indicate whether missing outcome data bias the results. | NA / Y / PY / PN / N |
| 3.3 If N/PN to 3.2: Could missingness in the outcome depend on its true value? | NI: No information is provided on why participants dropped out. This also makes it impossible to find out whether their dropout is related to the study content.  NI: no reported reasons for missing outcome data | NA / Y / PY / PN / N / NI |
| 3.4 If Y/PY/NI to 3.3: Is it likely that missingness in the outcome depended on its true value? |  | NA / Y / PY / PN / N / NI |
| Risk-of-bias judgement | high risk | low / high / some  concerns |
| Optional: What is the predicted direction of bias due to missing outcome data? | - | NA / Favours experimental / Favours comparator / Towards null /Away from null / Unpredictable |
| *Source: Higgins, J. P. T., Savović, J., Page, M. J., & Sterne, J. A. C. (Eds.). (2019a). Revised Cochrane risk-of-bias tool for randomized trials (RoB 2). Retrieval from https://drive.google.com/file/d/19R9savfPdCHC8XLz2iiMvL_71lPJERWK/view?pli=1 | | |

Appendix 3. Tab. 14. Domain 4: Risk of bias in measurement of the outcome

| **Signalling questions** | **Comments** | **Response options** |
| --- | --- | --- |
| 4.1 Was the method of measuring the outcome inappropriate? | N: “When employees enter the gym, they type their company identification number into one of two computer terminals inside the gym’s main entrance. These computerized login records provide high-quality data on visits made to the gym and provide both the data on which program incentives are based and our primary outcome measures.”, p. 1895 | Y / PY / PN / N / NI |
| 4.2 Could measurement or ascertainment of the outcome have differed between intervention groups? | N: Gym visits from all participants were recorded via login data to the gym, regardless of group affiliation. | Y / PY / PN / N / NI |
| 4.3 If N/PN/NI to 4.1 and 4.2: Were outcome assessors aware of the intervention received by study participants? | NI: No information is provided about it. | NA / Y / PY / PN / N / NI |
| 4.4 If Y/PY/NI to 4.3: Could assessment of the outcome have been influenced by knowledge of intervention received? | PN: Due to the fact that the results were collected automatically by the login data, the influence by the knowledge about the group membership can be estimated as low. | NA / Y / PY / PN / N / NI |
| 4.5 If Y/PY/NI to 4.4: Is it likely that assessment of the outcome was influenced by knowledge of intervention received? |  | NA / Y / PY / PN / N / NI |
| Risk-of-bias judgement | low risk | low / high / some concerns |
| Optional: What is the predicted direction of bias in measurement of the outcome? | - | NA / Favours experimental / Favours comparator / Towards null /Away from null / Unpredictable |

Appendix 3. Tab. 15. Domain 5: Risk of bias in selection of the reported result

| **Signalling questions** | **Comments** | **Response options** |
| --- | --- | --- |
| 5.1 Were the data that produced this result analysed in accordance with a pre-specified analysis plan that was finalized before unblinded outcome data were available for analysis? | NI: Due to the fact that the study protocol is not available, no statements can be made as to whether the outcome measurements and analyses performed were planned and determined beforehand. Furthermore, it cannot be ensured that the analysis intentions were completed before the outcome data were available. | Y / PY / PN / N / NI |
| Is the numerical result being assessed likely to have been selected, on the basis of the results, from... |  |  |
| 5.2. ... multiple eligible outcome measurements (e.g. scales, definitions, time points) within the outcome domain? | NI | Y / PY / PN / N / NI |
| 5.3 ... multiple eligible analyses of the data? | NI | Y / PY / PN / N / NI |
| Risk-of-bias judgement | some concerns | low / high / some concerns |
| Optional: What is the predicted direction of bias due to selection of the reported result? | - | NA / Favours experimental / Favours comparator / Towards null /Away from null / Unpredictable |

Appendix 3. Tab. 16. Overall risk of bias

| Risk-of-bias judgement | high risk | low / high / some concerns |
| --- | --- | --- |
| Optional: What is the overall predicted direction of bias for this outcome? | - | NA / Favours experimental / Favours comparator / Towards null /Away from null / Unpredictable |

Assessment of risk of bias (using RoB 2) – Finkelstein et al. (2016)

Appendix 3. Tab. 17. Study details

| \| Reference \| Finkelstein, E. A.; Haaland, B. A.; Bilger, M.; Sahasranaman, A.; Sloan, R. A.; Khaing Nang, E. E. & Evenson, K. R. (2016). Effectiveness of activity trackers with and without incentives to increase physical activity (TRIPPA): a randomised controlled trial. Lancet Diabetes & Endocrinology, 4 (12), p. 1-13. \| \| --- \| --- \|   Study design   \| X \| Individually-randomized parallel-group trial \| \| --- \| --- \| \| □ \| Cluster-randomized parallel-group trial \| \| □ \| Individually randomized cross-over (or other matched) trial \|   For the purposes of this assessment, the interventions being compared are defined as   \| Experimental: \| Intervention group 3: FitBit activity tracker + financial incentives. \| Comparator: \| Control group \| \| --- \| --- \| --- \| --- \|  \| Specify which outcome is being assessed for risk of bias \| Physical activity (measured in average number of daily steps) \| \| --- \| --- \|  \| Specify the numerical result being assessed. In case of multiple alternative analyses being presented, specify the numeric result (e.g. RR = 1.52 (95 % CI 0.83 to 2.77) and/or a reference (e.g. to a table, figure or paragraph) that uniquely defines the result being assessed. \| Change in step count IG 3 vs. CG (see Tab. 2+3, pp. 9+10):  after 6 months: 1050 (95% CI [600; 1490]; p<0.0001)  after 12 months: 500 (95% CI [50; 960]; p=0.0289) \| \| --- \| --- \|   Is the review team’s aim for this result…?   \| X \| to assess the effect of *assignment to intervention* (the ‘intention-to-treat’ effect) \| \| --- \| --- \| \| □ \| to assess the effect of *adhering to intervention* (the ‘per-protocol’ effect) \|   If the aim is to assess the effect of *adhering to intervention*, select the deviations from intended intervention that should be addressed (at least one must be checked):  □ occurrence of non-protocol interventions  □ failures in implementing the intervention that could have affected the outcome  □ non-adherence to their assigned intervention by trial participants  Which of the following sources were obtained to help inform the risk-of-bias assessment? (tick as many as apply)  X Journal article(s) with results of the trial  X Trial protocol  □ Statistical analysis plan (SAP)  □ Non-commercial trial registry record (e.g. ClinicalTrials.gov record)  □ Company-owned trial registry record (e.g. GSK Clinical Study Register record)  □ “Grey literature” (e.g. unpublished thesis)  □ Conference abstract(s) about the trial  □ Regulatory document (e.g. Clinical Study Report, Drug Approval Package)  □ Research ethics application  □ Grant database summary (e.g. NIH RePORTER or Research Councils UK Gateway to Research)  □ Personal communication with trialist  □ Personal communication with the sponsor |
| --- | --- | --- | --- | --- | --- | --- | --- | --- | --- | --- | --- | --- | --- | --- | --- | --- | --- | --- | --- | --- |

Appendix 3. Tab. 18. Domain 1: Risk of bias arising from the randomization process

| **Signalling questions** | **Comments** | **Response options** |
| --- | --- | --- |
| 1.1 Was the allocation sequence random? | Y: “We randomly assigned groups […] using a computer generated assignment schedule.” (p. 3)  Y: Envelopes containing information about the study group were prepared by the research team, which were not involved in the randomization process.(p. 3) | Y / PY / PN / N / NI |
| 1.2 Was the allocation sequence concealed until participants were enrolled and assigned to interventions? |  | Y / PY / PN / N / NI |
| 1.3 Did baseline differences between intervention groups suggest a problem with the randomization process? | PN: Report of baselines without p-values (Tab. 1, p. 6) | Y / PY / PN / N / NI |
| Risk-of-bias judgement | low risk | low / high / some  concerns |
| Optional: What is the predicted direction of bias arising from the randomization process? | - | NA / Favours experimental / Favours comparator / Towards null /Away from null / Unpredictable |

Appendix 3. Tab. 19. Domain 2: Risk of bias due to deviations from the intended interventions (effect of assignment to intervention)

| **Signalling questions** | **Comments** | **Response options** |
| --- | --- | --- |
| 2.1. Were participants aware of their assigned intervention during the trial? | Y: “Because of the nature of the intervention, the participants and study coordinator could not be blinded to the study group assignment.” (p. 3) | Y / PY / PN / N / NI |
| 2.2. Were carers and people delivering the interventions aware of participants' assigned intervention during the trial? |  | Y / PY / PN / N / NI |
| 2.3. If Y/PY/NI to 2.1 or 2.2: Were there deviations from the intended intervention that arose because of the trial context? | N: There are no deviations from the planned interventions in the study protocol. | NA / Y / PY / PN / N / NI |
| 2.4 If Y/PY to 2.3: Were these deviations likely to have affected the outcome? | - | NA / Y / PY / PN / N / NI |
| 2.5. If Y/PY/NI to 2.4: Were these deviations from intended intervention balanced between groups? | - | NA / Y / PY / PN / N / NI |
| 2.6 Was an appropriate analysis used to estimate the effect of assignment to intervention? | N: There are no deviations from the planned interventions in the study protocol. | Y / PY / PN / N / NI |
| 2.7 If N/PN/NI to 2.6: Was there potential for a substantial impact (on the result) of the failure to analyse participants in the group to which they were randomized? | - | NA / Y / PY / PN / N / NI |
| Risk-of-bias judgement | low risk | low / high / some concerns |
| Optional: What is the predicted direction of bias due to deviations from intended interventions? | - | NA / Favours experimental / Favours comparator / Towards null /Away from null / Unpredictable |

Appendix 3. Tab. 20. Domain 2: Risk of bias due to deviations from the intended interventions (effect of adhering to intervention)

| **Signalling questions** | **Comments** | **Response options** |
| --- | --- | --- |
| 2.1. Were participants aware of their assigned intervention during the trial? | - | Y / PY / PN / N / NI |
| 2.2. Were carers and people delivering the interventions aware of participants' assigned intervention during the trial? |  | Y / PY / PN / N / NI |
| 2.3. [If applicable:] If Y/PY/NI to 2.1 or 2.2: Were important non-protocol interventions balanced across intervention groups? | - | NA / Y / PY / PN / N / NI |
| 2.4. [If applicable:] Were there failures in implementing the intervention that could have affected the outcome? | - | NA / Y / PY / PN / N / NI |
| 2.5. [If applicable:] Was there non-adherence to the assigned intervention regimen that could have affected participants’ outcomes? | - | NA / Y / PY / PN / N / NI |
| 2.6. If N/PN/NI to 2.3, or Y/PY/NI to 2.4 or 2.5: Was an appropriate analysis used to estimate the effect of adhering to the intervention? | - | NA / Y / PY / PN / N / NI |
| Risk-of-bias judgement | - | low / high / some concerns |
| Optional: What is the predicted direction of bias due to deviations from intended interventions? | - | NA / Favours experimental / Favours comparator / Towards null /Away from null / Unpredictable |

Appendix 3. Tab. 21. Domain 3: Risk of bias due to missing outcome data

| **Signalling questions** | **Comments** | **Response options** |
| --- | --- | --- |
| 3.1 Were data for this outcome available for all, or nearly all, participants randomized? | N: “After 6 months, 57 (7 %) participants were lost to follow-up” (p. 7)  “After 12 months, 153 (19 %) participants were lost to follow-up” (p. 7)  According to Higgins et al. (2019)*, an availability of at least 95% of the data is to be classified as "nearly all". | Y / PY / PN / N / NI |
| 3.2 If N/PN/NI to 3.1: Is there evidence that the result was not biased by missing outcome data? | Y: Multiple imputation was performed. According to Dong & Peng (2013)* this is one way to deal with the problem of missing data (“All statistical analyses were based on estimates pooled across 20 multiple imputed datasets with each complete dataset based on 50 iterations of predictive mean matching imputation of each dependent variable’s missing values conditional on all other variables. Each of the 20 complete datasets was formed by looping across variables for 50 iterations. Within each iteration, each variable’s nonmissing values were regressed on all other variables via stepwise variable selection using the Akaike information criterion, then noisy predictions for the variable’s missing components were generated from the regression model, and matched to their nearest non-missing entry.”, pp. 6+7). | NA / Y / PY / PN / N |
| 3.3 If N/PN to 3.2: Could missingness in the outcome depend on its true value? | - | NA / Y / PY / PN / N / NI |
| 3.4 If Y/PY/NI to 3.3: Is it likely that missingness in the outcome depended on its true value? |  | NA / Y / PY / PN / N / NI |
| Risk-of-bias judgement | low risk | low / high / some concerns |
| Optional: What is the predicted direction of bias due to missing outcome data? | - | NA / Favours experimental / Favours comparator / Towards null /Away from null / Unpredictable |
| *Sources:  Higgins, J. P. T., Savović, J., Page, M. J., & Sterne, J. A. C. (Eds.). (2019a). Revised Cochrane risk-of-bias tool for randomized trials (RoB 2). Retrieval from https://drive.google.com/file/d/19R9savfPdCHC8XLz2iiMvL_71lPJERWK/view?pli=1  Dong, Y., & Peng, C.-Y. J. (2013). Principled missing data methods for researchers. SpringerPlus, 2(1), 222-238. https://doi.org/10.1186/2193-1801-2-222 | | |

Appendix 3. Tab. 22. Domain 4: Risk of bias in measurement of the outcome

| **Signalling questions** | **Comments** | **Response options** |
| --- | --- | --- |
| 4.1 Was the method of measuring the outcome inappropriate? | N: A pedometer (ActiGraph triaxial GT-3x+ accelerometer [ActiGraph, Pensacola, FL, USA]) was used to measure daily steps and was to be worn for at least 10 hours during wakefulness, 7 days/week, p. 4 | Y / PY / PN / N / NI |
| 4.2 Could measurement or ascertainment of the outcome have differed between intervention groups? | N: The measurements were the same between the groups. | Y / PY / PN / N / NI |
| 4.3 If N/PN/NI to 4.1 and 4.2: Were outcome assessors aware of the intervention received by study participants? | N: “team members Involved in assessing outcomes in participants and data analysts were blinded to group assignment.” p. 3 | NA / Y / PY / PN / N / NI |
| 4.4 If Y/PY/NI to 4.3: Could assessment of the outcome have been influenced by knowledge of intervention received? | - | NA / Y / PY / PN / N / NI |
| 4.5 If Y/PY/NI to 4.4: Is it likely that assessment of the outcome was influenced by knowledge of intervention received? |  | NA / Y / PY / PN / N / NI |
| Risk-of-bias judgement | low risk | low / high / some concerns |
| Optional: What is the predicted direction of bias in measurement of the outcome? | - | NA / Favours experimental / Favours comparator / Towards null /Away from null / Unpredictable |

Appendix 3. Tab. 23. Domain 5: Risk of bias in selection of the reported result

| **Signalling questions** | **Comments** | **Response options** |
| --- | --- | --- |
| 5.1 Were the data that produced this result analysed in accordance with a pre-specified analysis plan that was finalized before unblinded outcome data were available for analysis? | Y: The fact that the study protocol was received in November 2014 ensures that analysis intentions were established before unblinded outcome data (June 2013 to August 2015) were available. | Y / PY / PN / N / NI |
| Is the numerical result being assessed likely to have been selected, on the basis of the results, from... |  |  |
| 5.2. ... multiple eligible outcome measurements (e.g. scales, definitions, time points) within the outcome domain? | N: All outcomes reported for this outcome are consistent with the planned outcome measures. | Y / PY / PN / N / NI |
| 5.3 ... multiple eligible analyses of the data? | N: Almost all (see below) reported outcomes eligible for this outcome correspond to all intended analyses.  “Although not expressly stated in the study protocol, we also included results adjusted for wear time in the appendix because we cannot disentangle the competing hypotheses of increased wear time or physical activity, or both.” (p. 7) | Y / PY / PN / N / NI |
| Risk-of-bias judgement | low risk | low / high / some concerns |
| Optional: What is the predicted direction of bias due to selection of the reported result? | - | NA / Favours experimental / Favours comparator / Towards null /Away from null / Unpredictable |

Appendix 3. Tab. 24. Overall risk of bias

| Risk-of-bias judgement | low risk | low / high / some concerns |
| --- | --- | --- |
| Optional: What is the overall predicted direction of bias for this outcome? |  | NA / Favours experimental / Favours comparator / Towards null /Away from null / Unpredictable |

Assessment of risk of bias (using ROBINS-I) – Hunter et al. (2013)

Appendix 3. Tab. 25. ROBINS-I tool (Stage I): At protocol stage

| Specify the review question   \| Participants \| 406 \| \| --- \| --- \| \| Experimental intervention \| PAL-Intervention + financial incentives \| \| Comparator \| PAL-Intervention, no financial incentives \| \| Outcomes \| Primary outcome: physical activity (objectively measured by PA tracking system)  Secondary Outcomes:  Physical activity at work (self-reported using GPAQ).  Health (mental and physical components)  Quality of life (weighted health index from EQ-5D)  Self-efficacy (Physical Activity Self-efficacy Scale)  Work absenteeism (self-reported sick days) \|   List the confounding domains relevant to all or most studies   \| see below \| \| --- \|   List co-interventions that could be different between intervention groups and that could impact on outcomes   \| There are no co-interventions in the present study. \| \| --- \| |
| --- | --- | --- | --- | --- | --- | --- | --- | --- | --- | --- |

Appendix 3. Tab. 26. ROBINS-I tool (Stage II): For each study

| Specify a target randomized trial specific to the study   \| Design \| Individually randomized / Cluster randomized / Matched (e.g. cross-over) \| \| --- \| --- \| \| Participants \| 406 \| \| Experimental intervention \| PAL-Intervention + financial incentives \| \| Comparator \| PAL-Intervention, no financial incentives \|   Is your aim for this study…?   \| X \| to assess the effect of *assignment to* intervention \| \| --- \| --- \| \| □ \| to assess the effect of *starting and adhering to* intervention \|   Specify the outcome  Specify which outcome is being assessed for risk of bias (typically from among those earmarked for the Summary of Findings table). Specify whether this is a proposed benefit or harm of intervention.   \| Physical activity (objectively measured by PA tracking system in minutes). \| \| --- \|   Specify the numerical result being assessed  In case of multiple alternative analyses being presented, specify the numeric result (e.g. RR = 1.52 (95 % CI 0.83 to 2.77) and/or a reference (e.g. to a table, figure or paragraph) that uniquely defines the result being assessed.   \| After 12 weeks:  IG: 17.52 (95% CI [12.49; 22.56];  CG: 16.63 (95% CI [11.76; 21.51]; p=0.59 \| \| --- \| |
| --- | --- | --- | --- | --- | --- | --- | --- | --- | --- | --- | --- | --- | --- | --- |

Appendix 3. Tab. 27. Preliminary consideration of confounders

| Complete a row for each important confounding domain (i) listed in the review protocol; and (ii) relevant to the setting of this particular study, or which the study authors identified as potentially important.  “Important” confounding domains are those for which, in the context of this study, adjustment is expected to lead to a clinically important change in the estimated effect of the intervention. “Validity” refers to whether the confounding variable or variables fully measure the domain, while “reliability” refers to the precision of the measurement (more measurement error means less reliability).   \| (i) Confounding domains listed in the review protocol \| \| \| \| \| \| --- \| --- \| --- \| --- \| --- \| \| Confounding domain \| Measured variable(s) \| Is there evidence that controlling for this variable was unnecessary?* \| Is the confounding domain measured validly and reliably by this variable (or these variables)? \| OPTIONAL: Is failure to adjust for this variable (alone) expected to favour the experimental intervention or the comparator? \| \|  \|  \|  \| Yes / No / No information \| Favour experimental / Favour comparator / No information \| \| Es exisitiert kein Review-Protokoll, in dem Confounding-Domänen gelistet worden wären. \| \| \| \| \|  \| (ii) Additional confounding domains relevant to the setting of this particular study, or which the study authors identified as important \| \| \| \| \| \| --- \| --- \| --- \| --- \| --- \| \| Confounding domain \| Measured variable(s) \| Is there evidence that controlling for this variable was unnecessary?* \| Is the confounding domain measured validly and reliably by this variable (or these variables)? \| OPTIONAL: Is failure to adjust for this variable (alone) expected to favour the experimental intervention or the comparator? \| \|  \|  \|  \| Yes / No / No information \| Favour experimental / Favour comparator / No information \| \| Sociodemographic characteristics \| Age and gender (indicated in the questionnaire)  Education (highest qualification indicated in the questionnaire) \| No \| NI \| NI \| \| Obesity \| BMI (calculated via the values for height and weight given in the questionnaire). \| No \| NI \| NI \| \| Health \| Mental and physical health (indicated via the SF-8 questionnaire). \| No \| NI \| NI \| \| Movement behavior \| GPAQ (Global Physical Activity Questionnaire)  Physical activity at work (in minutes/week)  Moderate to high physical activity (minutes/week)  Physical activity categories (high, moderate, low) \| No \| Yes (pp. 61+62) \| YES, favour comparator \|   * In the context of a particular study, variables can be demonstrated not to be confounders and so not included in the analysis: (a) if they are not predictive of the outcome; (b) if they are not predictive of intervention; or (c) because adjustment makes no or minimal difference to the estimated effect of the primary parameter. Note that “no statistically significant association” is not the same as “not predictive”. |
| --- | --- | --- | --- | --- | --- | --- | --- | --- | --- | --- | --- | --- | --- | --- | --- | --- | --- | --- | --- | --- | --- | --- | --- | --- | --- | --- | --- | --- | --- | --- | --- | --- | --- | --- | --- | --- | --- | --- | --- | --- | --- | --- | --- | --- | --- | --- | --- | --- | --- | --- | --- | --- | --- | --- | --- |

Appendix 3. Tab. 28. Preliminary consideration of co-interventions

| Complete a row for each important co-intervention (i) listed in the review protocol; and (ii) relevant to the setting of this particular study, or which the study authors identified as important.  “Important” co-interventions are those for which, in the context of this study, adjustment is expected to lead to a clinically important change in the estimated effect of the intervention.   \| (i) Co-interventions listed in the review protocol \| \| \| \| --- \| --- \| --- \| \| Co-intervention \| Is there evidence that controlling for this co-intervention was unnecessary (e.g. because it was not administered)? \| Is presence of this co-intervention likely to favour outcomes in the experimental intervention or the comparator \| \|  \|  \| Favour experimental / Favour comparator / No information \| \|  \|  \| Favour experimental / Favour comparator / No information \|  \| (ii) Additional co-interventions relevant to the setting of this particular study, or which the study authors identified as important \| \| \| \| --- \| --- \| --- \| \| Co-intervention \| Is there evidence that controlling for this co-intervention was unnecessary (e.g. because it was not administered)? \| Is presence of this co-intervention likely to favour outcomes in the experimental intervention or the comparator \| \|  \|  \| Favour experimental / Favour comparator / No information \| |
| --- | --- | --- | --- | --- | --- | --- | --- | --- | --- | --- | --- | --- | --- | --- | --- | --- | --- | --- | --- | --- | --- |

Appendix 3. Tab. 29. Domain 1: Risk of bias due to confounding

| **Signalling questions** | **Description** | **Response options** |
| --- | --- | --- |
| 1.1 Is there potential for confounding of the effect of intervention in this study?  If N/PN to 1.1: the study can be considered to be at low risk of bias due to confounding and no further signalling questions need be considered | PN: Tab. 2 (p. 59) shows that the variable GPAQ: minutes of work PA (minutes/week) has a significant baseline difference between the two groups. However, it is unlikely that the significantly higher activity level in the control group could have an effect on the intervention, since the IG and CG were classified according to buildings A and B of the workplace (p. 57) | Y / PY / PN / N |
| If Y/PY to 1.1: determine whether there is a need to assess time-varying confounding: |  |  |
| 1.2. Was the analysis based on splitting participants’ follow up time according to intervention received?  If N/PN, answer questions relating to baseline confounding (1.4 to 1.6)  If Y/PY, go to question 1.3. | - | NA / Y / PY / PN / N / NI |
| 1.3. Were intervention discontinuations or switches likely to be related to factors that are prognostic for the outcome?  If N/PN, answer questions relating to baseline confounding (1.4 to 1.6)  If Y/PY, answer questions relating to both baseline and time-varying confounding (1.7 and 1.8) | - | NA / Y / PY / PN / N / NI |
| Questions relating to baseline confounding only | | |
| 1.4. Did the authors use an appropriate analysis method that controlled for all the important confounding domains? | - | NA / Y / PY / PN / N / NI |
| 1.5. If Y/PY to 1.4: Were confounding domains that were controlled for measured validly and reliably by the variables available in this study? | - | NA / Y / PY / PN / N / NI |
| 1.6. Did the authors control for any post-intervention variables that could have been affected by the intervention? | - | NA / Y / PY / PN / N / NI |
| Questions relating to baseline and time-varying confounding | |  |
| 1.7. Did the authors use an appropriate analysis method that controlled for all the important confounding domains and for time-varying confounding? | - | NA / Y / PY / PN / N / NI |
| 1.8. If Y/PY to 1.7: Were confounding domains that were controlled for measured validly and reliably by the variables available in this study? | - | NA / Y / PY / PN / N / NI |
| Risk of bias judgement | low risk | low / moderate / serious / critical / NI |
| Optional: What is the predicted direction of bias due to confounding? | - | Favours experimental / Favours comparator / Unpredictable |

Appendix 3. Tab. 30. Domain 2: Risk of bias in selection of participants into the study

| **Signalling questions** | **Description** | **Response options** |
| --- | --- | --- |
| 2.1. Was selection of participants into the study (or into the analysis) based on participant characteristics observed after the start of intervention?  If N/PN to 2.1: go to 2.4 | N: The selection of participants for the study was determined based on the inclusion and exclusion criteria prior to the start of the intervention. (p. 57) | Y / PY / PN / N / NI |
| 2.2. If Y/PY to 2.1: Were the post-intervention variables that influenced selection likely to be associated with intervention?  2.3 If Y/PY to 2.2: Were the post-intervention variables that influenced selection likely to be influenced by the outcome or a cause of the outcome? | - | NA / Y / PY / PN / N / NI  NA / Y / PY / PN / N / NI |
| 2.4. Do start of follow up and start of intervention coincide for most participants? | Y: All participants start the intervention at the same time and the follow-up period at the same time. | Y / PY / PN / N / NI |
| 2.5. If Y/PY to 2.2 and 2.3, or N/PN to 2.4: Were adjustment techniques used that are likely to correct for the presence of selection biases? | - | NA / Y / PY / PN / N / NI |
| Risk of bias judgement | low risk | low / moderate / serious / critical / NI |
| Optional: What is the predicted direction of bias due to selection of participants into the study? | - | Favours experimental / Favours comparator / Towards null /Away from null / Unpredictable |

Appendix 3. Tab. 31. Domain 3: Risk of bias in classification of interventions

| **Signalling questions** | **Description** | **Response options** |
| --- | --- | --- |
| 3.1 Were intervention groups clearly defined? | Y: The interventions are precisely defined (p. 58) | Y / PY / PN / N / NI |
| 3.2 Was the information used to define intervention groups recorded at the start of the intervention? | PY: No study protocol exists, making it impossible to conclusively determine whether information about the interventions could have been influenced by subsequent outcomes. | Y / PY / PN / N / NI |
| 3.3 Could classification of intervention status have been affected by knowledge of the outcome or risk of the outcome? | PN: There is no evidence to suggest that the classification of intervention status was influenced by knowledge of the outcome. | Y / PY / PN / N / NI |
| Risk of bias judgement | moderate risk | low / moderate / serious / critical / NI |
| Optional: What is the predicted direction of bias due to classification of interventions? | - | Favours experimental / Favours comparator / Towards null /Away from null / Unpredictable |

Appendix 3. Tab. 32. Domain 4: Risk of bias due to deviations from intended interventions

| **Signalling questions** | **Description** | **Response options** |
| --- | --- | --- |
| If your aim for this study is to assess the effect of assignment to intervention, answer questions 4.1 and 4.2 | |  |
| 4.1. Were there deviations from the intended intervention beyond what would be expected in usual practice? | PN: No deviations from the planned intervention can be identified from the study report. However, no study protocol is available and thus not enough information, so that this question cannot be answered conclusively. | Y / PY / PN / N / NI |
| 4.2. If Y/PY to 4.1: Were these deviations from intended intervention unbalanced between groups *and* likely to have affected the outcome? | - | NA / Y / PY / PN / N / NI |
| If your aim for this study is to assess the effect of starting and adhering to intervention, answer questions 4.3 to 4.6 | |  |
| 4.3. Were important co-interventions balanced across intervention groups? | - | Y / PY / PN / N / NI |
| 4.4. Was the intervention implemented successfully for most participants? | - | Y / PY / PN / N / NI |
| 4.5. Did study participants adhere to the assigned intervention regimen? | - | Y / PY / PN / N / NI |
| 4.6. If N/PN to 4.3, 4.4 or 4.5: Was an appropriate analysis used to estimate the effect of starting and adhering to the intervention? | - | NA / Y / PY / PN / N / NI |
| Risk of bias judgement | low risk | low / moderate / serious / critical / NI |
| Optional: What is the predicted direction of bias due to deviations from the intended interventions? | - | Favours experimental / Favours comparator / Towards null /Away from null / Unpredictable |

Appendix 3. 33. Domain 5: Risk of bias due to missing data

| **Signalling questions** | **Description** | **Response options** |
| --- | --- | --- |
| 5.1 Were outcome data available for all, or nearly all, participants? | PN: “84 % of employees in both groups completed the study at 6 months follow up.”, p. 59  However, this statement does not clarify how much outcome data was actually available for physical activity, because the 16 % dropout could be attributed to both the intervention and the follow-up time (for the secondary outcomes). Table 3 (p. 60) also does not provide any further information, as it only shows that all randomized participants were included in the analysis of the data for physical activity (ITT analysis).  There is no information on how much outcome data is really available after 12 weeks. | Y / PY / PN / N / NI |
| 5.2 Were participants excluded due to missing data on intervention status? | NI | Y / PY / PN / N / NI |
| 5.3 Were participants excluded due to missing data on other variables needed for the analysis? | NI | Y / PY / PN / N / NI |
| 5.4 If PN/N to 5.1, or Y/PY to 5.2 or 5.3: Are the proportion of participants and reasons for missing data similar across interventions? | NI: Only the secondary outcome data show that 31 participants in the IG and 32 participants in the CG dropped out. Reasons are not given. | NA / Y / PY / PN / N / NI |
| 5.5 If PN/N to 5.1, or Y/PY to 5.2 or 5.3: Is there evidence that results were robust to the presence of missing data? | NI: There is no information on how the missing data was handled in the analysis. | NA / Y / PY / PN / N / NI |
| Risk of bias judgement | no information | low / moderate / serious / critical / NI |
| Optional: What is the predicted direction of bias due to missing data? |  | Favours experimental / Favours comparator / Towards null /Away from null / Unpredictable |

Appendix 3. Tab. 34. Domain 6: Risk of bias in measurement of outcomes

| **Signalling questions** | **Description** | **Response options** |
| --- | --- | --- |
| 6.1 Could the outcome measure have been influenced by knowledge of the intervention received? | PN: Participants knew their own intervention (which was not at all possible otherwise in this study), but they did not know the intervention that the other group received (the intervention and control groups were split between the two buildings), p. 57 | Y / PY / PN / N / NI |
| 6.2 Were outcome assessors aware of the intervention received by study participants? | N: “(…) in an assessor-blind quasi-experimental design(…)”. p. 57 | Y / PY / PN / N / NI |
| 6.3 Were the methods of outcome assessment comparable across intervention groups? | Y: The method was the same for the IG and the CG, p. 58 | Y / PY / PN / N / NI |
| 6.4 Were any systematic errors in measurement of the outcome related to intervention received? | PN: Based on the fact that the evaluators of the outcome were unaware of the intervention received and that the methods used to evaluate the outcome were comparable, it is reasonable to assume that there were no systematic errors in outcome measurement related to the intervention received. | Y / PY / PN / N / NI |
| Risk of bias judgement | moderate risk | low / moderate / serious / critical / NI |
| Optional: What is the predicted direction of bias due to measurement of outcomes? | - | Favours experimental / Favours comparator / Towards null /Away from null / Unpredictable |

Appendix 3. Tab. 35. Domain 7: Risk of bias in selection of the reported result

| **Signalling questions** | **Description** | **Response options** |
| --- | --- | --- |
| Is the reported effect estimate likely to be selected, on the basis of the results, from... |  |  |
| 7.1. ... multiple outcome *measurements* within the outcome domain? | N: For the outcome physical activity (objectively measured by PA tracking system), there is only one measurement that is also reported. | Y / PY / PN / N / NI |
| 7.2 ... multiple *analyses* of the intervention-outcome relationship? | N: Only one analysis process is reported, which also clarifies what happens when someone does not use their PAL card. (p. 59) | Y / PY / PN / N / NI |
| 7.3 ... different *subgroups*? | N: No effect estimates for subgroups are reported. | Y / PY / PN / N / NI |
| Risk of bias judgement | low risk | low / moderate / serious / critical / NI |
| Optional: What is the predicted direction of bias due to selection of the reported result? | - | Favours experimental / Favours comparator / Towards null /Away from null / Unpredictable |

Appendix 3. Tab. 36. Overall risk of bias

| Risk of bias judgement | moderate risk | low / moderate / serious / critical / NI |
| --- | --- | --- |
| Optional: What is the overall predicted direction of bias for this outcome? | - | Favours experimental / Favours comparator / Towards null /Away from null / Unpredictable |

Assessment of risk of bias (using RoB 2) – Omran et al. (2018)

Appendix 3. Tab. 37. Study details

| \| Reference \| Omran, J.; Trinh, L.; Arbout-Nicitopoulos, K. P.; Mitchell, M.S. & Faulkner, G.E. (2018). Do Incentives Promote Action Planning  in a Web-based Walking Intervention? Am J Health Behav., 24 (4), p. 13-22 \| \| --- \| --- \|   Study design   \| X \| Individually-randomized parallel-group trial \| \| --- \| --- \| \| □ \| Cluster-randomized parallel-group trial \| \| □ \| Individually randomized cross-over (or other matched) trial \|   For the purposes of this assessment, the interventions being compared are defined as   \| Experimental: \| Intervention group: incentive \| Comparator: \| Control group: No incentive \| \| --- \| --- \| --- \| --- \|  \| Specify which outcome is being assessed for risk of bias \| Daily step count \| \| --- \| --- \|  \| Specify the numerical result being assessed. In case of multiple alternative analyses being presented, specify the numeric result (e.g. RR = 1.52 (95 % CI 0.83 to 2.77) and/or a reference (e.g. to a table, figure or paragraph) that uniquely defines the result being assessed. \| Change in daily step count from baseline; mean (SD):  After 4 weeks (incentive period)  IG: not reported  CG: not reported  After 8 weeks (post-incentive period)  IG: 1793 (2408.72)  CG: 686 (2887.62) \| \| --- \| --- \|   Is the review team’s aim for this result…?   \| X \| to assess the effect of *assignment to intervention* (the ‘intention-to-treat’ effect) \| \| --- \| --- \| \| □ \| to assess the effect of *adhering to intervention* (the ‘per-protocol’ effect) \|   If the aim is to assess the effect of *adhering to intervention*, select the deviations from intended intervention that should be addressed (at least one must be checked):  □ occurrence of non-protocol interventions  □ failures in implementing the intervention that could have affected the outcome  □ non-adherence to their assigned intervention by trial participants  Which of the following sources were obtained to help inform the risk-of-bias assessment? (tick as many as apply)  X Journal article(s) with results of the trial  □ Trial protocol  □ Statistical analysis plan (SAP)  □ Non-commercial trial registry record (e.g. ClinicalTrials.gov record)  □ Company-owned trial registry record (e.g. GSK Clinical Study Register record)  □ “Grey literature” (e.g. unpublished thesis)  □ Conference abstract(s) about the trial  □ Regulatory document (e.g. Clinical Study Report, Drug Approval Package)  □ Research ethics application  □ Grant database summary (e.g. NIH RePORTER or Research Councils UK Gateway to Research)  X Personal communication with trialist  □ Personal communication with the sponsor |
| --- | --- | --- | --- | --- | --- | --- | --- | --- | --- | --- | --- | --- | --- | --- | --- | --- | --- | --- | --- | --- |

Appendix 3. Tab. 38. Domain 1: Risk of bias arising from the randomization process

| **Signalling questions** | **Comments** | **Response options** |
| --- | --- | --- |
| 1.1 Was the allocation sequence random? | Y: “Participants (N = 69) were randomly assigned with equal allocation (1:1) (…) using a computer-generated random numbers list.”, p. 16  Y: “A research assistant generated the condition assignments, which were concealed from the program administrator.”, p. 16 | Y / PY / PN / N / NI |
| 1.2 Was the allocation sequence concealed until participants were enrolled and assigned to interventions? |  | Y / PY / PN / N / NI |
| 1.3 Did baseline differences between intervention groups suggest a problem with the randomization process? | N: “No differences in demographic characteristics or the average steps taken per day during baseline between conditions were found (Table 2).”, p. 19 | Y / PY / PN / N / NI |
| Risk-of-bias judgement | low risk | low / high / some concerns |
| Optional: What is the predicted direction of bias arising from the randomization process? | - | NA / Favours experimental / Favours comparator / Towards null /Away from null / Unpredictable |

Appendix 3. Tab. 39. Domain 2: Risk of bias due to deviations from the intended interventions (effect of assignment to intervention)

| **Signalling questions** | **Comments** | **Response options** |
| --- | --- | --- |
| 2.1. Were participants aware of their assigned intervention during the trial? | PY: There is no explicit statement about this in the study report. However, it is already inherent in the intervention that it cannot be avoided that the participants and study coordinators know about the allocation. | Y / PY / PN / N / NI |
| 2.2. Were carers and people delivering the interventions aware of participants' assigned intervention during the trial? |  | Y / PY / PN / N / NI |
| 2.3. If Y/PY/NI to 2.1 or 2.2: Were there deviations from the intended intervention that arose because of the trial context? | NI: No deviations from the planned intervention can be identified from the study report. However, since no study protocol exists, this question cannot be answered conclusively. | NA / Y / PY / PN / N / NI |
| 2.4 If Y/PY to 2.3: Were these deviations likely to have affected the outcome? | - | NA / Y / PY / PN / N / NI |
| 2.5. If Y/PY/NI to 2.4: Were these deviations from intended intervention balanced between groups? | - | NA / Y / PY / PN / N / NI |
| 2.6 Was an appropriate analysis used to estimate the effect of assignment to intervention? | Y: “This procedure conservatively assumed no change in variables and allowed analysis by intention-to-treat (ITT).”, p. 18 | Y / PY / PN / N / NI |
| 2.7 If N/PN/NI to 2.6: Was there potential for a substantial impact (on the result) of the failure to analyse participants in the group to which they were randomized? | - | NA / Y / PY / PN / N / NI |
| Risk-of-bias judgement | some concerns | low / high / some concerns |
| Optional: What is the predicted direction of bias due to deviations from intended interventions? | - | NA / Favours experimental / Favours comparator / Towards null /Away from null / Unpredictable |

Appendix 3. Tab. 40. Domain 2: Risk of bias due to deviations from the intended interventions (effect of adhering to intervention)

| **Signalling questions** | **Comments** | **Response options** |
| --- | --- | --- |
| 2.1. Were participants aware of their assigned intervention during the trial? | - | Y / PY / PN / N / NI |
| 2.2. Were carers and people delivering the interventions aware of participants' assigned intervention during the trial? |  | Y / PY / PN / N / NI |
| 2.3. [If applicable:] If Y/PY/NI to 2.1 or 2.2: Were important non-protocol interventions balanced across intervention groups? | - | NA / Y / PY / PN / N / NI |
| 2.4. [If applicable:] Were there failures in implementing the intervention that could have affected the outcome? | - | NA / Y / PY / PN / N / NI |
| 2.5. [If applicable:] Was there non-adherence to the assigned intervention regimen that could have affected participants’ outcomes? | - | NA / Y / PY / PN / N / NI |
| 2.6. If N/PN/NI to 2.3, or Y/PY/NI to 2.4 or 2.5: Was an appropriate analysis used to estimate the effect of adhering to the intervention? | - | NA / Y / PY / PN / N / NI |
| Risk-of-bias judgement | - | low / high / some concerns |
| Optional: What is the predicted direction of bias due to deviations from intended interventions? | - | NA / Favours experimental / Favours comparator / Towards null /Away from null / Unpredictable |

Appendix 3. Tab. 41. Domain 3: Risk of bias due to missing outcome data

| **Signalling questions** | **Comments** | **Response options** |
| --- | --- | --- |
| 3.1 Were data for this outcome available for all, or nearly all, participants randomized? | N: The dropout rate ranges from 71% (incentive) to 74% (no incentive), p. 19.  According to Higgins et al. (2019)*, an availability of at least 95% of the data is to be classified as "nearly all". | Y / PY / PN / N / NI |
| 3.2 If N/PN/NI to 3.1: Is there evidence that the result was not biased by missing outcome data? | N: “Missing data patterns were assessed using Little’s MCAR test to identify if step count entries were missing at random; however, no missing step count data were imputed.”, p. 18: Only how to deal with missing step data, e.g. due to technical malfunction of the pedometers, is described, not how to deal with missing data from dropped out subjects. | NA / Y / PY / PN / N |
| 3.3 If N/PN to 3.2: Could missingness in the outcome depend on its true value? | NI: No information is provided on why participants dropped out. This also makes it impossible to find out whether their dropout is related to the study content.  NI: no reported reasons for missing outcome data. | NA / Y / PY / PN / N / NI |
| 3.4 If Y/PY/NI to 3.3: Is it likely that missingness in the outcome depended on its true value? |  | NA / Y / PY / PN / N / NI |
| Risk-of-bias judgement | high risk | low / high / some concerns |
| Optional: What is the predicted direction of bias due to missing outcome data? | - | NA / Favours experimental / Favours comparator / Towards null /Away from null / Unpredictable |
| *Source: Higgins, J. P. T., Savović, J., Page, M. J., & Sterne, J. A. C. (Eds.). (2019a). Revised Cochrane risk-of-bias tool for randomized trials (RoB 2). Retrieval from https://drive.google.com/file/d/19R9savfPdCHC8XLz2iiMvL_71lPJERWK/view?pli=1 | | |

Appendix 3. Tab. 42. Domain 4: Risk of bias in measurement of the outcome

| **Signalling questions** | **Comments** | **Response options** |
| --- | --- | --- |
| 4.1 Was the method of measuring the outcome inappropriate? | PN: “All participants were provided with a Yamax SW-200 pedometer (Warminster, PA) and were responsible for logging their step counts daily on the website.”, p. 16. “was the most accurate at detecting steps taken”, p. 17.  “Participants self-reported if they had completed the action plan.”, p. 17  Doubts in the measurement methodology exist due to the fact that participants were responsible for entering their daily step count on the website and indicating whether they implemented their action plan. | Y / PY / PN / N / NI |
| 4.2 Could measurement or ascertainment of the outcome have differed between intervention groups? | N: "All participants were provided with a Yamax SW-200 pedometer (Warminster, PA) and were responsible for logging their step counts daily on the website.”, p. 16. | Y / PY / PN / N / NI |
| 4.3 If N/PN/NI to 4.1 and 4.2: Were outcome assessors aware of the intervention received by study participants? | Y: Participants know if they are getting incentives or not and at the same time enter their daily step count in the website.  “For participant-reported outcomes, the outcome assessor is the study participant.” (Higgins et al., 2019)* | NA / Y / PY / PN / N / NI |
| 4.4 If Y/PY/NI to 4.3: Could assessment of the outcome have been influenced by knowledge of intervention received? | 4.4 PY: Due to the fact that no evaluation takes place, as for example in the assessment of pain, the influence of the knowledge about the intervention can be estimated as low. However, a residual uncertainty remains, because it was not independently verified whether the entered step count values really corresponded to the objectively measured step counts on the pedometers.  “Knowledge of the assigned intervention could influence participant-reported outcomes (such as level of pain), observer-reported outcomes involving some judgement, and intervention provider decision outcomes. They are unlikely to influence observer-reported outcomes that do not involve judgement, for example all-cause mortality.” (Higgins et al., 2019, p. 54)*  ---  4.5 PN: There is no reason to believe that knowledge of intervention status influenced outcome and that participants specifically entered a different step count than that reported on the pedometer. | NA / Y / PY / PN / N / NI |
| 4.5 If Y/PY/NI to 4.4: Is it likely that assessment of the outcome was influenced by knowledge of intervention received? |  | NA / Y / PY / PN / N / NI |
| Risk-of-bias judgement | some concerns | low / high / some concerns |
| Optional: What is the predicted direction of bias in measurement of the outcome? |  | NA / Favours experimental / Favours comparator / Towards null /Away from null / Unpredictable |
| *Source: Higgins, J. P. T., Savović, J., Page, M. J., & Sterne, J. A. C. (Eds.). (2019a). Revised Cochrane risk-of-bias tool for randomized trials (RoB 2). Retrieval from https://drive.google.com/file/d/19R9savfPdCHC8XLz2iiMvL_71lPJERWK/view?pli=1 | | |

Appendix 3. Tab. 43. Domain 5: Risk of bias in selection of the reported result

| **Signalling questions** | **Comments** | **Response options** |
| --- | --- | --- |
| 5.1 Were the data that produced this result analysed in accordance with a pre-specified analysis plan that was finalized before unblinded outcome data were available for analysis? | NI: Due to the fact that no study protocol exists, no statements can be made as to whether the outcome measurements and analyses performed were planned and determined beforehand. Furthermore, it cannot be ensured that the analysis intentions were completed before the outcome data were available. | Y / PY / PN / N / NI |
| Is the numerical result being assessed likely to have been selected, on the basis of the results, from... |  |  |
| 5.2. ... multiple eligible outcome measurements (e.g. scales, definitions, time points) within the outcome domain? | PN: The outcome (action plans planned and implemented) were measured in only one way (namely, via the self-report on the website), p. 20.  PN: The outcome (daily step count) was also measured in only one possible way (and that was via a pedometer), p. 16. | Y / PY / PN / N / NI |
| 5.3 ... multiple eligible analyses of the data? | PN: Only one method of analysis is reported to analyze the outcome (action plans planned and implemented) (mean and standard deviations and Cohen's d to examine the effect of condition on the average number of action plans), p. 18.  PN: Also, only one method of analysis is reported for the outcome daily steps (mean and standard deviations for both groups and an RM-ANOVA to find differences between the two groups in the pre-incentive (Phase I), incentive (Phases II and III), and post-incentive (Phases IV and V) periods), p. 18. | Y / PY / PN / N / NI |
| Risk-of-bias judgement | some concerns | low / high / some concerns |
| Optional: What is the predicted direction of bias due to selection of the reported result? | - | NA / Favours experimental / Favours comparator / Towards null /Away from null / Unpredictable |

Appendix 3. Tab. 44. Overall risk of bias

| Risk-of-bias judgement | high risk | low / high / some concerns |
| --- | --- | --- |
| Optional: What is the overall predicted direction of bias for this outcome? | - | NA / Favours experimental / Favours comparator / Towards null /Away from null / Unpredictable |

Assessment of risk of bias (using RoB 2) – Patel et al. (2016)

Appendix 3. Tab. 45. Study details

| \| Reference \| Patel, M.S.; Asch, D.A.; Rosin, R.; Small, D.S.; Bellamy, S.L.; Heuer, J.; Sproat, S.; Hyson, C.; Haff, N.; Lee, S.M.; Wesby, L.; Hoffer, K.; Shuttleworth, D.; Taylor, D.H.; Hilbert, V.; Zhu, J.; Yang, L.; Wang, X. & Volpp, K.G. (2016). Framing financial incentives to increase physical activity among overweight and obese adults: a randomized, controlled trial. Ann Intern Med., 164 (6), p. 385–394. \| \| --- \| --- \|   Study design   \| X \| Individually-randomized parallel-group trial \| \| --- \| --- \| \| □ \| Cluster-randomized parallel-group trial \| \| □ \| Individually randomized cross-over (or other matched) trial \|   For the purposes of this assessment, the interventions being compared are defined as   \| Experimental: \| Intervention group: daily feedback + financial incentive. \| Comparator: \| Control group: daily feedback \| \| --- \| --- \| --- \| --- \|  \| Specify which outcome is being assessed for risk of bias \| Daily step count \| \| --- \| --- \|  \| Specify the numerical result being assessed. In case of multiple alternative analyses being presented, specify the numeric result (e.g. RR = 1.52 (95 % CI 0.83 to 2.77) and/or a reference (e.g. to a table, figure or paragraph) that uniquely defines the result being assessed. \| Difference between IG vs. CG (Tab. 2, p. 18)  After 13 weeks: 406 [-380; 1193]; p=0.31)  After 26 weeks: 30 [-741; 802]; p=0.94) \| \| --- \| --- \|   Is the review team’s aim for this result…?   \| X \| to assess the effect of *assignment to intervention* (the ‘intention-to-treat’ effect) \| \| --- \| --- \| \| □ \| to assess the effect of *adhering to intervention* (the ‘per-protocol’ effect) \|   If the aim is to assess the effect of *adhering to intervention*, select the deviations from intended intervention that should be addressed (at least one must be checked):  □ occurrence of non-protocol interventions  □ failures in implementing the intervention that could have affected the outcome  □ non-adherence to their assigned intervention by trial participants  Which of the following sources were obtained to help inform the risk-of-bias assessment? (tick as many as apply)  X Journal article(s) with results of the trial  □ Trial protocol  □ Statistical analysis plan (SAP)  □ Non-commercial trial registry record (e.g. ClinicalTrials.gov record)  □ Company-owned trial registry record (e.g. GSK Clinical Study Register record)  □ “Grey literature” (e.g. unpublished thesis)  □ Conference abstract(s) about the trial  □ Regulatory document (e.g. Clinical Study Report, Drug Approval Package)  □ Research ethics application  □ Grant database summary (e.g. NIH RePORTER or Research Councils UK Gateway to Research)  □ Personal communication with trialist  □ Personal communication with the sponsor |
| --- | --- | --- | --- | --- | --- | --- | --- | --- | --- | --- | --- | --- | --- | --- | --- | --- | --- | --- | --- | --- |

Appendix 3. Tab. 46. Domain 1: Risk of bias arising from the randomization process

| **Signalling questions** | **Comments** | **Response options** |
| --- | --- | --- |
| 1.1 Was the allocation sequence random? | PY: “Participants were electronically randomly assigned to the control group or to 1 of 3 intervention groups (…)”, p. 5  PY: “Participants enrolled online using Way to Health, an automated technology platform based at the University of Pennsylvania that integrates wireless devices, conducts clinical trial randomization and enrollment processes (…)”, p. 4 | Y / PY / PN / N / NI |
| 1.2 Was the allocation sequence concealed until participants were enrolled and assigned to interventions? |  | Y / PY / PN / N / NI |
| 1.3 Did baseline differences between intervention groups suggest a problem with the randomization process? | PN: “Participant baseline characteristics were generally well-balanced across the 4 study groups (Table 1) (…)” p. 7  Report of baseline values without p-values | Y / PY / PN / N / NI |
| Risk-of-bias judgement | low risk | low / high / some concerns |
| Optional: What is the predicted direction of bias arising from the randomization process? | - | NA / Favours experimental / Favours comparator / Towards null /Away from null / Unpredictable |

Appendix 3. Tab. 47. Domain 2: Risk of bias due to deviations from the intended interventions (effect of assignment to intervention)

| **Signalling questions** | **Comments** | **Response options** |
| --- | --- | --- |
| 2.1. Were participants aware of their assigned intervention during the trial? | Y: “Neither the participants nor the study coordinator could be blinded to the group assignment.”, p. 5 | Y / PY / PN / N / NI |
| 2.2. Were carers and people delivering the interventions aware of participants' assigned intervention during the trial? |  | Y / PY / PN / N / NI |
| 2.3. If Y/PY/NI to 2.1 or 2.2: Were there deviations from the intended intervention that arose because of the trial context? | NI: No deviations from the planned intervention can be identified from the study report. However, no study protocol is available and thus not enough information, so that this question cannot be answered conclusively. | NA / Y / PY / PN / N / NI |
| 2.4 If Y/PY to 2.3: Were these deviations likely to have affected the outcome? | - | NA / Y / PY / PN / N / NI |
| 2.5. If Y/PY/NI to 2.4: Were these deviations from intended intervention balanced between groups? | - | NA / Y / PY / PN / N / NI |
| 2.6 Was an appropriate analysis used to estimate the effect of assignment to intervention? | Y: “All other randomly assigned participants were included in the intention-to-treat analysis.”, p. 5 | Y / PY / PN / N / NI |
| 2.7 If N/PN/NI to 2.6: Was there potential for a substantial impact (on the result) of the failure to analyse participants in the group to which they were randomized? | - | NA / Y / PY / PN / N / NI |
| Risk-of-bias judgement | some concerns | low / high / some concerns |
| Optional: What is the predicted direction of bias due to deviations from intended interventions? | - | NA / Favours experimental / Favours comparator / Towards null /Away from null / Unpredictable |

Appendix 3. Tab. 48. Domain 2: Risk of bias due to deviations from the intended interventions (effect of adhering to intervention)

| **Signalling questions** | **Comments** | **Response options** |
| --- | --- | --- |
| 2.1. Were participants aware of their assigned intervention during the trial? | - | Y / PY / PN / N / NI |
| 2.2. Were carers and people delivering the interventions aware of participants' assigned intervention during the trial? |  | Y / PY / PN / N / NI |
| 2.3. [If applicable:] If Y/PY/NI to 2.1 or 2.2: Were important non-protocol interventions balanced across intervention groups? | - | NA / Y / PY / PN / N / NI |
| 2.4. [If applicable:] Were there failures in implementing the intervention that could have affected the outcome? | - | NA / Y / PY / PN / N / NI |
| 2.5. [If applicable:] Was there non-adherence to the assigned intervention regimen that could have affected participants’ outcomes? | - | NA / Y / PY / PN / N / NI |
| 2.6. If N/PN/NI to 2.3, or Y/PY/NI to 2.4 or 2.5: Was an appropriate analysis used to estimate the effect of adhering to the intervention? | - | NA / Y / PY / PN / N / NI |
| Risk-of-bias judgement | - | low / high / some concerns |
| Optional: What is the predicted direction of bias due to deviations from intended interventions? | - | NA / Favours experimental / Favours comparator / Towards null /Away from null / Unpredictable |

Appendix 3. Tab. 49. Domain 3: Risk of bias due to missing outcome data

| **Signalling questions** | **Comments** | **Response options** |
| --- | --- | --- |
| 3.1 Were data for this outcome available for all, or nearly all, participants randomized? | “For continuous outcomes, availability of data from 95 % of the participants will often be sufficient.”, (Higgins et al., 2019)*  Y: For the results of the weeks 1-13: “In all 4 groups, at least 95 % of the participants completed the 13-week intervention (…).”, p. 7  N: For the results of the weeks 14-26: “In all 4 groups, (…) at least 92 % completed the entire 26-week study.”, p. 7 | Y / PY / PN / N / NI |
| 3.2 If N/PN/NI to 3.1: Is there evidence that the result was not biased by missing outcome data? | Y: “For all outcomes, results of sensitivity analyses adjusted by device and using different methods of accounting for missing data were qualitatively similar to those of the main model.”, p. 7 | NA / Y / PY / PN / N |
| 3.3 If N/PN to 3.2: Could missingness in the outcome depend on its true value? | - | NA / Y / PY / PN / N / NI |
| 3.4 If Y/PY/NI to 3.3: Is it likely that missingness in the outcome depended on its true value? |  | NA / Y / PY / PN / N / NI |
| Risk-of-bias judgement | low risk | low / high / some concerns |
| Optional: What is the predicted direction of bias due to missing outcome data? | - | NA / Favours experimental / Favours comparator / Towards null /Away from null / Unpredictable |
| *Source: Higgins, J. P. T., Savović, J., Page, M. J., & Sterne, J. A. C. (Eds.). (2019a). Revised Cochrane risk-of-bias tool for randomized trials (RoB 2). Retrieval from https://drive.google.com/file/d/19R9savfPdCHC8XLz2iiMvL_71lPJERWK/view?pli=1 | | |

Appendix 3. Tab. 50. Domain 4: Risk of bias in measurement of the outcome

| **Signalling questions** | **Comments** | **Response options** |
| --- | --- | --- |
| 4.1 Was the method of measuring the outcome inappropriate? | N: The following procedure was performed: “Step counts were tracked using the Moves smart-phone application (ProtoGeo Oy), which uses accelerometers within the phone and has been shown by our prior work to be accurate. Each participant was given a unique personal identification number to enter into the smartphone application and verify permission that the study team could access step-count data. Once the application was installed on the phone, the participant never had to reopen it, although they could as often as they wished. Instead, participants had to allow the application to run passively on the phone, have the phone powered on, and carry it with them (for example, in a pocket or on a belt clip or arm band) while they were active.”, p. 4 | Y / PY / PN / N / NI |
| 4.2 Could measurement or ascertainment of the outcome have differed between intervention groups? | N: The steps of all participants were measured with a pedometer. | Y / PY / PN / N / NI |
| 4.3 If N/PN/NI to 4.1 and 4.2: Were outcome assessors aware of the intervention received by study participants? | N: “All investigators, statisticians, and data analysts were blinded to group assignments until the 26-week study ended.”, p. 5 | NA / Y / PY / PN / N / NI |
| 4.4 If Y/PY/NI to 4.3: Could assessment of the outcome have been influenced by knowledge of intervention received? | - | NA / Y / PY / PN / N / NI |
| 4.5 If Y/PY/NI to 4.4: Is it likely that assessment of the outcome was influenced by knowledge of intervention received? |  | NA / Y / PY / PN / N / NI |
| Risk-of-bias judgement | low risk | low / high / some concerns |
| Optional: What is the predicted direction of bias in measurement of the outcome? | - | NA / Favours experimental / Favours comparator / Towards null /Away from null / Unpredictable |

Appendix 3. Tab. 51. Domain 5: Risk of bias in selection of the reported result

| **Signalling questions** | **Comments** | **Response options** |
| --- | --- | --- |
| 5.1 Were the data that produced this result analysed in accordance with a pre-specified analysis plan that was finalized before unblinded outcome data were available for analysis? | NI: Due to the fact that the study protocol is not available, no statements can be made as to whether the outcome measurements and analyses performed were planned and determined beforehand. Furthermore, it cannot be ensured that the analysis intentions were completed before the outcome data were available. | Y / PY / PN / N / NI |
| Is the numerical result being assessed likely to have been selected, on the basis of the results, from... |  |  |
| 5.2. ... multiple eligible outcome measurements (e.g. scales, definitions, time points) within the outcome domain? | NI due to the lack of a study protocol . | Y / PY / PN / N / NI |
| 5.3 ... multiple eligible analyses of the data? | NI due to the lack of a study protocol | Y / PY / PN / N / NI |
| Risk-of-bias judgement | some concerns | low / high / some concerns |
| Optional: What is the predicted direction of bias due to selection of the reported result? |  | NA / Favours experimental / Favours comparator / Towards null /Away from null / Unpredictable |

Appendix 3. Tab. 52. Overall risk of bias

| Risk-of-bias judgement | some concerns | low / high / some concerns |
| --- | --- | --- |
| Optional: What is the overall predicted direction of bias for this outcome? | - | NA / Favours experimental / Favours comparator / Towards null /Away from null / Unpredictable |

Assessment of risk of bias (using RoB 2) – Royer et al. (2015)

Appendix 3. Tab. 53. Study details

| \| Reference \| Royer, H.; Stehr, M. & Sydnor, J. (2015). Incentives, Commitments, and Habit Formation in Exercise: Evidence from a Field Experiment with Workers at a Fortune-500 Company. American Economic Journal: Applied Economics, 7(3), p. 51–84. \| \| --- \| --- \|   Study design   \| X \| Individually-randomized parallel-group trial \| \| --- \| --- \| \| □ \| Cluster-randomized parallel-group trial \| \| □ \| Individually randomized cross-over (or other matched) trial \|   For the purposes of this assessment, the interventions being compared are defined as   \| Experimental: \| Intervention group: incentives \| Comparator: \| Control group \| \| --- \| --- \| --- \| --- \|  \| Specify which outcome is being assessed for risk of bias \| Gym visits (gym visits measured as "Any Visit" = at least 1 gym visit per week). \| \| --- \| --- \|  \| Specify the numerical result being assessed. In case of multiple alternative analyses being presented, specify the numeric result (e.g. RR = 1.52 (95 % CI 0.83 to 2.77) and/or a reference (e.g. to a table, figure or paragraph) that uniquely defines the result being assessed. \| Gym visits for all participants (Tab. 2, p. 66)  Week 1-4: 0.18 (0.02)  week 5-13: 0.04 (0.02)  Week 14-26: 0.04 (0.02)  Gym visits among non-members (Table 2, p. 66).  Week 1-4: 0.15 (0.02)  Week 5-13: 0.04 (0.02)  Week 14-26: 0.03 (0.02)  Gym visits among members (Tab. 2, p. 66).  Week 1-4: 0.23 (0.04)  Week 5-13: 0.03 (0.03)  Week 14-26: 0.04 (0.04) \| \| --- \| --- \|   Is the review team’s aim for this result…?   \| X \| to assess the effect of *assignment to intervention* (the ‘intention-to-treat’ effect) \| \| --- \| --- \| \| □ \| to assess the effect of *adhering to intervention* (the ‘per-protocol’ effect) \|   If the aim is to assess the effect of *adhering to intervention*, select the deviations from intended intervention that should be addressed (at least one must be checked):  □ occurrence of non-protocol interventions  □ failures in implementing the intervention that could have affected the outcome  □ non-adherence to their assigned intervention by trial participants  Which of the following sources were obtained to help inform the risk-of-bias assessment? (tick as many as apply)  X Journal article(s) with results of the trial  □ Trial protocol  □ Statistical analysis plan (SAP)  □ Non-commercial trial registry record (e.g. ClinicalTrials.gov record)  □ Company-owned trial registry record (e.g. GSK Clinical Study Register record)  □ “Grey literature” (e.g. unpublished thesis)  □ Conference abstract(s) about the trial  □ Regulatory document (e.g. Clinical Study Report, Drug Approval Package)  □ Research ethics application  □ Grant database summary (e.g. NIH RePORTER or Research Councils UK Gateway to Research)  □ Personal communication with trialist  □ Personal communication with the sponsor |
| --- | --- | --- | --- | --- | --- | --- | --- | --- | --- | --- | --- | --- | --- | --- | --- | --- | --- | --- | --- | --- |

Appendix 3. Tab. 54. Domain 1: Risk of bias arising from the randomization process

| **Signalling questions** | **Comments** | **Response options** |
| --- | --- | --- |
| 1.1 Was the allocation sequence random? | NI: The only information about the randomization method is that the study was randomized.  NI | Y / PY / PN / N / NI |
| 1.2 Was the allocation sequence concealed until participants were enrolled and assigned to interventions? |  | Y / PY / PN / N / NI |
| 1.3 Did baseline differences between intervention groups suggest a problem with the randomization process? | N: No irregularities can be found with regard to the descriptive characteristics of the participants at the beginning of the study. Tab. 1a+b, p. 60+61 (“Overall, the groups are fairly well balanced across the different treatments; none of the pretreatment differences examined in Table 1 are statistically different from zero at the 5 percent level.”, p. 60) | Y / PY / PN / N / NI |
| Risk-of-bias judgement | some concerns | low / high / some concerns |
| Optional: What is the predicted direction of bias arising from the randomization process? | - | NA / Favours experimental / Favours comparator / Towards null /Away from null / Unpredictable |

Appendix 3. Tab. 55. Domain 2: Risk of bias due to deviations from the intended interventions (effect of assignment to intervention)

| **Signalling questions** | **Comments** | **Response options** |
| --- | --- | --- |
| 2.1. Were participants aware of their assigned intervention during the trial? | PY: No information about it is provided. However, the participants need the information about the incentive, because this is supposed to motivate them to visit the gym.  NI | Y / PY / PN / N / NI |
| 2.2. Were carers and people delivering the interventions aware of participants' assigned intervention during the trial? |  | Y / PY / PN / N / NI |
| 2.3. If Y/PY/NI to 2.1 or 2.2: Were there deviations from the intended intervention that arose because of the trial context? | NI: No deviations from the planned intervention can be identified from the study report. However, no study protocol is available and thus not enough information, so that this question cannot be answered conclusively. | NA / Y / PY / PN / N / NI |
| 2.4 If Y/PY to 2.3: Were these deviations likely to have affected the outcome? | - | NA / Y / PY / PN / N / NI |
| 2.5. If Y/PY/NI to 2.4: Were these deviations from intended intervention balanced between groups? | - | NA / Y / PY / PN / N / NI |
| 2.6 Was an appropriate analysis used to estimate the effect of assignment to intervention? | PN: An ITT analysis is performed. However, this appears to have been conducted in a different context with data on the intervention with commitment. (“The actual IV estimates are generally around 0.5, suggesting that the intention-to-treat effect sizes we observe here are broadly sensible.”, p. 67) | Y / PY / PN / N / NI |
| 2.7 If N/PN/NI to 2.6: Was there potential for a substantial impact (on the result) of the failure to analyse participants in the group to which they were randomized? | PN: There is no evidence that there were participants who were analyzed in the wrong group and thus may have biased the results. | NA / Y / PY / PN / N / NI |
| Risk-of-bias judgement | some concerns | low / high / some concerns |
| Optional: What is the predicted direction of bias due to deviations from intended interventions? | - | NA / Favours experimental / Favours comparator / Towards null /Away from null / Unpredictable |

Appendix 3. Tab. 56. Domain 2: Risk of bias due to deviations from the intended interventions (effect of adhering to intervention)

| **Signalling questions** | **Comments** | **Response options** |
| --- | --- | --- |
| 2.1. Were participants aware of their assigned intervention during the trial? | - | Y / PY / PN / N / NI |
| 2.2. Were carers and people delivering the interventions aware of participants' assigned intervention during the trial? |  | Y / PY / PN / N / NI |
| 2.3. [If applicable:] If Y/PY/NI to 2.1 or 2.2: Were important non-protocol interventions balanced across intervention groups? | - | NA / Y / PY / PN / N / NI |
| 2.4. [If applicable:] Were there failures in implementing the intervention that could have affected the outcome? | - | NA / Y / PY / PN / N / NI |
| 2.5. [If applicable:] Was there non-adherence to the assigned intervention regimen that could have affected participants’ outcomes? | - | NA / Y / PY / PN / N / NI |
| 2.6. If N/PN/NI to 2.3, or Y/PY/NI to 2.4 or 2.5: Was an appropriate analysis used to estimate the effect of adhering to the intervention? | - | NA / Y / PY / PN / N / NI |
| Risk-of-bias judgement | - | low / high / some concerns |
| Optional: What is the predicted direction of bias due to deviations from intended interventions? | - | NA / Favours experimental / Favours comparator / Towards null /Away from null / Unpredictable |

Appendix 3. Tab. 57. Domain 3: Risk of bias due to missing outcome data

| **Signalling questions** | **Comments** | **Response options** |
| --- | --- | --- |
| 3.1 Were data for this outcome available for all, or nearly all, participants randomized? | N: Follow-up rates ranged from 89.8% to 94.8%, depending on the group. (see Online Appendix, Figure 2)  According to Higgins et al. (2019)*, an availability of at least 95% of the data is to be classified as "nearly all". | Y / PY / PN / N / NI |
| 3.2 If N/PN/NI to 3.1: Is there evidence that the result was not biased by missing outcome data? | PN: No information is provided on analysis methods that would indicate whether missing outcome data bias the results. | NA / Y / PY / PN / N |
| 3.3 If N/PN to 3.2: Could missingness in the outcome depend on its true value? | NI: No information is provided on why participants dropped out. This also makes it impossible to find out whether their dropout is related to the study content.  NI: no reportet reasons for missing outcome data | NA / Y / PY / PN / N / NI |
| 3.4 If Y/PY/NI to 3.3: Is it likely that missingness in the outcome depended on its true value? |  | NA / Y / PY / PN / N / NI |
| Risk-of-bias judgement | high risk | low / high / some  concerns |
| Optional: What is the predicted direction of bias due to missing outcome data? | - | NA / Favours experimental / Favours comparator / Towards null /Away from null / Unpredictable |
| *Source: Higgins, J. P. T., Savović, J., Page, M. J., & Sterne, J. A. C. (Eds.). (2019a). Revised Cochrane risk-of-bias tool for randomized trials (RoB 2). Retrieval from https://drive.google.com/file/d/19R9savfPdCHC8XLz2iiMvL_71lPJERWK/view?pli=1 | | |

Appendix 3. Tab. 58. Domain 4: Risk of bias in measurement of the outcome

| **Signalling questions** | **Comments** | **Response options** |
| --- | --- | --- |
| 4.1 Was the method of measuring the outcome inappropriate? | N: “Upon entry to the gym, employees log in at a computer terminal and these computerized log-ins serve as our primary data.”, p. 55 | Y / PY / PN / N / NI |
| 4.2 Could measurement or ascertainment of the outcome have differed between intervention groups? | N: Gym visits from all participants were recorded via login data to the gym, regardless of group affiliation. | Y / PY / PN / N / NI |
| 4.3 If N/PN/NI to 4.1 and 4.2: Were outcome assessors aware of the intervention received by study participants? | NI: No information about it is provided. | NA / Y / PY / PN / N / NI |
| 4.4 If Y/PY/NI to 4.3: Could assessment of the outcome have been influenced by knowledge of intervention received? | PN: Because the results were collected automatically by the login data, the influence of the knowledge about the group membership can be estimated as low. | NA / Y / PY / PN / N / NI |
| 4.5 If Y/PY/NI to 4.4: Is it likely that assessment of the outcome was influenced by knowledge of intervention received? |  | NA / Y / PY / PN / N / NI |
| Risk-of-bias judgement | low risk | low / high / some  concerns |
| Optional: What is the predicted direction of bias in measurement of the outcome? | - | NA / Favours experimental / Favours comparator / Towards null /Away from null / Unpredictable |

Appendix 3. Tab. 59. Domain 5: Risk of bias in selection of the reported result

| **Signalling questions** | **Comments** | **Response options** |
| --- | --- | --- |
| 5.1 Were the data that produced this result analysed in accordance with a pre-specified analysis plan that was finalized before unblinded outcome data were available for analysis? | NI: Due to the fact that the study protocol is not available, no statements can be made as to whether the outcome measurements and analyses performed were planned and determined beforehand. Furthermore, it cannot be ensured that the analysis intentions were completed before the outcome data were available. | Y / PY / PN / N / NI |
| Is the numerical result being assessed likely to have been selected, on the basis of the results, from... |  |  |
| 5.2. ... multiple eligible outcome measurements (e.g. scales, definitions, time points) within the outcome domain? | PN: Despite the fact that no study protocol is available and therefore no final examination of the outcome measures is possible, all reported results for the outcome gym use seem to be consistent with the intended measurements (see p. 61 et sqq). | Y / PY / PN / N / NI |
| 5.3 ... multiple eligible analyses of the data? | PN: Due to the lack of a study protocol, no firm statements can be made about the analysis intentions. However, the study report reports quite extensively on how the results are to be analyzed (see p. 64, et sqq.) | Y / PY / PN / N / NI |
| Risk-of-bias judgement | some concerns | low / high / some concerns |
| Optional: What is the predicted direction of bias due to selection of the reported result? | - | NA / Favours experimental / Favours comparator / Towards null /Away from null / Unpredictable |

Appendix 3. Tab. 60. Overall risk of bias

| Risk-of-bias judgement | high risk | low / high / some concerns |
| --- | --- | --- |
| Optional: What is the overall predicted direction of bias for this outcome? | - | NA / Favours experimental / Favours comparator / Towards null /Away from null / Unpredictable |

Appendix 3. Tab. 61. Impact of risk of bias on quality of evidence

| **Criteria** | **Assessment of the studies** |
| --- | --- |
| Lack of allocation concealment | low risk: Finkelstein et al. (2016), Omran et al. (2018), Patel et al. (2016), Hunter et al. (2013)  some concerns (no information): Carrera et al. (2020), Royer et al. (2015) |
| Lack of blinding | low risk: Finkelstein et al. (2016), Hunter (2013)  some concerns: Carrera et al. (2020), Omran et al. (2018), Patel et al. (2016), Royer et al. (2015) |
| Incomplete accounting of patients and outcome events | low risk: Finkelstein et al. (2016), Patel et al. (2016)  high risk: Carrera et al. (2020), Omran et al. (2018), Royer et al. (2015)  no information: Hunter et al. (2013) |
| Selective outcome reporting | low risk: Finkelstein et al. (2016), Hunter et al. (2013)  some concerns (no information): Carrera et al. (2020), Omran et al. (2018), Patel et al. (2016), Royer et al. (2015) |
| Other limitations | some concerns (regarding the use of non-validated outcome measures (outcomes reported by patients)): Omran et al. (2018) |
